# The Discovery of Complex Heterocycles from Millipede Secretions

**DOI:** 10.1021/jacs.5c08079

**Published:** 2025-07-17

**Authors:** Paige Banks, Carla Menegatti, Lin Du, Paul E. Marek, Emily Mevers

**Affiliations:** † Department of Chemistry, 1757 Virginia Tech, Blacksburg, Virginia 24061, United States; ‡ Molecular Targets Program, Center for Cancer Research, 70717 National Cancer Institute, Frederick, Maryland 24701, United States; § Department of Entomology, Virginia Tech, Blacksburg, Virginia 24061, United States

## Abstract

The recent explosion in documented chemistry from Colobognatha, a millipede subterclass with more than 240 species, has rekindled interest in the defensive secretions from these ancient animals. Prior to 2020, studies on defensive secretions by Colobognatha—the only millipedes that produce terpenoid alkaloids—were limited to a single order, Polyzoniida. However, numerous species of the order Platydesmida have recently been shown to produce structurally diverse terpenoid alkaloids with potent biological activities. Platydesmidan defensive secretions encompass multiple natural product scaffolds with greater chemical complexity compared to previously reported millipede-derived alkaloids. Here, we report an analysis of the defensive secretions of , an evolutionary sister to all other Platydesmida. Analyzing defensive secretions revealed that produces an arsenal of alkaloids dissimilar to all previously reported metabolites. Using various analytical techniques, we accomplished complete structural assignment of two distinct scaffolds: a 5,6-fused heterocycle named the andrognathines and a 6,6,6,5-bridged heterocycle containing seven continuous stereogenic centers named the andrognathanols. Each scaffold contains diverse fatty acids; this leads to an extraordinary number of unique metabolites. These described alkaloids are actively secreted upon physical disturbance and change the behavior of ants that reside in the same environment.

## Graphical abstract

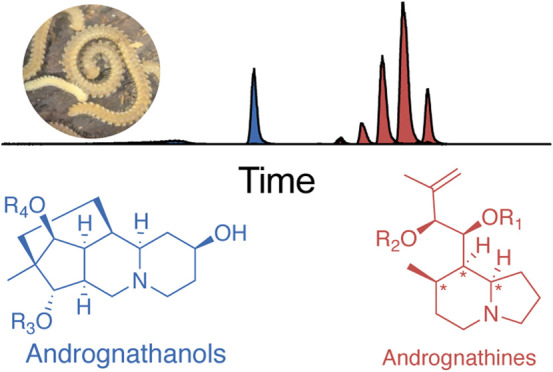

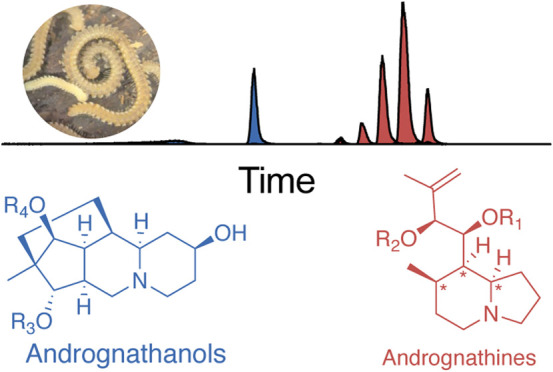

## Introduction

A single subterclass, the Colobognatha, produces all millipede-derived terpenoid alkaloids. This monophyletic group consists of four species-rich orders. Unlike other millipedes, colobognaths possess unique characteristics—feeding primarily on fungus, forming large aggregations, and exhibiting other social behaviors such as parental egg care.^1,2^ The size of these aggregations varies greatly, and their function remains unclear, but some contain over 100 millipedes.^3^ Despite the fascinating biology observed in Colobognatha, few studies have focused on the chemical composition of their defensive secretions. Prior to 2020, only three terpenoid alkaloids—buzonamine (), polyzonimine (*Petaserpes rosalbus*) and spiropyrrolizidine *O*-methyloxime 236 ()—were reported, and all from defensive secretions of species within a single order, Polyzoniida.^4–7^ Very few studies examine millipedes from the other three orders of Colobognatha; in those limited studies, only simple monoterpenes appear.^8^ However, recent findings indicate a proliferation in new chemistry from species of Platydesmida.^9–11^ First, in 2020 and 2024, , , and secretions were determined to contain indolizidine and quinolizidines.^10,12^ Next, in 2022 and 2024, and were shown to produce the deoxybuzonamine isomers.^10,11^ Finally, in 2025, secretions were reported to contain the oxidized ischnocybines.^9^

The ecological role of terpenoid alkaloids remains unclear; however, they most likely serve a defensive role, act as pheromones, or function in both capacities.^13,14^ Like nearly all millipedes, Colobognatha have evolved specialized repugnatorial glands that store and secrete defensive compounds. These glands open through ozopores and are located bilaterally, with two glands per segment.^8^ Some species of Colobognatha possess up to 330 segments.^15^ Several alkaloids (*e.g.,* glomerin, polyzonimine, buzonamine, and the ischnocybines) deter commonly encountered predators (*e.g.,* spiders, cockroaches, flies, and ants).^8,16–19^ However, only the ischnocybines and spiropyrrolizidines have been evaluated in a range of receptor assays. Ischnocybine A potently binds the sigma-1 receptor (σ_1_R), an orphan neuroreceptor, with 100-fold selectivity over the sigma-2 receptor (σ_2_R). Molecular docking experiments suggest the ischnocybines act as σ_1_R antagonists.^9^ Conversely, the spiropyrrolizidine oximes appear to block nicotinic receptors with selectivity for ganglionic-type receptors.^20^ Additionally, both spiropyrrolizidine oximes and polyzonimine have been isolated from the skin of poison dart frogs—researchers hypothesize that the frogs acquire these alkaloids through their diet for defensive purposes.^21^ Given the social characteristics displayed by Colobognatha, terpenoid alkaloids might function as pheromones, aiding in forming large aggregations and brood care.^8^

To date, the defense secretion composition from Platydesmida species shows the greatest structural diversity with the production of two distinct core structures: a 5,6,6-fused heterocycle and an indolizidine/quinolizidine core.^10^ In addition, Platydesmida secretions contain significantly more predicted post-modification events (*e.g.,* multiple oxidations, ligation events, and incorporation of different precursors).^9–11,20^ Yet, chemists have studied less than 10% of colobognath species, with several entire genera unstudied. This includes , a living member of one of the oldest genera of the Platydesmida with a fossil record dated to the Late Cretaceous.^13,22^ Understanding the structural diversity of these unstudied genera will lead to a better understanding of their true ecological role.

## Results and Discussion

### Chemical Investigations into Defensive Secretions of 

 specimens were primarily collected in decayed woody debris in southwestern Virginia (Table S1) between May and September, with large aggregations commonly found from late May through July. Field observations across multiple years suggested that these large aggregations were more active toward the beginning of August, as pairs of adult millipedes and juveniles were more frequently encountered. By October, locating any millipedes on the same woody debris became increasingly difficult. This led to the hypothesis that spends winter underground, potentially protecting itself from cold temperatures and low humidity.

Analysis of the defensive secretions from collected millipedes by high-resolution liquid chromatography mass spectrometry (HR-LCMS) revealed that produces an array of diverse alkaloids (>18 compounds), with molecular ions ranging from 240.1965 to 424.3053 Da (Figure 1). Tandem MS analysis revealed two different structural classes, as indicated by key fragment ions (232.1695 and 204.1753 Da), which are structurally distinct from all previously reported colobognath alkaloids. The alkaloids were purified using reverse-phase HPLC-MS, yielding andrognathines A–F (**1–12**) and andrognathanols A–D (**13**–**18**).

**Figure 1:**
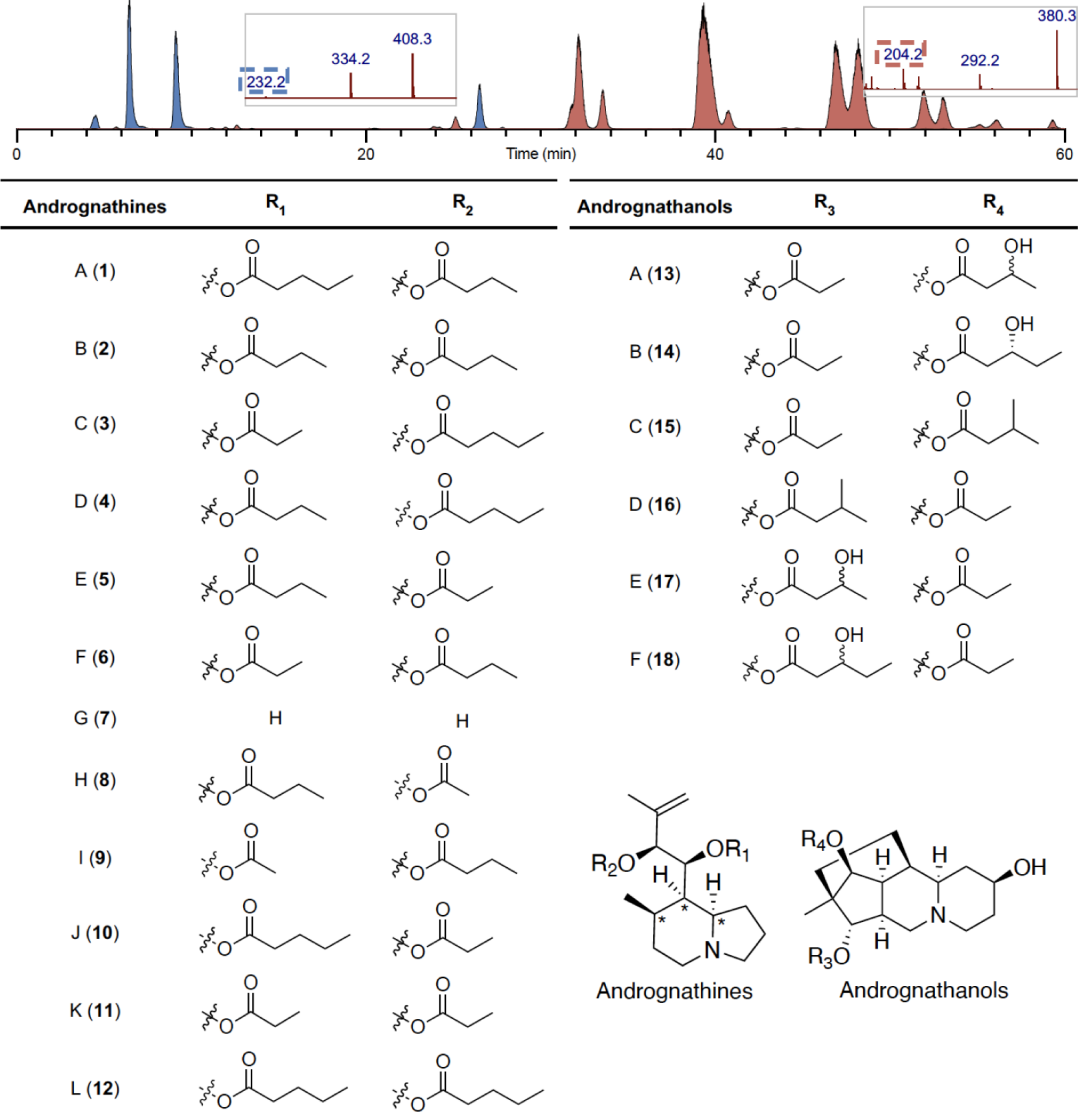
HPLC-MS chromatogram of a representative crude extract. The andrognathanols are highlighted in blue, and the andrognathines are highlighted in red, with key tandem MS fragments shown in the respective boxes. Structures of the andrognathines (**1**–**12**) and andrognathanols (**13**–**18**) from . *Denotes relative configuration.

### Structural Assignment of the Andrognathines (**1–12**)

Full 2D NMR data sets (^1^H, dqfCOSY, gHSQC, H2BC, HMBC and easyROESY) on **1**–**6** (Figures S1–S31 and Tables S2–S7) were acquired. Compound **1** had an [M + H]^+^ of 394.2954 *m*/*z*, suggesting a molecular formula (MF) of C_23_H_39_NO_4_ and indicating five degrees of unsaturation (DoU). Tandem MS analysis of **1** revealed two neutral losses of 88.0523 and 102.0683 Da, suggesting the presence of butyrate and pentanoate esters. ^1^H and ^13^C NMR chemical shifts established two ester carbonyls (δ_C_ 172.3 and 171.8), and an exomethylene (δ_C_ 118.1; δ_H_ 5.10/5.04), accounting for three of the five DoUs. The ^1^H NMR spectrum revealed four methyl groups: three aliphatic (δ_H_ 1.00, 0.87, and 0.86) and one vinyl methyl (δ_H_ 1.70). A comparison of the spectroscopic data to previously reported millipede alkaloids suggested that **1** contained an indolizidine core similar to hydrogosodesmine.^10^ The HSQC spectrum revealed a diagnostic bridgehead methine (δ_C_ 64.3) and diastereomeric methylenes (δ_C_ 53.1 and 51.8) alpha to the tertiary nitrogen, accounting for the remaining two DoUs. However, analysis of H2BC and COSY correlations (Figure 2A) revealed **1** contained a different substitution pattern on the indolizidine ring compared to hydrogosodesmine.

**Figure 2:**
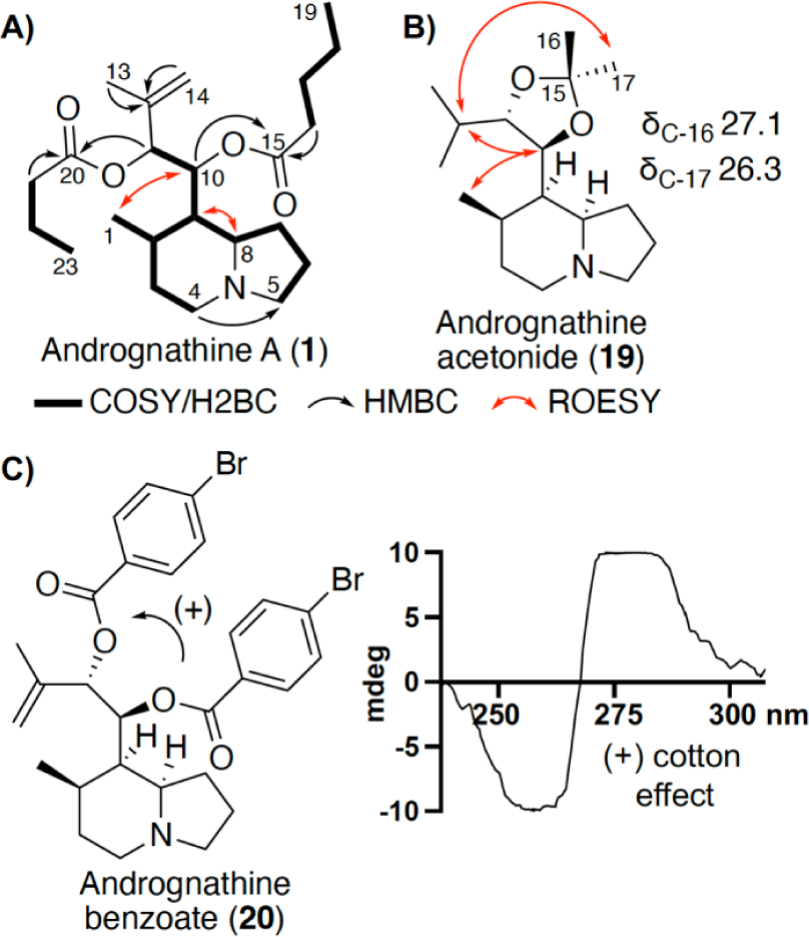
(A) Key 2D NMR correlations for andrognathine A (**1**). (B) Key ROESY correlations and ^13^C NMR chemical shifts of the andrognathine acetonide (**19**). These correlations support a *syn-*relationship between the 10,11-diol. (C) Positive split cotton effect was observed in the ECD spectra of the andrognathine benzoate (**20**), confirming the absolute configuration as 10*S*,11*S*-**20**.

Continuous H2BC correlations from the bridgehead methine (H-8: δ_H_ 1.76) through three diastereomeric methylenes (H-7: δ_H_ 1.53/2.03; H-6: δ_H_ 1.65; H-5: δ_H_ 1.96/2.94) confirmed an unsubstituted pyrrolidine ring. Further analysis of H2BC correlations for H-8 revealed another correlation to a methine, C-9 (δ_C_ 47.1). H-9 (δ_H_ 1.16) exhibited an additional H2BC correlation to another methine (C-2: δ_C_ 32.5) within the indolizidine ring, and an HMBC correlation to an oxygenated methylene (C-10: δ_C_ 70.1), which was positioned on a side chain. To continue establishing the indolizidine system, H-2 (δ_H_ 1.21) had a strong H2BC correlation to a doublet methyl (C-1: δ_C_ 19.8) and a HMBC correlation to diastereomeric methylenes (H-3: δ_H_ 1.21/1.58). H-3 also had a strong H2BC correlation to C-4 (δ_C_ 51.8). The chemical shifts of C-4 are consistent with a carbon alpha to the tertiary amine, thereby establishing a 7,8-disubstituted indolizidine. The oxygenated methine, H-10 (δ_H_ 5.38), previously assigned adjacent to C-9, exhibited a strong HMBC correlation to the other oxygenated carbon, C-11 (δ_C_ 76.1). HMBC correlations from H-11 (δ_H_ 5.45) to the quaternary carbon C-12 (δ_C_ 139.9), the exomethylene C-14 (δ_C_ 118.1), and the singlet vinyl methyl C-13 (δ_C_ 17.5), allowed for assigning the carbon backbone of **1**. The two aliphatic esters were assigned using HMBC correlations: H-10 correlated with C-15 (δ_C_ 172.3), confirming the pentanoate, while H-11 correlated with C-20 (δ_C_ 171.8), confirming the butyrate. This completed the planar structural assignment of **1**, named andrognathine A (Figure 1).

Compounds **2**–**6** had nearly identical NMR data to **1**, but HRMS indicated each had a slightly different MF (**2:** C_22_H_37_NO_4_, **3**: C_22_H_37_NO_4_, **4**: C_23_H_39_NO_4_, **5**: C_21_H_35_NO_4_, and **6**: C_21_H_35_NO_4_). Tandem MS analysis confirmed the presence of a conserved indolizidine core as evidenced by the key 204 Da fragment, while distinct neutral losses distinguished each compound. Structural isomers **2** and **3** differed in their fatty acid composition. Tandem MS data confirmed **2** contained two butyrates, while **3** contained a propionate and a pentanoate, respectively. Compound **4**, a structural isomer of **1**, also had identical neutral losses (88.0526 and 102.0670 Da), indicating incorporation of butyrate and pentanoate, respectively, but in opposite positions compared to **1**. Isomers **5** and **6** displayed neutral losses of 88.0515 and 74.0360 Da, indicating butyrate and propionate, respectively. Analysis of 2D NMR spectra confirmed the planar structures and positions of the side chains for **2**–**6**, andrognathines B–F. There were six additional minor analogs (**7**–**12**), which exhibited the following [M + H]^+^: 240.1969 (**7**), 352.2473 (**8**, **9**, and **11**), 380.2805 (**10**), 408.3114 Da (**12**), that were isolated in too small a quantity for characterization by NMR. Analysis of their neutral losses revealed the assignments as shown in Figure 1 (see Supporting Information).

The relative and absolute configurations of andrognathines were determined through 2D NMR, chemical derivatization, and ECD analysis. First, we assigned the relative configuration among the three adjacent stereocenters within the indolizine portion of **1** through key ROESY correlations (Figure 2A). Correlations between H-1 (δ_H_ 1.00) and H-10 (δ_H_ 5.38), as well as H-8 (δ_H_ 1.76) and H-9 (δ_H_ 1.16), confirmed a *syn* stereochemistry among H-2, H-8, and H-9 in the bicyclic system. However, the flexibility of the side chain attached at C-9 challenged attempts to link the relative configuration of the indolizidine to the side chain. Hydrogenating an aliquot of the crude extract mixture reduced the olefin, hydrolysis removed ester side chains, and subsequent acetonide protection gave one peak in the LCMS with a molecular ion corresponding to **19**. NMR analysis on purified **19** (Figures S32–S37 and Table S8) revealed 1D NOE interactions between H-10 (δ_H_ 4.11) and H-12 (δ_H_ 1.72), H-13 (δ_H_ 0.93), H-14 (δ_H_ 0.84), and H-17 (δ_H_ 1.27), while H-11 (δ_H_ 3.41) had a 1D NOE interaction with the other *gem*-dimethyl within the acetonide (H-16; δ_H_ 1.34). These correlations indicate an *anti*-configuration (Figures 2B and S38). The absolute configuration of C-10 and C-11 was established throughs esterifying a portion of the hydrolyzed diol product with *para*-bromobenzoate to generate **20**. Analysis of **20** by ECD yielded a positive split Cotton effect (Figure 2C), confirming the absolute configuration of the diol as 10*S*,11*S* and establishing 2R*,8R*,9R*,10*S*,11*S* configuration (Figure S38).^23,24^ Unfortunately, the free rotation between C-9 and C-10 hindered the extension of the absolute configuration of the diol to the indolizidine diol. Attempts to use DFT to calculate both ^1^H and ^13^C NMR chemical shifts led to highly similar calculations for both diastereomers. Total synthesis of the andrognathine core will be necessary to deduce the absolute configuration of the indolizidine core.

### Structural Assignment of the Andrognathanols (**13–18**)

Analyzing HR-LCMS data for **13** indicated a molecular ion of [M + H]^+^ 410.2544 *m*/*z*, providing a MF of C_22_H_35_NO_6_, requiring six DoUs. Comparing the 2D NMR data set for **13** (Figures S39–S44 and Table S9) with **1**–**6** revealed significant structural differences, notably the absence of a vinyl methyl and exomethylene. The ^13^C NMR spectrum revealed two esters (δ_C_ 172.9 and 171.1) and four oxygenated carbons (δ_C_ 78.0, 75.4, 67.7, and 63.2). Tandem MS data showed two neutral losses, 104.0488 and 74.0367 Da, suggesting a hydroxybutanoate and propionate, respectively. Analyzing the ^1^H NMR spectrum confirmed the four oxygenated carbons as sp^3^ hybridized with protons at δ_H_ 4.92, 4.60, 4.01, and 3.60, and revealed three aliphatic methyl groups (δ_H_ 0.78, 1.01, and 1.10). However, with no obvious functionality accounting for the additional DoUs, we predicted that **13** contained four rings.

Detailed analysis of the 2D NMR data set for **13** led to the elucidation of the planar structure (Figure 3A). The diagnostic bridgehead methine (Η-8: δ_Η_ 1.90) alpha to the tertiary amine exhibited H2BC correlations to both methylene C-7 (δ_C_ 32.0) and methine C-9 (δ_C_ 30.3). An oxygenated proton H-6 (δ_H_ 4.92) had HMBC correlations to C-8 (δ_C_ 57.6). Additional HMBC correlations from H-6 to methylenes C-5 (δ_C_ 28.8) and C-4 (δ_C_ 49.5), with C-4 being alpha to the tertiary amine, indicated that, unlike in **1**–**6**, an oxygenated piperidine replaced the pyrrolidine ring in andrognathanols. The final methylene alpha to the amine, H-3 (δ_H_ 2.15/2.94), had an H2BC correlation to methine C-2 (δ_C_ 43.4), further distinguishing **13** from **1**–**6**. The H-2 (δ_H_ 1.70) showed two additional H2BC correlations to two methines, including C-10 (δ_C_ 40.8) and C-1 (δ_C_ 75.4), with C-1 being oxygenated. A singlet methyl at δ_Η_ 0.78 exhibited several HMBC correlations, including to C-12 (δ_C_ 42.6), a sp^3^ hybridized quaternary carbon, to C-1, to C-11 (δ_C_ 78.0), and to C-13 (δ_C_ 30.6). This established a 6,6,5-fused tricyclic system. Diastereomeric methylenes, H-13 (δ_H_ 1.14/1.73) and H-14 (δ_H_ 1.30/1.91), exhibited mutual H2BC correlations, while C-14 (δ_C_ 16.9) also correlated with H-9 (δ_H_ 1.49), confirming the connectivity of the final ring. Finally, H2BC correlations between methylene H-20 (δ_H_ 2.37) and oxygenated carbon C-21 (δ_C_ 63.2), as well as C-21 and a doublet methyl, H-22 (δ_H_ 1.10) confirmed a 3-hydroxybutanoate. An HMBC correlation between H-11 (δ_H_ 4.60) and C-19 (δ_C_ 171.1) established the 3-hydroxybutanoate attachment to C-11, while another HMBC correlation between H-1 (δ_H_ 3.60) and C-16 (δ_C_ 172.9) established the propionate attachment to C-16. Thus, we established the planar structure of **13** as a 6,6,6,5-bridged heterocycle as seen in Figure 1.

**Figure 3:**
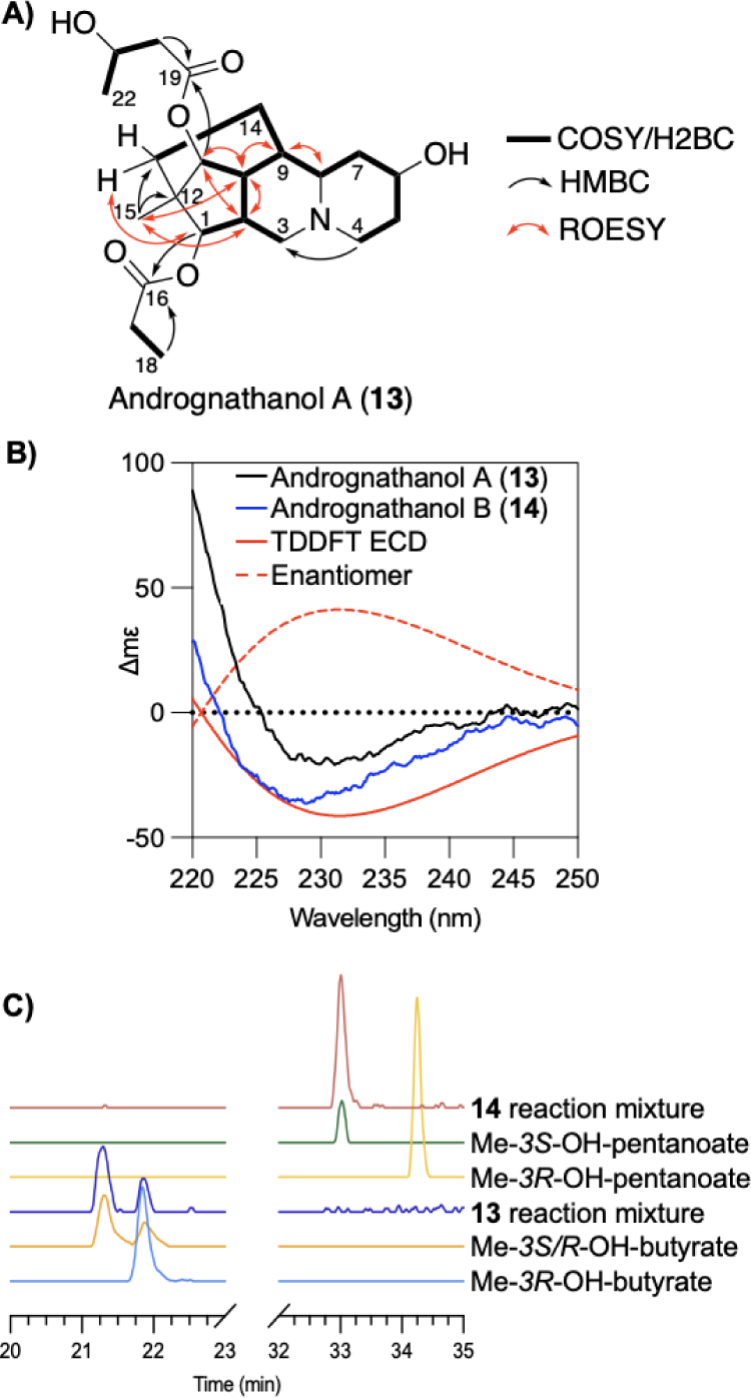
Key 2D NMR correlations for andrognathanol A (**13**). (B) Experimental and TD-DFT data for both **13** and **14** along with predicted data and the enantiomer. (C) Stacked chromatograms of the methylated hydrolyzates of **13** and **14** along with Me–OH-pentanoate and Me–OH-butyrate standards, confirming the absolute configuration as Me-3*S*–OH-pentanoate for **14** and a mixture of Me–OH-butyrate isomers in **13**.

Comparing NMR and tandem MS spectra for **14**–**16** (Figures S45–S60 and Tables S10–S12) with **13** confirmed structural similarities, with all containing the key fragment at 232.1695 Da, indicative of the 6,6,6,5-bridged heterocyclic system. However, like andrognathines, HR-LCMS confirmed that **14**–**16** had different retention times and distinct chemical compositions, C_23_H_37_NO_6_ (**14**), and C_23_H_37_NO_5_ (**15** and **16**), as well as different neutral losses. For **14** tandem MS spectra showed neutral losses of 74.0361 and 118.0645 Da, indicating propionate and hydroxypentanoate, respectively. Analyzing 2D NMR spectra revealed the latter as a 3-hydroxypentanoate attached to C-11. A key HMBC correlation between H-1 (δ_H_ 3.60) and C-16 (δ_C_ 172.9) within the propionate moiety establishes the final connection. Compounds **15** and **16**, structural isomers with identical neutral losses (102.0686 and 74.0362 Da), contained either an isobutyrate or pentanoate and propanoate, respectively. Analyzing 2D NMR confirmed isobutyrate, as evidenced by two doublet methyl groups that exhibited strong HMBC correlations with one another. Key HMBC correlations confirmed the propionate attached to C-11 in **15**, and on C-1 in **16**. In addition, two additional minor analogs (**17** and **18**), which exhibited [M + H]^+^ of 410.2553 and 424.2710 *m*/*z*, were isolated in too small a quantity for characterization by NMR. Analysis of their neutral losses led to the assignment as shown in Figure 1 (see Supporting Information).

We established relative configurations of the andrognathanol A (**13**) by observing key ROESY correlations (Figure 3A). H-11 (δ_H_ 4.60), had ROESY correlations with H-10 (δ_H_ 2.01), H-2 (δ_H_ 1.70), and H-15 (δ_H_ 0.78), indicating these groups are *syn* to one another. In addition, ROESY correlations were observed from H-9 (δ_H_ 1.50) to H-8 (δ_H_ 2.01) and H-10 (δ_H_ 1.57), while H-1 (δ_H_ 3.60) exhibited no ROESY correlations with any stereogenic centers. However, H-1 (δ_H_ 3.57) showed a strong ROESY correlation to H-13a (δ_H_ 1.13), confirming an *anti-*relationship to H-2, H-9, H-10, and H-11.

Finally, the free alcohol form of the andrognathanol core (**21**) was generated by treating the crude extract with 1 N NaOH to remove the side chains. This led to a single peak on LCMS that was purified using RP-HPLC (Figures S61–S66 and Table S13). Acquisition of a 1D NOE spectrum with select irradiation of H-6 (δ_H_ 3.87) revealed a strong correlation to H-8, confirming a *syn* relationship (Figure S66). This allowed for the complete assignment of the relative configuration of the andrognathanol core as 1S*,2S*,6S*,8R*,9R*,10S*,11S*,12S*. Further derivatization of **21** with Mosher’s reagent failed to yield product in high enough quantities for analysis by ^1^H NMR. Therefore, the absolute configuration was established by comparing the experimental ECD spectra to TD-DFT calculated spectra of both enantiomers (Figure 3B). Both **13** and **14** exhibited a negative split Cotton effect, matching the 1*S*,2*S*,6*S*,8*R*,9*R*,10*S*,11*S*,12*S* enantiomer.

The final structural assignment involved determining the absolute configuration of 3-hydroxybutanoate in **13** and 3-hydroxypentanote in **14**. The base hydrolyzate product described above was treated with (trimethylsilyl)­diazomethane to form the methyl esters. Chiral GCMS comparison of the derivatized natural product with authentic standards revealed both enantiomers for both 3-hydroxybutanoate and 3-hydroxypentanoate present in equal quantities (Figure S67). Next, we treated aliquots of purified **13** and **14** with the same conditions and analyzed each. The reaction mixture for **14** contained only methyl-3*S-*hydroxypentanoate (Figure 3C). However, the reaction mixture for **13** still contained a mixture of both enantiomers of methyl-3-hydroxybutanoate (Figure 3C), indicating **13** as a mixture of diastereomers.

### Ecological Role of Secretions

Previous studies on colobognath alkaloids have hypothesized that they may serve a defensive or pheromone role. Experimental trials with potential predators support the former; however, social behaviors of colobognath millipedes, which include forming large aggregations (Figure 4A) and brood care, suggest the latter.^1,8^ When disturbed, many terpenoid alkaloids are actively secreted through ozopores, and a subset appears to deter potential predators, such as cockroaches, flies, and ants.^8,16–18^ However, none of these species have been reported to capture or consume an alkaloid-producing millipede; most are immediately repelled. Our field observations suggest that encounters common predators, such as spiders and ants, as they are typically found on the underside of the same decayed logs. However, when captured and housed in the same enclosure, the millipedes and ants co-occur with one another, cohabitating in discrete locations.

**Figure 4:**
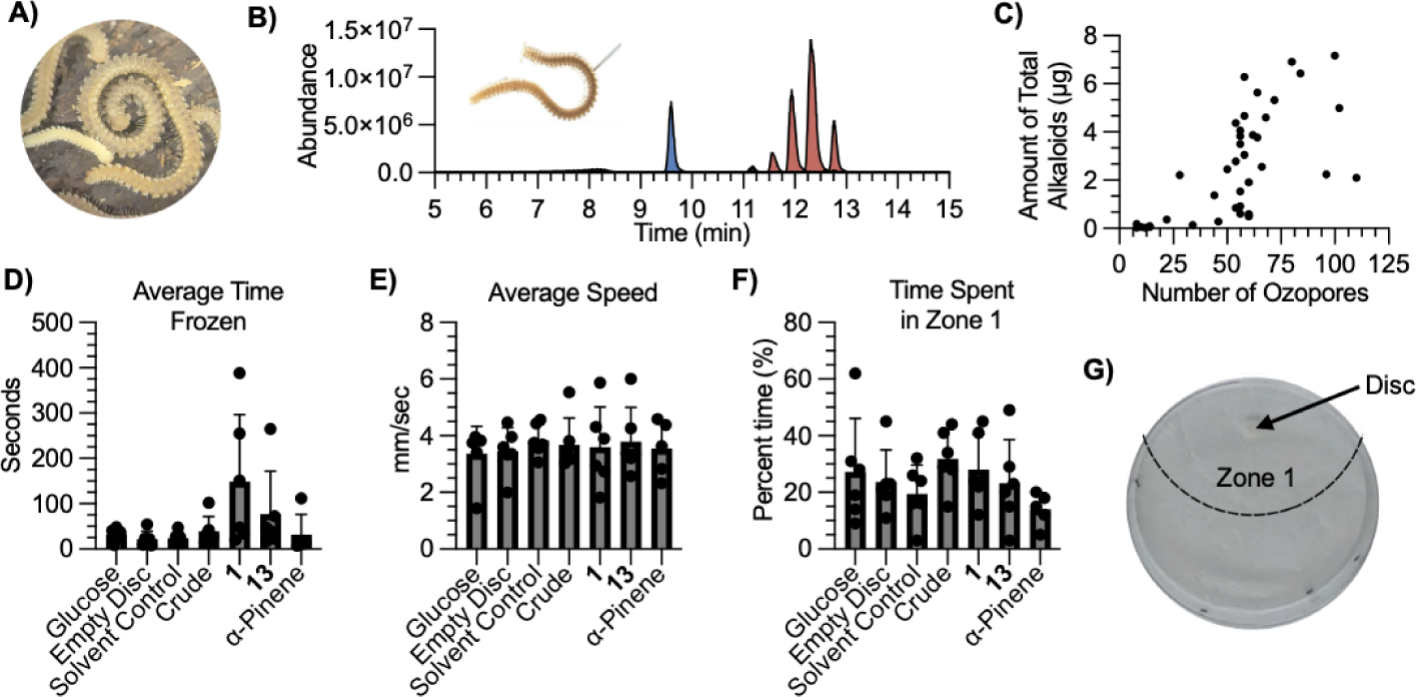
Ecological roles of andrognathines and andrognathanols. (A) aggregation. (B) Chromatogram of a single droplet collected from an ozopore of by capillary. The droplet contains the andrognathanols (blue) and andrognathines (red). (C) The average peak area versus the number of ozopores in an individual millipede shows a positive relationship (R^2^ = 0.75). (D) Average time sp. spent frozen once motion ceased during the predator assay. (E) Average speed of the ants during the predator assay. (F) The percent time each ant spent in zone 1 during the predator assay for each condition. (G) Petri dish during ant assay highlighting zone 1. Error bars represent the standard deviation of the biological replicates (*n* = 5).

To gain an understanding of the ecological role of the , we first confirmed that actively secretes the alkaloids from the ozopores upon disturbance (poking with a capillary). Analyses of captured secretions by LR-LCMS revealed the presence of both the andrognathines and andrognathanols (Figure 4B and Table S14).^3^ Absolute quantification of chemical extractions of 39 single millipedes shows that alkaloid concentration correlates positively with both the number of ozopores (R^2^ = 0.74) and the overall length (R^2^ = 0.75) of each millipede (Figure 4C and S68–S70). This correlation persisted across distinct collection sites in Virginia. In addition, each ozopore contains an average 86 ± 68 ng of the alkaloid mixture, with a mature millipede containing >100 ozopores. Of note, every millipede studied produces significantly more andrognathines than andrognathanols. Finally, we collected millipedes across a larger geographical range: SW North Carolina, SW Virginia, and Central Alabama. All collections contained both the andrognathines and andrognathanols, albeit with some variation in relative abundance (Figure S71).

To evaluate whether these compounds deter common predators, we conducted behavioral assays using sp., an ant frequently encountered in the same habitat as . Individual ants were presented with filter paper discs treated with 400 μg of the crude extract from a single millipede, **1**, **13**, α-pinene, glucose, a solvent control, or an untreated disc. Each assay lasted 15 min, with observations beginning after a 2 min acclimation period to allow the ants to adjust to the testing arena. Analysis of the video using ToxTrac allowed us to quantify 1) the average time each ant spent frozen (Figure 4D), 2) the average speed of each ant (Figure 4E), and 3) the average time spent within 5 cm (zone 1) of the impregnated disc (Figure 4F,G).^25^ None of the treatments had an impact on the ant’s average speed or time spent within zone 1, but the alkaloids did seem to impact the time spent not moving. Specifically, when exposed to the alkaloids, the ants appeared to stop moving more frequently (not quantified), and their stops lasted for a prolonged period compared to the controls. Although this was only statistically significant when the ants were exposed to **1** compared to either the solvent control or the empty disc (*p* = 0.036; *p* = 0.050, respectively), **13** caused the ants to stop moving and trends in the same direction as **1**. Although the ants spend similar amounts of time in zone 1 when exposed to the alkaloids compared to controls, we believe this is partly due to the prolonged stopping induced by exposure to **1** and the other alkaloids. It is worth noting that the ants are likely encountering the alkaloids through direct interactions with the millipedes, which likely affects their behavior. The alkaloids seem to modify the ant’s behavior, but further studies into other predators, including vertebrates (e.g., salamanders), are needed to fully understand their defensive role.

### Biological Activity of the Andrognathines and Andrognathanols

Recent studies have shown that ischnocybines potently inhibit the σ_1_R neuroreceptor (K_i_ 13.1 nM) and exhibit 100-fold selectivity over the σ_2_R.^9^ Due to similar structural features, we evaluated two andrognathines (**1** and **5**) and three andrognathanols (**13**–**15**) against σ_1_R and σ_2_R in collaboration with the PDSP at UNC Chapel Hill (Figures S72 and S73 and Tables S15 and S16). The andrognathines showed mixed results, where **1** exhibited a marked decrease in potency against σ_1_R (K_i_ 838 nM) but maintained a 3-fold selectivity for σ_1_R over σ_2_R (K_i_ 2123 nM), while **5** showed no activity against either receptor. We note that the andrognathines have some stability issues involving the side chain, and both may have partially degraded before the assay. Surprisingly, the more complex andrognathanols (**1–3**) showed no activity against either receptor (K_i_ > 10 μM; Table S17). Structurally, the millipede alkaloids resemble the pumilotoxins, potent voltage-gated sodium channels (VGSCs) inhibitors.^26^ However, **1** and **13** show no activity against two Na_V_ receptors, Na_V_1.5 and 1.8, at concentrations up to 10 μM (Tables S18 and S19).

## Conclusion

Millipede defensive secretions reveal a remarkable evolutionary story, with repugnatorial glands evolving over 385 million years ago and defensive secretions having significantly diversified across the more than 12,000 known species.^27^ Only Colobognatha produce terpenoid alkaloids, a structurally intriguing subset of these defensive secretions. A recent surge in characterizing the secretions by Colobognatha from the order Platydesmida has revealed significant, unexplored novel chemistry. Analytical investigations led to the discovery of the andrognathines and andrognathanols, terpenoid alkaloids structurally distinct from all previously described chemistry. Andrognathanols exhibit unique chemistry, featuring an unprecedented 6,6,6,5-bridged heterocyclic core with seven continuous stereogenic centers. These alkaloids are actively secreted from ozopores when physically agitated, which modifies the behavior of an ant commonly found in the same environment.

## Supplementary Material



## Data Availability

The data underlying this study are available in the published article, in its Supporting Information, and openly available in NP-MRD under accession numbers NP0351265–NP0351274 at https://np-mrd.org/.

## Notes

Detailed description of materials and methods, biological and ecological assays, mass spectrometry, NMR spectra, and computational XYZ coordinates (PDF)

## Abbreviations

HR-LCMS: high-resolution liquid chromatography mass spectrometry
LC: liquid chromatography
MS: mass spectrometry
NMR: nuclear magnetic resonance
σ_1_R: sigma-1 receptor
σ_2_R: sigma-2 receptor
DFT: density functional theory
ECD: electronic circular dichroism.
