# Supplementary material for: The Discovery of Complex Heterocycles from Millipede Secretions
Source: J Am Chem Soc. 2025 Jul 17;147(30):26813–9. doi: 10.1021/jacs.5c08079 (PMC12314897; doi:10.1021/jacs.5c08079)
Supplement: Supplementary file 1 [file ja5c08079_si_001.pdf]

Supporting Information

The Discovery of Complex Heterocycles from Millipede Secretions

Paige Banks,[a] Carla Menegatti,[a] Lin Du,[b] Paul E. Marek,[c] Emily Mevers[a],*

[a]Department of Chemistry, Virginia Tech, Blacksburg, VA, 24061, USA; [b]Molecular Targets Program, Center for Cancer Research, National Cancer Institute, Frederick, MD, 24701, USA; [c]Department of Entomology, Virginia Tech, Blacksburg, VA, 24061, USA

*Email: emevers@vt.edu; Phone: (540)-231-6570

1. DATA DEPOSITION 4
NMR 4
2. EXPERIMENTAL METHODS 4
GENERAL EXPERIMENTAL PROCEDURES. 4
TABLE S1. *ANDROGNATHUS CORTICARIUS* COLLECTIONS 4
EXTRACTION AND PURIFICATION OF THE ANDROGNATHINES AND ANDROGNATHANOLS 4
HYDROGENATION AND HYDROLYSIS OF CRUDE MILLIPEDE EXTRACT. 7
ACETONIDE PROTECTION OF ANDROGNATHINE CORE (19) 8
ANDROGNATHINE BENZOATE (20) FOR ECD. 8
GCMS ANALYSIS OF FATTY ACID COMPOSITION IN *A. CORTICARIUS* CRUDE EXTRACT. 8
GCMS ANALYSIS OF FATTY ACID COMPOSITION IN ANDROGNATHANOL A (13) AND B (14). 8
DFT CALCULATION OF ECD SPECTRA FOR ANDROGNATHANOLS. 8
LCMS QUANTIFICATION OF ANDROGNATHANOLS AND ANDROGNATHINES. 9
ANT ASSAY. 9
SIGMA-1 AND SIGMA-2 RECEPTOR ASSAY PROCEDURE FROM PSYCHOACTIVE DRUG SCREENING PROGRAM (PDSP). 9
FIGURE S1. ^{1}H NMR SPECTRA FOR ANDROGNATHINE A (**1**) 10
FIGURE S2. GHSQC NMR SPECTRA FOR ANDROGNATHINE A (**1**) 11
FIGURE S3. H2BC NMR SPECTRA FOR ANDROGNATHINE A (**1**) 11
FIGURE S4. HMBC NMR SPECTRA FOR ANDROGNATHINE A (**1**) 12
FIGURE S5. EASYROESY NMR SPECTRA FOR ANDROGNATHINE A (**1**) 12
FIGURE S6. DQFCOSY NMR SPECTRA FOR ANDROGNATHINE A (**1**) 13
FIGURE S7. ^{1}H NMR SPECTRA FOR ANDROGNATHINE B (**2**) 13
FIGURE S8. GHSQC NMR SPECTRA FOR ANDROGNATHINE B (**2**) 14
FIGURE S9. H2BC NMR SPECTRA FOR ANDROGNATHINE B (**2**) 14
FIGURE S10. HMBC NMR SPECTRA FOR ANDROGNATHINE B (**2**) 15
FIGURE S11. EASYROESY NMR SPECTRA FOR ANDROGNATHINE B (**2**) 15
FIGURE S12. DQFCOSY NMR SPECTRA FOR ANDROGNATHINE B (**2**) 16
FIGURE S13. ^{1}H NMR SPECTRA FOR ANDROGNATHINE C (**3**) 16
FIGURE S14. GHSQC NMR SPECTRA FOR ANDROGNATHINE C (**3**) 17
FIGURE S15. H2BC NMR SPECTRA FOR ANDROGNATHINE C (**3**) 17
FIGURE S16. HMBC NMR SPECTRA FOR ANDROGNATHINE C (**3**) 18
FIGURE S17. EASYROESY NMR SPECTRA FOR ANDROGNATHINE C (**3**) 18
FIGURE S18. DQFCOSY NMR SPECTRA FOR ANDROGNATHINE C (**3**) 19
FIGURE S19. ^{1}H NMR SPECTRA FOR ANDROGNATHINE D (**4**) 19
FIGURE S20. GHSQC NMR SPECTRA FOR ANDROGNATHINE D (**4**) 20
FIGURE S21. H2BC NMR SPECTRA FOR ANDROGNATHINE D (**4**) 20
FIGURE S22. HMBC NMR SPECTRA FOR ANDROGNATHINE D (**4**) 21
FIGURE S23. COSY NMR SPECTRA FOR ANDROGNATHINE D (**4**) 21
FIGURE S24. ^{1}H NMR SPECTRA FOR ANDROGNATHINE E (**5**) 21
FIGURE S25. GHSQC NMR SPECTRA FOR ANDROGNATHINE E (**5**) 22
FIGURE S26. H2BC NMR SPECTRA FOR ANDROGNATHINE E (**5**) 22
FIGURE S27. HMBC NMR SPECTRA FOR ANDROGNATHINE E (**5**) 23
FIGURE S28. COSY NMR SPECTRA FOR ANDROGNATHINE E (**5**) 23
FIGURE S29. ^{1}H NMR SPECTRA FOR ANDROGNATHINE F (**6**) 24
FIGURE S30. GHSQC NMR SPECTRA FOR ANDROGNATHINE F (**6**) 24
FIGURE S31. HMBC NMR SPECTRA FOR ANDROGNATHINE F (**6**) 25
FIGURE S32. ^{1}H NMR SPECTRA FOR ANDROGNATHINE ACETONIDE (**19**) 25
FIGURE S33. GHSQC NMR SPECTRA FOR ANDROGNATHINE ACETONIDE (**19**) 26
FIGURE S34. DQFCOSY NMR SPECTRA FOR ANDROGNATHINE ACETONIDE (**19**) 26
FIGURE S35. HMBC NMR SPECTRA FOR ANDROGNATHINE ACETONIDE (**19**) 27
FIGURE S36. EASYROESY NMR SPECTRA FOR ANDROGNATHINE ACETONIDE (**19**) 27
FIGURE S37. 1D NOE SPECTRUM OF ANDROGNATHINE ACETONIDE (**19**) 28
FIGURE S38. NEWMAN PROJECTIONS FOR THE CONFORMERS FOR THE TWO *ANTI-*CONFIGURATIONS 29
FIGURE S39. ^{1}H NMR SPECTRA FOR ANDROGNATHANOL A (**13**) 30

FIGURE S40. GHSQC NMR SPECTRA FOR ANDROGNATHANOL A (**13**) 30
FIGURE S41. H2BC NMR SPECTRA FOR ANDROGNATHANOL A (**13**) 31
FIGURE S42. HMBC NMR SPECTRA FOR ANDROGNATHANOL A (**13**)..31
FIGURE S43. EASYROESY NMR SPECTRA FOR ANDROGNATHANOL A (**13**) 32
FIGURE S44. DQFCOSY NMR SPECTRA FOR ANDROGNATHANOL A (**13**) 32
FIGURE S45. ^{1}H NMR SPECTRA FOR ANDROGNATHANOL B (**14**)..33
FIGURE S46. GHSQC NMR SPECTRA FOR ANDROGNATHANOL B (**14**) 33
FIGURE S47. H2BC NMR SPECTRA FOR ANDROGNATHANOL B (**14**)..34
FIGURE S48. HMBC NMR SPECTRA FOR ANDROGNATHANOL B (**14**) 34
FIGURE S49. EASYROESY NMR SPECTRA FOR ANDROGNATHANOL B (**14**) 35
FIGURE S50. DQFCOSY NMR SPECTRA FOR ANDROGNATHANOL B (**14**) 35
FIGURE S51. ^{1}H NMR SPECTRA FOR ANDROGNATHANOL C (**15**)..36
FIGURE S52. GHSQC NMR SPECTRA FOR ANDROGNATHANOL C (**15**) 36
FIGURE S53. H2BC NMR SPECTRA FOR ANDROGNATHANOL C (**15**)..37
FIGURE S54. HMBC NMR SPECTRA FOR ANDROGNATHANOL C (**15**) 37
FIGURE S55. EASYROESY NMR SPECTRA FOR ANDROGNATHANOL C (**15**) 38
FIGURE S56. DQFCOSY NMR SPECTRA FOR ANDROGNATHANOL C (**15**) 38
FIGURE S57. ^{1}H NMR SPECTRA FOR ANDROGNATHANOL D (**16**) 39
FIGURE S58. GHSQC NMR SPECTRA FOR ANDROGNATHANOL D (**16**) 39
FIGURE S59. H2BC NMR SPECTRA FOR ANDROGNATHANOL D (**16**) 40
FIGURE S60. HMBC NMR SPECTRA FOR ANDROGNATHANOL D (**16**)..40
FIGURE S61. ^{1}H NMR SPECTRA FOR ANDROGNATHANOL HYDRATE (**21**) 41
FIGURE S62. GHSQC NMR SPECTRA FOR ANDROGNATHANOL HYDRATE (**21**)....................................41
FIGURE S63. GCOSY SPECTRA FOR ANDROGNATHANOL HYDRATE (**21**) 42
FIGURE S64. HMBC SPECTRA FOR ANDROGNATHANOL HYDRATE (**21**)..42
FIGURE S65. 2D EASYROESY SPECTRA FOR ANDROGNATHANOL HYDRATE (**21**) 43
FIGURE S66. 1D NOE SPECTRA FOR ANDROGNATHANOL HYDRATE (**21**)...43
FIGURE S67. ESTER SIDE CHAIN REACTIONS 44
FIGURE S68. ABSOLUTE QUANTIFICATION OF ANDROGNATHINE A (**1**)..44
FIGURE S69. ABSOLUTE QUANTIFICATION OF ANDROGNATHANOL A (**13**) AND B (**14**) 45
FIGURE S70. ALKALOIDS COMPARED TO LENGTH AND NUMBER OF OZOPORES 46
FIGURE S71. LR-LCMS ANALYSIS OF *A. CORTICARIUS* SAMPLES COLLECTED IN DISTINCT GEOGRAPHICAL LOCATIONS..47
FIGURE S72. Σ_1 RECEPTOR BINDING. SECONDARY SCREENING OF ANDROGNATHINE A (**1**), AND POSITIVE CONTROL HALOPERIDOL. 47
FIGURE S73. Σ_2 RECEPTOR BINDING 48
TABLE S2. NMR SPECTROSCOPY DATA FOR ANDROGNATHINE A (**1**) 49
TABLE S3. NMR SPECTROSCOPY DATA FOR ANDROGNATHINE B (**2**) 50
TABLE S4. NMR SPECTROSCOPY DATA FOR ANDROGNATHINE C (**3**) 51
TABLE S5. NMR SPECTROSCOPY DATA FOR ANDROGNATHINE D (**4**)..52
TABLE S6. NMR SPECTROSCOPY DATA FOR ANDROGNATHINE E (**5**) 53
TABLE S7. NMR SPECTROSCOPY DATA FOR ANDROGNATHINE F (**6**)...54
TABLE S8. NMR SPECTROSCOPY DATA FOR ANDROGNATHINE ACETONIDE (**19**) 55
TABLE S9. NMR SPECTROSCOPY DATA FOR ANDROGNATHANOL A (**13**)..56
TABLE S10. NMR SPECTROSCOPY DATA FOR ANDROGNATHANOL B (**14**) 57
TABLE S11. NMR SPECTROSCOPY DATA FOR ANDROGNATHANOL C (**15**) 58
TABLE S12. NMR SPECTROSCOPY DATA FOR ANDROGNATHANOL D (**16**)..59
TABLE S13. NMR SPECTROSCOPY DATA FOR ANDROGNATHANOL HYDRATE (**21**)..............................60
TABLE S14. QUANTIFICATION OF ALKALOIDS PRODUCED BY *A. CORTICARIUS* 61
TABLE S15. Σ_1 RECEPTOR BINDING 62
TABLE S16. Σ_2 RECEPTOR BINDING 62
TABLE S17. PDSP PRIMARY SCREEN AT 10 MM 62
TABLE S18. NAV1.5 HUMAN SODIUM ION CHANNEL CELL BASED APC LEADHUNTER ASSAY.........63
TABLE S19. NAV1.8 HUMAN SODIUM ION CHANNEL CELL BASED APC LEADHUNTER ASSAY.........64
TABLE S20. XYZ COORDINATES ANDROGNATHANOLS...64

1.Data Deposition

NMR Raw Data Files. Openly available in NP-MRD under accession numbers NP0341265 – NP0351274 at https://np-mrd.org/.

2.Experimental Methods

General Experimental Procedures. NMR spectra were recorded with d_6-DMSO with the residual solvent peak as an internal standard (δ_C 39.5, δ_H 2.50) on Bruker Advance III 600 MHz instrument equipped with a triple resonance inverse (CP-TCI) Prodigy N2 cooled CryoProbe (600 and 150 MHz for ^{1}H and ^{13}C NMR, respectively). LR-LCMS data was obtained using an Agilent 1200 series HPLC system equipped with a photo-diode array detector and a Thermo LTQ mass spectrometer. HR-ESI-MS was carried out using a Shimadzu LC-q-TOF Mass Spectrometer equipped with an HPLC system. HPLC purifications were carried out using Agilent 1200 series or 1260 Infinity II HPLC systems (Agilent Technologies) equipped with a photodiode array detector. All solvents were of HPLC quality. Optical rotations were measured on a JASCO P-2000 polarimeter

Millipede Collections. The millipedes were collected from the underside of decaying logs underneath trees, often feeding on fungus. They were preserved in methanol, which was then removed, replaced, and dried down for a crude extract. The locations collected are shown in **Table S1**.

Table S1. *Andrognathus corticarius* Collections

Millipede	Location	Coordinates	Elevation	Date
A. corticarius	Virginia, Montgomery Co., Blacksburg, Virginia Tech, Stadium Woods	37.22124, -80.41586	Elev. 647 m	29 June 2022
A. corticarius	Virginia, Montgomery Co., Caldwell Fields Campground	37.33553, -80.32430	Elev. 521 m	25 April 2023
A. corticarius	Virginia, Montgomery Co., Blacksburg, Virginia Tech, Stadium Woods	37.22124, -80.41586	Elev. 647 m	18 May 2023
A. corticarius	Virginia, Montgomery Co., Blacksburg, Virginia Tech, Stadium Woods	37.22124, -80.41586	Elev. 647 m	18 May 2023
A. corticarius	Virginia, Montgomery Co., Pandapas Pond	37.28257, -80.46991	Elev. 672 m	19 July 2023
A. corticarius (single millipedes)	Virginia, Montgomery Co., Blacksburg, Virginia Tech, Stadium Woods	37.22124, -80.41586	Elev. 647 m	09 August 2023
A. corticarius (single millipedes)	Virginia, Montgomery Co., Pandapas Pond	37.28257, -80.46991	Elev. 672 m	09 August 2023
A. corticarius	Talladega County, Alabama, Sylaward Trail at Lake Howard	33.19511, -86.19370	Elev. 187 m	24 September 2023
A. corticarius	Swain County, North Carolina, Findley Falls Trailhead	35.28291, -83.67422	Elev. 621 m	06 July 2023

Extraction and Purification of the Andrognathines and Andrognathanols from *A. corticarius*. The millipedes were extracted using MeOH, and the combined organic material was dried down under vacuum. The extract was then resuspended in 1:1 MeCN/H_2O and separated using reverse-phase high-performance liquid chromatography (RP-HPLC) equipped with a Phenomenex 4 µm Hydro semi-preparative column (250 × 10 mm) under the following conditions. Holding 20% MeCN + 0.1% formic acid (FA)/80% H_2O + 0.1% FA for 5 min, then a linear gradient to 60% MeCN + 0.1% FA/40% H_2O + 0.1% FA over 45 min at a flow rate of 3 mL/min to yield pure andrognathines and andrognathanols.

*Andrognathine A (**1**):* amorphous solid; $[\alpha]^{23}_D$ +23.6 (*c* 0.015, MeOH); ^{1}H NMR (600 MHz, d_6-DMSO) δ 5.45 (d, *J* = 9.3, CH_2, H-11), 5.38 (d, *J* = 9.3, CH, H-10), 5.10 (s, CH_2, H-14b), 5.04 (s, CH_2, H-14a), 2.94 (m, CH_2, H-5b), 2.91 (m, CH_2, H-4b), 2.27 (m, CH_2, H-21b), 2.25 (m, CH_2, H-16b), 2.22 (m, CH_2, H-21a), 2.21 (m, CH_2, H-16a), 2.03 (m, CH_2, H-7b), 1.96 (m, CH_2, H-5a), 1.85 (m, CH_2, H-4a), 1.76 (m, *J* = 10.2, CH, H-8), 1.70 (s, CH_3, H-13), 1.65 (m, CH_2, H-6), 1.58 (m, CH_2, H-3b), 1.53 (m, CH_2, H-7a), 1.53 (m, CH_2, H-22), 1.45 (m, CH_2, H-17), 1.24 (m, CH_2, H-18), 1.21 (m, CH, H-2), 1.21 (m, CH_2, H-3a), 1.16 (m, CH, H-9), 1.00 (d, *J* =5.2, CH_3, H-1), 0.87 (m, CH_3, H-19), 0.86 (m, CH_3, H-23); ^{13}C NMR (d_6-DMSO; through HSQC and HMBC) δ 172.3 (C-15), 171.8 (C-20), 139.9 (C-12), 118.1 (C-14), 76.1 (C-11), 70.1 (C-10), 64.3 (C-8), 53.1 (C-5), 51.8 (C-4), 47.1 (C-9), 35.3 (C-21), 34.1 (C-3), 33.2

(C-16), 32.5 (C-2), 29.7 (C-7), 26.5 (C-17), 21.9 (C-23), 21.6 (C-18), 20.5 (C-6), 19.8 (C-1), 17.9 (C-22), 17.5 (C-13), 13.3 (C-19); ESI MS/MS (q-TOF) *m/z* 394.2954 ($C_{23}H_{39}NO_4$), 306.2429 ($C_{19}H_{31}NO_2$), 292.2273 ($C_{18}H_{29}NO_2$), 222.1857 ($C_{14}H_{23}NO$), 204.1749 ($C_{14}H_{21}N$), 138.1280 ($C_9H_{15}N$); HRESIMS $[M+H]^+$ *m/z* 394.2954 (calcd $C_{23}H_{39}NO_4$ $[M+H]^+$ 394.2952, Δ0.5).

*Andrognathine B (**2**):* amorphous solid; ^{1}H NMR (600 MHz, d_6-DMSO) δ 5.47 (d, *J* = 9.7, CH, H-11), 5.38 (d, *J* = 9.7, CH, H-10), 5.11 (s, CH_2, H-14b), 5.05 (s, CH_2, H-14a), 2.9 (m, CH_2, H-5b), 2.88 (m, CH_2, H-4b), 2.27 (m, CH_2, H-16b), 2.27 (m, CH_2, H-20b), 2.21 (m, CH_2, H-16a), 2.21 (m, CH_2, H-20a), 2.02 (m, CH_2, H-7b), 1.91 (m, CH_2, H-5a), 1.79 (m, CH_2, H-4a), 1.71 (s, CH_3, H-13), 1.68 (m, CH, H-8), 1.63 (m, CH_2, H-6), 1.58 (m, CH_2, H-3b), 1.51 (m, CH_2, H-7a), 1.51 (m, CH_2, H-17), 1.51 (m, CH_2, H-21), 1.20 (m, CH, H-2), 1.20 (m, CH_2, H-3a), 1.12 (m, CH, H-9), 1.01 (d, *J* = 5.2, CH_3, H-1), 0.87 (m, CH_3, H-18), 0.87 (m, CH_3, H-22); ^{13}C NMR (d_6-DMSO; through HSQC and HMBC) δ 171.8 (C-15), 171.8 (C-19), 140.0 (C-12), 118.1 (C-14), 75.9 (C-11), 70.1 (C-10), 64.1 (C-8), 53.2 (C-5), 51.8 (C-4), 47.1 (C-9), 35.2 (C-16), 35.2 (C-20), 34.2 (C-3), 32.5 (C-2), 29.7 (C-7), 20.4 (C-6), 19.7 (C-1), 17.7 (C-17), 17.7 (C-21), 17.5 (C-13), 13.2 (C-18), 13.2 (C-22); ESI MS/MS (q-TOF) *m/z* 380.2801 ($C_{22}H_{37}NO_4$), 292.2274 ($C_{18}H_{29}NO_2$), 222.1858 ($C_{14}H_{23}NO$), 204.1752 ($C_{14}H_{21}N$), 138.1282 ($C_9H_{15}N$); HRESIMS $[M+H]^+$ *m/z* 380.2801 (calcd $C_{22}H_{37}NO_4$ $[M+H]^+$ 380.2795, Δ1.5).

*Andrognathine C (**3**):* amorphous solid; $[\alpha]^{23}_D$ +4.67 (*c* 0.015, MeOH); ^{1}H NMR (600 MHz, d_6-DMSO) 5.47 (d, *J* = 8.4, CH, H-11), 5.37 (d, *J* = 8.4, CH, H-10), 5.10 (s, CH_2, H-14b), 5.04 (s, CH_2, H-14a), 2.90 (m, CH_2, H-5b), 2.87 (m, CH_2, H-4b), 2.30 (m, CH_2, H-16b), 2.26 (m, CH_2, H-16a), 2.26 (m, CH_2, H-20), 2.02 (m, CH_2, H-7b), 1.90 (m, CH_2, H-5a), 1.79 (m, CH_2, H-4a), 1.71 (s, CH_3, H-13), 1.68 (m, CH, H-8), 1.64 (m, CH_2, H-6), 1.57 (m, CH_2, H-3b), 1.51 (m, CH_2, H-7a), 1.51 (m, CH_2, H-22), 1.45 (m, CH_2, H-21), 1.20 (m, CH, H-2), 1.20 (m, CH_2, H-3a), 1.12 (t, *J* = 9.3, CH, H-9), 1.02 (m, CH_3, H-17), 1.00 (m, CH_3, H-1). ^{13}C NMR (d_6-DMSO; through HSQC and HMBC) δ 173.2 (C-15), 171.9 (C-19), 140.0 (C-12), 118.1 (C-14), 75.9 (C-11), 70.1 (C-10), 64.1 (C-8), 53.2 (C-5), 51.7 (C-4), 47.1 (C-9), 34.1 (C-3), 33.1 (C-20), 32.4 (C-2), 29.6 (C-7), 26.7 (C-16), 26.3 (C-21), 20.4 (C-6), 19.6 (C-1), 17.7 (C-22), 17.5 (C-13), 13.3 (C-23), 8.8 (C-17); ESI MS/MS (q-TOF) *m/z* 380.2806 ($C_{22}H_{37}NO_4$), 306.2435 ($C_{19}H_{31}NO_2$), 278.2126 ($C_{17}H_{27}NO_2$), 222.1859 ($C_{14}H_{23}NO$), 204.1756 ($C_{14}H_{21}N$), 138.1284 ($C_9H_{15}N$); HRESIMS $[M+H]^+$ *m/z* 380.2806 (calcd $C_{22}H_{37}NO_4$ $[M+H]^+$ 380.2795, Δ2.8).

*Andrognathine D (**4**):* amorphous solid; ^{1}H NMR (600 MHz, d_6-DMSO) δ 5.46 (d, *J* = 9.0, CH, H-11), 5.37 (d, *J* = 9.0, CH, H-10), 5.10 (s, CH_2, H-14b), 5.04 (s, CH_2, H-14a), 2.90 (m, CH_2, H-5a), 2.87 (m, CH_2, H-4a), 2.27 (m, CH_2, H-20b), 2.26 (m, CH_2, H-16b), 2.23 (m, CH_2, H-16a), 2.22 (m, CH_2, H-20a), 1.92 (m, CH_2, H-5b), 1.80 (m, CH_2, H-4b), 1.71 (s, CH_3, H-13), 1.67 (m, CH, H-8), 1.63 (m, CH_2, H-6), 1.57 (m, CH_2, H-3), 1.52 (m, CH_2, H-7), 1.52 (q, *J* = 7.1, CH_2, H-17), 1.46 (t, *J* = 7.7, CH_2, H-21), 1.27 (m, CH_2, H-22), 1.20 (m, CH, H-2), 1.13 (m, CH, H-9), 1.01 (m, CH_3, H-1), 0.87 (m, CH_3, H-18), 0.85 (m, CH_3, H-23); ^{13}C NMR (d_6-DMSO; through HSQC and HMBC) δ 172.2 (C-15), 171.8 (C-19), 140.4 (C-12), 117.9 (C-14), 75.9 (C-11), 70.3 (C-10), 64.1 (C-8), 53.1 (C-5), 51.8 (C-4), 47.1 (C-9), 35.4 (C-16), 34.3 (C-3), 33.2 (C-20), 32.5 (C-2), 26.3 (C-7), 26.2 (C-21), 21.7 (C-18), 21.2 (C-22), 20.5 (C-6), 19.6 (C-1), 17.6 (C-13), 17.5 (C-17), 13.1 (C-23); ESI MS/MS (q-TOF) *m/z* 394.2961 ($C_{23}H_{39}NO_4$), 306.2437 ($C_{19}H_{31}NO_2$), 292.2283 ($C_{18}H_{29}NO_2$), 222.1861 ($C_{14}H_{23}NO$), 204.1755 ($C_{14}H_{21}N$), 138.1283 ($C_9H_{15}N$); HRESIMS $[M+H]^+$ *m/z* 394.2961 (calcd $C_{23}H_{39}NO_4$ $[M+H]^+$ 394.2952, Δ2.2).

*Andrognathine E (**5**):* amorphous solid; ^{1}H NMR (600 MHz, d_6-DMSO) δ 5.46 (d, *J* = 9.2, CH, H-11), 5.38 (d, *J* = 9.2, CH, H-10), 5.10 (s, CH_2, H-14b), 5.04 (s, CH_2, H-14a), 2.90 (m, CH_2, H-5b), 2.87 (m, CH_2, H-4b), 2.27 (m, CH_2, H-16a), 2.22 (m, CH_2, H-16b), 2.15 (m, CH_2, H-20b), 2.11 (m, CH_2, H-20a), 1.90 (m, CH_2, H-5a), 1.79 (t, *J* = 10.9, CH_2, H-4a), 1.70 (s, CH_3, H-13), 1.68 (m, CH, H-8), 1.62 (m, CH_2, H-6), 1.57 (d, *J* = 9.1, CH_2, H-3b), 1.52 (m, CH_2, H-7), 1.52 (m, CH_2, H-17), 1.21 (m, CH_2, H-3a), 1.20 (m, CH, H-2), 1.13 (m, CH, H-9), 1.01 (d, *J* = 5.4, CH_3, H-1), 0.88 (m, CH_3, H-18), 0.88 (m, CH_3, H-21); ^{13}C NMR (d_6-DMSO; through HSQC and HMBC) δ 172.2 (C-19), 171.2 (C-15), 139.9 (C-12), 118.1 (C-14), 76.1 (C-11), 70.3 (C-10), 64.3 (C-8), 53.3 (C-5), 51.9 (C-4), 47.3 (C-9), 42.6 (C-20), 35.4 (C-16), 34.3 (C-3), 32.6 (C-2), 21.5 (C-21), 20.5 (C-6), 19.8 (C-1), 17.9 (C-7), 17.6 (C-13), 17.6 (C-17), 13.5 (C-18); ESI MS/MS (q-TOF) *m/z* 366.2644 ($C_{21}H_{35}NO_4$), 292.2279 ($C_{18}H_{29}NO_2$), 278.2119 ($C_{17}H_{27}NO_2$), 222.1858 ($C_{14}H_{23}NO$), 204.1752 ($C_{14}H_{21}N$), 138.1283 ($C_9H_{15}N$); HRESIMS $[M+H]^+$ *m/z* 366.2644 (calcd $C_{21}H_{35}NO_4$ $[M+H]^+$ 366.2639, Δ1.3).

*Andrognathine F (**6**):* amorphous solid; ^{1}H NMR (600 MHz, d_6-DMSO) δ 5.47 (m, CH, H-11), 5.36 (m, CH, H-10), 5.11 (s, CH_2, H-14b), 5.05 (s, CH_2, H-14a), 2.90 (m, CH_2, H-5b), 2.87 (m, CH_2, H-4b), 2.27 (m, CH_2, H-16), 2.27 (m, CH_2, H-19b), 2.20 (m, CH_2, H-19a), 2.02 (m, CH_2, H-7b), 1.91 (m, CH_2, H-5a), 1.80 (m, CH_2, H-4a), 1.70 (s, CH_3,

H-13), 1.68 (m, CH, H-8), 1.63 (m, CH_2, H-6), 1.57 (m, CH_2, H-3b), 1.50 (m, CH_2, H-7a), 1.50 (m, CH_2, H-20), 1.20 (m, CH, H-2), 1.20 (m, CH_2, H-3a), 1.12 (m, CH, H-9), 1.01 (m, CH_3, H-1), 1.01 (m, CH_3, H-17), 0.85 (m, CH_3, H-21); ^{13}C NMR (d_6-DMSO; through HSQC and HMBC) δ 173.1 (C-15), 171.7 (C-18), 139.9 (C-12), 118.1 (C-14), 76.1 (C-11), 70.3 (C-10), 64.1 (C-8), 53.2 (C-5), 52.0 (C-4), 47.3 (C-9), 35.3 (C-19), 34.4 (C-3), 32.5 (C-2), 29.9 (C-7), 26.9 (C-16), 20.5 (C-6), 19.8 (C-1), 17.9 (C-20), 17.7 (C-13), 13.5 (C-21), 9.1 (C-17); ESI MS/MS (q-TOF) *m/z* 366.2654 ($C_{21}H_{35}NO_4$), 292.2280 ($C_{18}H_{29}NO_2$), 278.2115 ($C_{17}H_{27}NO_2$), 222.1854 ($C_{14}H_{23}NO$), 204.1746 ($C_{14}H_{21}N$), 138.1283 ($C_9H_{15}N$); HRESIMS $[M+H]^+$ *m/z* 366.2654 (calcd $C_{21}H_{35}NO_4$ $[M+H]^+$ 366.2639, Δ4.3).

Andrognathine G (7): ESI MS/MS (q-TOF) *m/z* 240.1969 ($C_{14}H_{25}NO_2$), 184.1331 ($C_{10}H_{17}NO_2$), 168.1387 ($C_{10}H_{17}NO$); HRESIMS $[M+H]^+$ *m/z* 240.1969 (calcd $C_{14}H_{25}NO_2$ $[M+H]^+$ 240.1958, Δ 4.4).

*Andrognathine H (**8**):* ESI MS/MS (q-TOF) *m/z* 352.2473 ($C_{20}H_{33}NO_4$), 292.2263 ($C_{18}H_{29}NO_2$), 264.1967 ($C_{16}H_{25}NO_2$), 222.1866 ($C_{14}H_{23}NO$), 204.1749 ($C_{14}H_{21}N$); HRESIMS $[M+H]^+$ *m/z* 352.2473 (calcd $C_{20}H_{33}NO_4$ $[M+H]^+$ 352.2482, Δ2.8).

*Andrognathine I (**9**):* ESI MS/MS (q-TOF) *m/z* 352.2486 ($C_{20}H_{33}NO_4$), 292.2263 ($C_{18}H_{29}NO_2$), 264.1976 ($C_{16}H_{25}NO_2$), 222.1861 ($C_{14}H_{23}NO$), 204.1749 ($C_{14}H_{21}N$); HRESIMS $[M+H]^+$ *m/z* 352.2486 (calcd $C_{20}H_{33}NO_4$ $[M+H]^+$ 352.2482, Δ1.0).

*Andrognathaine J (**10**):* ESI MS/MS (q-TOF) *m/z* 380.2805 ($C_{22}H_{37}NO_4$), 306.2445 ($C_{19}H_{31}NO_2$), 278.2123 ($C_{17}H_{27}NO_2$), 222.1864 ($C_{14}H_{23}NO$), 204.1754 ($C_{14}H_{21}N$), 138.1284 ($C_9H_{15}N$); HRESIMS $[M+H]^+$ *m/z* 380.2805 (calcd $C_{22}H_{37}NO_4$ $[M+H]^+$ 380.2795, Δ2.4).

*Andrognathaine K (**11**):* ESI MS/MS (q-TOF) *m/z* 352.2490 ($C_{20}H_{33}NO_4$), 278.2103 ($C_{17}H_{27}NO_2$), 222.1852 ($C_{14}H_{23}NO$), 204.1752 ($C_{14}H_{21}N$); HRESIMS $[M+H]^+$ *m/z* 352.2490 (calcd $C_{20}H_{33}NO_4$ $[M+H]^+$ 352.2482, Δ2.1).

*Andrognathaine L (**12**):* ESI MS/MS (q-TOF) *m/z* 408.3114 ($C_{24}H_{41}NO_4$), 306.2429 ($C_{19}H_{31}NO_2$), 222.1858 ($C_{14}H_{23}NO$), 204.1752 ($C_{14}H_{21}N$); HRESIMS $[M+H]^+$ *m/z* 408.3114 (calcd $C_{24}H_{41}NO_4$ $[M+H]^+$ 408.3108, Δ1.3).

*Andrognathanol A (**13**):* amorphous solid; $[\alpha]^{23}_D$ +31.7 (*c* 0.015, MeOH); ^{1}H NMR (600 MHz, d_6-DMSO) δ 4.92 (s, CH, H-6), 4.60 (d, *J* = 4.4, CH, H-11), 4.01 (sext, *J* = 6.4, CH, H-21), 3.60 (d, *J* = 5.5, CH, H-1), 2.94 (d, *J* = 10.7, CH_2, H-3b), 2.55 (d, *J* = 12.0, CH_2, H-4b), 2.37 (m, CH_2, H-20), 2.29 (q, *J* = 7.3, CH_2, H-17), 2.15 (m, CH_2, H-3a), 2.13 (m, CH_2, H-4a), 2.01 (q, *J* = 4.3, CH, H-10), 1.90 (m, CH, H-8), 1.90 (m, CH_2, H-14b), 1.73 (m, CH_2, H-13b), 1.70 (m, CH, H-2), 1.66 (m, CH_2, H-5b), 1.53 (m, CH_2, H-7b), 1.49 (m, CH, H-9), 1.43 (m, CH_2, H-7a), 1.30 (pent, *J* = 6.7, CH_2, H-14a), 1.23 (m, CH_2, H-5a), 1.14 (dd, *J* = 8.6 and 12.5, CH_2, H-13a), 1.10 (d, *J* = 6.0, CH_3, H-22), 1.01 (t, *J* = 7.4, CH_3, H-18), 0.78 (s, CH_3, H-15); ^{13}C NMR (d_6-DMSO; through HSQC and HMBC) δ 172.9 (C-16), 171.1 (C-19), 78.0 (C-11), 75.4 (C-1), 67.7 (C-6), 63.2 (C-21), 57.6 (C-8), 55.4 (C-3), 49.5 (C-4), 44.1 (C-20), 43.4 (C-2), 42.6 (C-12), 40.8 (C-10), 32.0 (C-7), 30.6 (C-13), 30.3 (C-9), 28.8 (C-5), 26.9 (C-17), 23.2 (C-22), 19.4 (C-15), 16.9 (C-14), 8.8 (C-18); ESI MS/MS (q-TOF) *m/z* 410.2544 ($C_{22}H_{35}NO_6$), 366.2283 ($C_{20}H_{31}NO_5$), 336.2180 ($C_{19}H_{29}NO_4$), 306.2750 ($C_{18}H_{27}NO_4$), 292.1910 ($C_{17}H_{25}NO_3$), 250.1809 ($C_{14}H_{23}NO_2$), 232.1711 ($C_{15}H_{21}NO$); HRESIMS $[M+H]^+$ *m/z* 410.2544 (calcd $C_{22}H_{35}NO_6$ $[M+H]^+$ 410.2537, Δ1.6).

*Andrognathanol B (**14**):* amorphous solid; $[\alpha]^{23}_D$ +19.2 (*c* 0.015, MeOH); ^{1}H NMR (600 MHz, d_6-DMSO) δ 4.91 (s, CH, H-6), 4.60 (d, *J* = 4.6, CH, H-11), 3.76 (m, CH, H-21), 3.60 (d, *J* = 5.7, CH, H-1), 2.94 (d, *J* = 12.4, CH_2, H-3b), 2.56 (m, CH_2, H-4b), 2.41 (d, *J* = 5.6, CH_2, H-20b), 2.31 (m, CH_2, H-20a), 2.28 (m, CH_2, H-17), 2.16 (m, CH_2, H-3a), 2.14 (m, CH_2, H-4a), 2.02 (m, CH, H-10), 1.91 (m, CH, H-8), 1.89 (m, CH_2, H-14b), 1.72 (m, CH_2, H-13b), 1.70 (m, CH, H-2), 1.66 (m, CH_2, H-5), 1.54 (m, CH_2, H-7b), 1.50 (m, CH, H-9), 1.43 (m, CH_2, H-7a), 1.36 (m, CH_2, H-22), 1.29 (m, CH_2, H-14a), 1.14 (m, CH_2, H-13a), 1.01 (t, *J* = 7.4, CH_3, H-18), 0.85 (t, *J* = 7.4, CH_3, H-23), 0.78 (s, CH_3, H-15); ^{13}C NMR (d_6-DMSO; through HSQC and HMBC) δ 172.9 (C-16), 171.3 (C-19), 78.1 (C-11), 75.5 (C-1), 68.3 (C-21), 67.4 (C-6), 57.7 (C-8), 55.4 (C-3), 49.6 (C-4), 43.5 (C-2), 42.4 (C-12), 42.1 (C-20), 40.8 (C-10), 32.1 (C-7), 30.6 (C-13), 30.3 (C-9), 29.5 (C-22), 28.8 (C-5), 26.9 (C-17), 19.4 (C-15), 16.9 (C-14), 9.6 (C-23), 8.8 (C-18); ESI MS/MS (q-TOF) *m/z* 424.2698 ($C_{23}H_{37}NO_6$), 366.2278 ($C_{20}H_{31}NO_5$), 350.2331 ($C_{20}H_{31}NO_4$), 324.2176 ($C_{18}H_{29}NO_4$), 306.2071 ($C_{18}H_{27}NO_4$), 292.1910 ($C_{17}H_{25}NO_3$), 250.1805 ($C_{14}H_{23}NO_2$), 232.1700 ($C_{15}H_{21}NO$); HRESIMS $[M+H]^+$ *m/z* 424.3055 (calcd $C_{23}H_{37}NO_6$ $[M+H]^+$ 424.2694, Δ0.8).

*Andrognathanol C (**15**):* amorphous solid; $[\alpha]^{23}_{D}$ +19.2 (*c* 0.015, MeOH); ^{1}H NMR (600 MHz, d_6-DMSO) δ 4.91 (s, CH, H-6), 4.63 (d, *J* = 4.7, CH, H-11), 3.60 (d, *J* = 5.9, CH, H-1), 2.92 (d, *J* = 12.1, CH_2, H-3b), 2.54 (m, CH_2, H-4b), 2.29 (q, *J* = 7.5, CH_2, H-21), 2.21 (m, CH_2, H-17), 2.15 (m, CH_2, H-3a), 2.13 (m, CH_2, H-4a), 2.02 (m, CH, H-10), 1.97 (m, CH, H-18), 1.91 (m, CH_2, H-14b), 1.90 (m, CH, H-8), 1.71 (m, CH_2, H-13b), 1.70 (m, CH, H-2), 1.67 (m, CH_2, H-5b), 1.54 (m, CH_2, H-7b), 1.46 (m, CH, H-9), 1.43 (m, CH, H-7a), 1.30 (m, CH_2, H-14a), 1.23 (m, CH_2, H-5a), 1.15 (m, CH_2, H-13a), 1.01 (t, *J* = 7.5, CH_3, H-22), 0.91 (d, *J* = 6.6, CH_3, H-19), 0.78 (s, CH_3, H-15); ^{13}C NMR (d_6-DMSO; through HSQC and HMBC) δ 172.9 (C-20), 172.1 (C-16), 78.1 (C-11), 75.5 (C-1), 67.4 (C-6), 57.6 (C-8), 55.4 (C-3), 49.5 (C-4), 43.6 (C-2), 42.7 (C-17), 42.4 (C-12), 40.9 (C-10), 32.1 (C-7), 30.6 (C-13), 30.5 (C-9), 28.7 (C-5), 26.9 (C-21), 25.1 (C-18), 21.9 (C-19), 19.5 (C-15), 16.9 (C-14), 8.8 (C-22); ESI MS/MS (q-TOF) *m/z* 408.2746 ($C_{23}H_{37}NO_5$), 334.2377 ($C_{20}H_{31}NO_3$), 306.2063 ($C_{18}H_{27}NO_4$), 250.1792 ($C_{14}H_{23}NO_2$), 232.1687 ($C_{15}H_{21}NO$); HRESIMS $[M+H]^+$ *m/z* 408.2746 (calcd $C_{23}H_{37}NO_5$ $[M+H]^+$ 408.2745, Δ0.4).

*Andrognathanol D (**16**):* amorphous solid; $[\alpha]^{23}_{D}$ +19.2 (*c* 0.015, MeOH); ^{1}H NMR (600 MHz, d_6-DMSO) NMR (600 MHz, d_6-DMSO) δ 4.91 (s, CH, H-6), 4.62 (d, *J* = 4.6, CH, H-11), 3.60 (d, *J* = 6.1, CH, H-1), 2.92 (d, *J* = 11.7, CH_2, H-3b), 2.55 (m, CH_2, H-4b), 2.29 (q, *J* = 7.7, CH_2, H-17), 2.21 (dd, *J* = 4.5 and 2.4, CH_2, H-20), 2.16 (m, CH_2, H-3a), 2.13 (m, CH_2, H-4a), 2.01 (m, CH, H-10), 1.99 (m, CH, H-21), 1.91 (m, CH, H-8), 1.91 (m, CH_2, H-14b), 1.70 (m, CH, H-2), 1.70 (m, CH_2, H-13b), 1.66 (m, CH_2, H-5b), 1.52 (m, CH_2, H-7b), 1.45 (m, CH, H-9), 1.43 (m, CH_2, H-7a), 1.30 (m, CH_2, H-14a), 1.22 (m, CH_2, H-5a), 1.15 (m, CH_2, H-13a), 1.01 (t, *J* = 7.7, CH, H-18), 0.90 (d, *J* = 6.8, CH_3, H-22), 0.78 (s, CH_3, H-15); ^{13}C NMR (d_6-DMSO; through HSQC and HMBC) δ 172.9 (C-16), 172.2 (C-19), 78.2 (C-11), 75.6 (C-1), 67.5 (C-6), 57.8 (C-8), 55.6 (C-3), 49.6 (C-4), 43.6 (C-2), 42.9 (C-20), 42.4 (C-12), 41.0 (C-10), 32.2 (C-7), 30.7 (C-13), 30.7 (C-9), 28.8 (C-5), 27.0 (C-17), 25.2 (C-21), 22.0 (C-22), 19.6 (C-15), 17.0 (C-14), 9.0 (C-18); ESI MS/MS (q-TOF) *m/z* 408.2748 ($C_{23}H_{37}NO_5$), 334.2382 ($C_{20}H_{31}NO_3$), 306.2044 ($C_{18}H_{27}NO_4$), 250.1792 ($C_{14}H_{23}NO_2$), 232.1691 ($C_{15}H_{21}NO$); HRESIMS $[M+H]^+$ *m/z* 408.2747 (calcd $C_{23}H_{37}NO_5$ $[M+H]^+$ 408.2745, Δ0.5).

*Andrognathanol E (**17**):* ESI MS/MS (q-TOF) *m/z* 410.2553 ($C_{22}H_{35}NO_6$), 366.2291 ($C_{20}H_{31}NO_5$), 336.2178 ($C_{19}H_{29}NO_4$), 306.2050 ($C_{18}H_{27}NO_4$), 292.1918 ($C_{17}H_{25}NO_3$), 250.1799 ($C_{14}H_{23}NO_2$), 232.1686 ($C_{15}H_{21}NO$); HRESIMS $[M+H]^+$ *m/z* 410.2553 (calcd $C_{22}H_{35}NO_6$ $[M+H]^+$ 410.2537, Δ4.0).

*Andrognathanol F (**18**):* ESI MS/MS (q-TOF) *m/z* 424.2710 ($C_{23}H_{37}NO_6$), 366.2275 ($C_{20}H_{31}NO_5$), 350.2340 ($C_{20}H_{31}NO_4$), 324.2191 ($C_{18}H_{29}NO_4$), 306.0761 ($C_{18}H_{27}NO_4$), 292.1917 ($C_{17}H_{25}NO_3$), 250.1816 ($C_{14}H_{23}NO_2$), 232.1680 ($C_{15}H_{21}NO$); HRESIMS $[M+H]^+$ *m/z* 424.2710 (calcd $C_{23}H_{37}NO_6$ $[M+H]^+$ 424.2694, Δ3.8).

*Andrognathine acetonide (**19**):* amorphous solid; ^{1}H NMR (600 MHz, d_6-DMSO) δ 4.11 (d, *J* = 8.3CH, H-10), 3.48 (m, CH_2, H-4a), 3.47 (m, CH_2, H-5a), 3.41 (t, *J* = 7.4, CH, H-11), 3.10 (m, CH, H-8), 2.92 (m, CH_2, H-5b), 2.91 (m, CH_2, H-4b), 2.26 (m, CH_2, H-7a), 1.95 (m, CH_2, H-6b), 1.88 (m, CH_2, H-6a), 1.74 (m, CH_2, H-7b), 1.72 (m, CH, H-12), 1.66 (m, CH, H-2), 1.63 (m, CH, H-9), 1.34 (s, CH_3, H-16), 1.27 (s, CH_2, H-17), 1.21 (m, CH_2, H-3), 1.05 (d, *J* = 5.8, CH_3, H-1), 0.93 (d, *J* = 6.4, CH_3, H-13), 0.84 (d, *J* = 5.8, CH_3, H-14); ^{13}C NMR (125 MHz, d_6-DMSO) δ 107.9 (C, C-15), 83.2 (CH, C-11), 76.6 (CH, C-10), 65.4 (CH, C-8), 49.7 (CH_2, C-4), 49.7 (CH_2, C-5), 49.7 (CH_2, C-6), 45.0 (CH, C-9), 31.9 (CH, C-2), 31.6 (CH, C-12), 28.7 (CH_2, C-3), 27.4 (CH_2, C-7), 27.1 (CH_3, C-16), 26.3 (CH_2, C-17), 18.9 (CH_3, C-14), 18.8 (CH_3, C-1), 18.5 (CH_3, C-13).

*Andrognathanol hydrate (**21**):* amorphous solid; ^{1}H NMR (600 MHz, d_6-DMSO) δ 3.86 (m, CH, H-6), 3.57 (d, *J* = 6.0, CH, H-1),3.48 (d, *J* = 4.5, CH, H-11), 2.86 (d, *J* = 11.8, CH_2, H-3b), 2.38 (t, *J* = 11.7, CH_2, H-4b), 2.26 (t, *J* = 13.1, CH_2, H-4a), 2.11 (dd, *J* = 5.4 and 11.8, CH_2, H-3a), 2.01 (d, *J* = 11.3, CH, H-8), 1.81 (m, CH_2, H-14b), 1.73 (m, CH_2, H-13b),1.72 (m, CH, H-2), 1.57 (m, CH, H-10),1.53 (m, CH_2, H-5b), 1.50 (m, CH_2, H-5a),1.50 (m, CH, H-9), 1.42 (d, *J* = 12.3, CH_2, H-7b),1.26 (m, CH_2, H-7a), 1.18 (m, CH_2, H-14a), 0.91 (m, CH_2, H-13a), 0.76 (s, CH_3, H-15); ^{13}C NMR (d_6-DMSO; through HSQC and HMBC) δ 76.3 (C-1), 75.8 (C-11), 62.4 (C-6), 57.4 (C-8), 56.1 (C-3), 49.4 (C-4), 44.0 (C-2), 43.7 (C-10), 35.5 (C-7), 31.9 (C-5), 30.5 (C-9), 29.9 (C-14), 29.9 (C-13), 19.7 (C-15); HRESIMS $[M+H]^+$ *m/z* 268.191 (calcd $C_{15}H_{25}NO_3$ $[M+H]^+$ 268.1904, Δ1.1).

Hydrogenation and Hydrolysis of Crude Millipede Extract. An aliquot of the *A. corticarius* (10 mg) crude extract was hydrogenated using 10% Pd/C (23 mg, 0.0216 mmol) in EtOAc (2 mL) under H_2 (g) and left stirring at rt for 16 h. The mixture was then filtered through a celite glass plug and dried down under vacuum. This mixture was then resuspended in 1 N NaOH (3 mL), heated to 40° C in an oil bath, and left stirring for 48 h. The reaction mixture was quenched by treatment with 1 N HCl *(aq)*. The solution was then extracted with EtOAc (2 mL x3) The organic layer and aqueous layer were dried down separately under vacuum. The dried aqueous material was resuspended in 50%

MeCN/50% H_2O at 5 mg/mL and the hydrolysis products were purified by HPLC equipped with a Phenomenex Luna 5 µm Phenyl-Hexyl (250 × 10 mm) column under the following conditions: holding 0% MeCN + 0.1% FA/100% H_2O + 0.1% FA for 10 min followed by a linear gradient to 50% MeCN + 0.1% FA/50% H_2O + 0.1% FA over 10 min with a flow rate of 3 mL/min. This yielded a pure hydrogenated hydrolyzed andrognathine backbone (10.1 min, 0.4 mg, 0.002 mmol) and a hydrolyzed andrognathanol backbone (18.7 min, 1.4 mg, 0.0052 mmol). It is worth noting that only one peak for each compound was observed on the LCMS, thus indicating all andrognathanols and andrognathines contain the same relative configuration.

Acetonide Protection of Andrognathine Core (19). An aliquot of the hydrogenated hydrolyzed andrognathine backbone (0.4 mg, 2 µmol) was reacted with *p*-toluenesulfonic acid (26 mg, 0.15 mmol) and 2,2-dimethoxypropane (0.20 mL, 1.6 mmol) at rt under N_2 (g) for 16 h. The reaction was quenched by the addition of 1 N NaOH. The solution was extracted with EtOAc (3 mL x3) and the organic layer was extracted with brine (4 x 3 mL). The organic layer was dried under vacuum and the acetonide product (**19**) was isolated by HPLC equipped with a Phenomenex 4 µm Hydro column (250 × 10 mm) under the following conditions: holding10% MeCN + 0.1% FA/90% H_2O + 0.1% FA for 5 min followed by a linear gradient to 55% MeCN + 0.1% FA/45% H_2O + 0.1% FA over 15 min with a flow rate of 3 mL/min, yielding pure **19** (17 min).

Andrognathine Benzoate (20) for ECD. An aliquot of the millipede crude extract (300 µL) was reacted with 0.5 N NaOH (300 µL), heated to 40 °C in an oil bath, and left stirring overnight. The reaction mixture was quenched by treatment with 1 N HCl *(aq)*. The solution was then extracted with EtOAc (1 mL x 3) and the aqueous layer was dried under vacuum. The crude mixture was then resuspended in THF (5 mL) and treated with bromobenzoyl chloride (30 mg, 0.13 mmol), and DMAP (10 mg, 0.081 mmol). The mixture was left stirring under N_2 for 48 h. The solution was then extracted with EtOAc (1 mL x 3) and washed with brine (1 mL). The combined organic layer was dried under vacuum. The dried organic material was resuspended in 50% MeCN/50% H_2O and the esterified products were purified by HPLC equipped with a Phenomenex 4 µm Hydro column (250 × 10 mm) column under the following conditions: holding 45% MeCN + 0.1% FA/55% H_2O + 0.1% FA for 15 min followed by a linear gradient to 100% MeCN + 0.1% FA over 15 min with a flow rate of 3 mL/min, yielding pure **20** (28.6 min).

GCMS Analysis of Fatty Acid Composition in *A. corticarius* Crude Extract. The organic layer from the hydrolysis reaction described above was suspended in EtOAc (1.5 mL), followed by the addition of (trimethylsilyl)diazomethane (6 drops of 0.6 M in hexanes). The reaction mixture was stirred at rt for 15 min, then dried under N_2 (g). The product, along with commercial standards of methyl-3*R*-hydroxypentanoate and methyl-3*S*-hydroxypentanoate were analyzed by chiral GCMS equipped with a Cyclosil B column (Agilent Technologies J&W Scientific, 30 m × 0.25 mm) under the following conditions: the initial oven temp was 64 °C, kept for 20 min, followed by a ramp to 85 °C, at a rate of 1.5 °C, followed by a ramp to 200 °C, at a rate of 10 °C/min, kept for 5 min. The authentic standards eluted at: methyl-3*S*-hydroxybutanoate (21.3 min), methyl-3*R*-hydroxybutanoate (21.8 min), methyl-3*S*-hydroxypentanoate (33.1 min) and methyl-3*R*-hydroxypentanoate (34.3 min). The derivatized hydrolysis product exhibited four peaks at 21.3, 21.8, 33.1, and 34.3 min.

GCMS Analysis of Fatty Acid Composition in Andrognathanol A (13) and B (14). Similarly, an aliquot of pure **13** (200 µg, 0.49 µmol) and **14** (200 µg, 0.47 µmol) were resuspended in MeOH (100 µL), then treated with 0.5 N NaOH (100 µL) at rt for 2 d. The reaction mixture was quenched by the addition of 0.5 N HCl (100 µL), then the reaction mixture was extracted with EtOAc (1 mL x 3). The combined organic layers were dried over $MgSO_4$, filtered through celite, and dried under N_2 (g). The crude product, along with standards for 3*S*-hydroxybutanoate and 3*R*-hydroxybutanoate, was methylated by resuspension in EtOAc (1 mL) followed by the addition of (trimethylsilyl)diazomethane (4 drops, 0.6 M in hexanes). The reaction mixtures were stirred at rt for 15 min, then dried under N_2 (g). The products were analyzed by chiral GCMS using the method described above. The derivatized hydrolysis product of **13** exhibited two peaks at 21.3 and 21.8 min, while the derivatized hydrolysis product of **14** exhibited a single peak at 33.1 min.

DFT Calculation of ECD Spectra for Andrognathanols. Conformational analyses were carried out using ComputeVOA v1.1. A single lowest-energy conformer was obtained from the conformation search. Geometry, frequency, and ECD calculations were applied at the DFT (density functional theory) (B3LYP functional/DGDZVP basis set) and TD-DFT (time-dependent density functional theory) [B3LYP functional/6-311G(d,p) basis set] levels with Gaussian' 16 carried out in MeCN phase using the COSMO solvation model. The calculated ECD spectrum of the lowest-energy conformer was adjusted by applying a sigma value of 0.3 eV and a 5 nm shift to the high-wavelength

side prior to the comparison with the experimentally obtained ECD data. The conformation search returned only one predominant conformer with the energy cutoff 3 kcal/mol.

LCMS Quantification of Andrognathanols and Andrognathines. Thirty individual *A. corticarius* were collected and placed in 1.5 mL Eppendorf tubes. Each specimen was measured and preserved in 0.5 mL of MeOH. After initial preservation, the MeOH was removed, and each millipede was rinsed three times with ~1 mL of fresh MeOH per rinse. The pooled MeOH for each individual was dried under vacuum and reconstituted in 200 µL of 50% MeOH/50% H_2O. A concentration curve for andrognathine A (**1**), andrognathanol A (**13**), and andrognathanol B (**14**) was generated using authentic standards at the following concentrations: **1** - 7.000, 3.500, 1.750, 0.870, 0.430, 0.220, 0.110, 0.050, and 0.025 µg/µL; **13** and **14** - 1.750, 0.870, 0.430, 0.220, 0.110, 0.050, and 0.025 µg/µL. All standards were analyzed at both the beginning and end of the millipede sample sequence. A small aliquot (5 µL) was taken from each millipede sample and standard was analyzed by LR-LCMS (LTQ) using a Kinetex 5µm EVO C18 (100 x 3.0 mm) column using the following solvent gradient system: holding 10% MeCN + 0.1% FA/90% H_2O + 0.1% FA for 3 min followed by a gradient to 100% MeCN + 0.1% FA over 11 min with a flow rate of 0.3 mL/min. Raw LC-MS data files were converted to .mzML format using MSConvert (v3.0.20337) and analyzed in MZmine 3 (v3.9.0). Mass detection was performed with an MS1 noise threshold of 3.5×10^5 and an MS2 threshold of 1×10^2. The ADAP chromatogram builder used the following parameters: minimum group size = 7, group intensity threshold = 3.5×10^5, minimum peak height = 1×10^2, and m/z tolerance = 0.5. Peak deconvolution was performed using the local minimum resolver with a chromatographic threshold of 97%, RT search window = 0.2 min, minimum relative height = 10%, minimum absolute height = 3.5×10^3, minimum peak top/edge ratio = 1.15, minimum number of data points = 7, and peak duration range = 0.0–3.0 min. The ^{13}C isotope filter was applied with an *m/z* tolerance of 0.1, RT tolerance of 0.1 min, maximum charge = 3, and the most intense isotope selected as the representative. The join aligner was configured with an m/z tolerance of 0.2, retention time tolerance of 1 min, and weighting factors of 75 for *m/z* and 25 for RT. Quantitative data were exported as .csv files. Peak areas were extracted for the $[M+H]^+$ ions corresponding to each compound of interest (m/z 352, 366, 380, 394, 408, 410, and 424). Calibration curves were constructed by averaging the peak areas from the standard injections and were used to quantify andrognathines and andrognathanols in individual millipedes. Finally, the number of ozopores in each specimen was counted and plotted against the extracted compound masses using GraphPad Prism (v10.2.0).

Ant Predator Assay. *Aphaenogaster* sp. were collected from the same location where *A. corticarius* is known to be found. To assess the potential repellent effects of andrognathines and andrognathanols on a likely predator, groups of four ants were placed in 100 mm × 15 mm Petri plates containing a paper disc treated with ~400 µg of crude extract from a single millipede, dissolved in 50% MeOH/H_2O. Additional treatments included paper discs impregnated with ~400 µg of individual compounds **1**, **13**, and **14**, glucose, or α-pinene, each dissolved in ~100 µL of the same solvent. Negative controls included discs treated with solvent only, glucose in 50% MeOH/H_2O, and untreated blank discs. Ant behavior was recorded for 15 min per trial, with the first two min excluded from analysis to allow for acclimation. Videos were analyzed using ToxTrac software to quantify average time spent immobile (frozen), average movement speed, and time spent within 5 cm of the treated disc.

Sigma-1 and Sigma-2 Receptor Assay Procedure from Psychoactive Drug Screening Program (PDSP). HEKT cell lines were used to make membrane pallets for binding assays. In the primary binding assays, compounds were tested at a single concentration (10 µM) and in quadruplicate in 96-well plates. Compounds showing a minimum of 50% inhibition at 10 µM were tagged for secondary radioligand binding assays to determine equilibrium binding affinity at specific targets. In secondary binding assays, compounds were tested at 11 concentrations (0.1, 0.3, 1, 3, 10, 30, 100, 300, 1,000, 3,000, and 10,000 nM) and in triplicate (3 sets of 96-well plates). Both primary and secondary radioligand binding assays were carried out in a final volume of 125 µL per well in appropriate binding buffers (50 mM Tris HCl, pH 8.0, rt). Pentazocine was used as a radioligand and the hot ligand concentration was determined close to its Kd. Total binding and nonspecific binding were determined in the absence and presence of 10 µM of haloperidol, the reference compound. Plates were incubated at room temperature and in the dark for 90 min. Reactions were stopped by vacuum filtration onto 0.3% polyethyleneimine (PEI) soaked 96-well filter mats using a 96-well Filtermate harvester, followed by three washes with cold wash buffers (50 mM Tris HCl, pH 7.4). Scintillation cocktail was then melted onto the microwave-dried filters on a hot plate and radioactivity was counted in a Microbeta counter. K_d values for [3H]-(+)-pentazocine (σ_1R: 6.70 nM), (σ_2R: 14.50) were determined via separate homologous competitive binding experiments. Ki values were determined from at least three independent experiments and are reported as mean ± SEM.

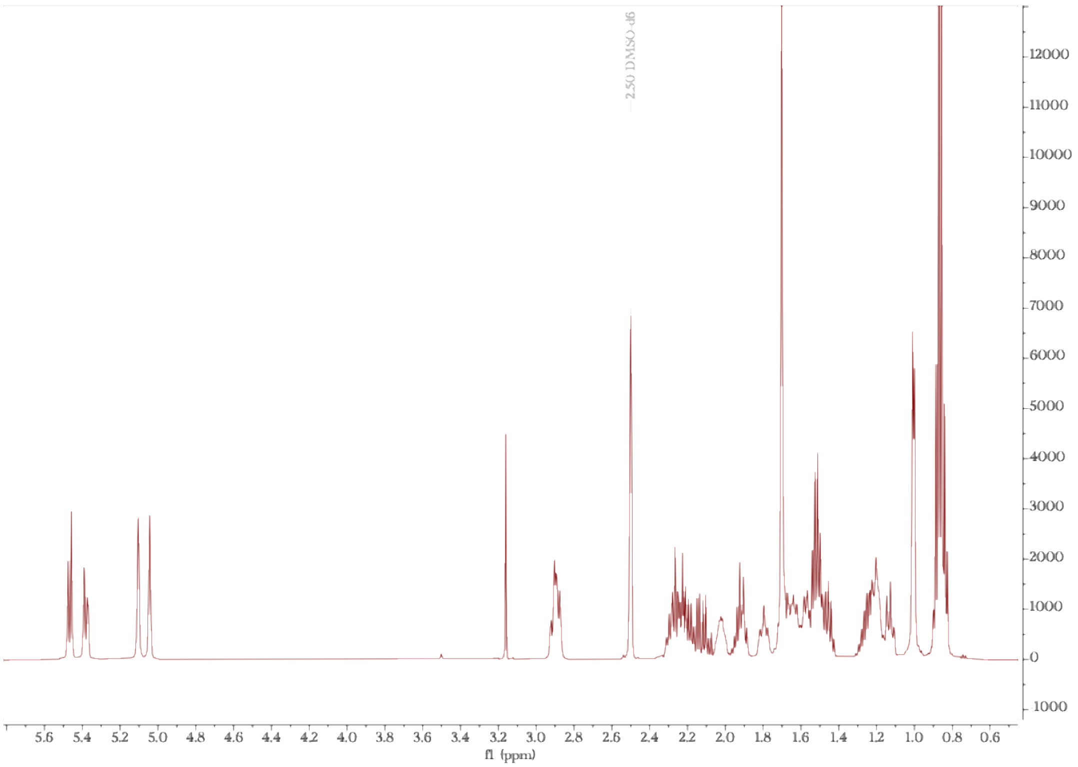

Figure S1. ^{1}H NMR spectra for andrognathine A (**1**) (600 MHz, d_6-DMSO). 64 scans

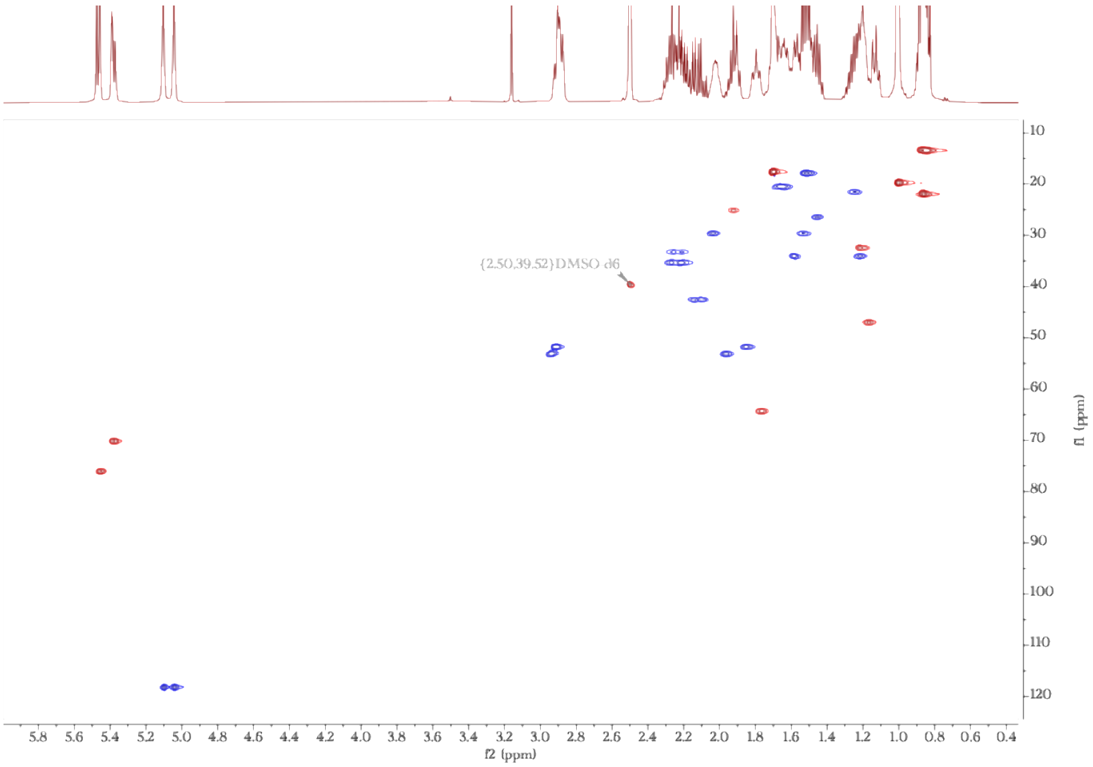

Figure S2. gHSQC NMR spectra for andrognathine A (**1**) (600 MHz, d_6-DMSO). 64 scans, NUS25 and 400 increments

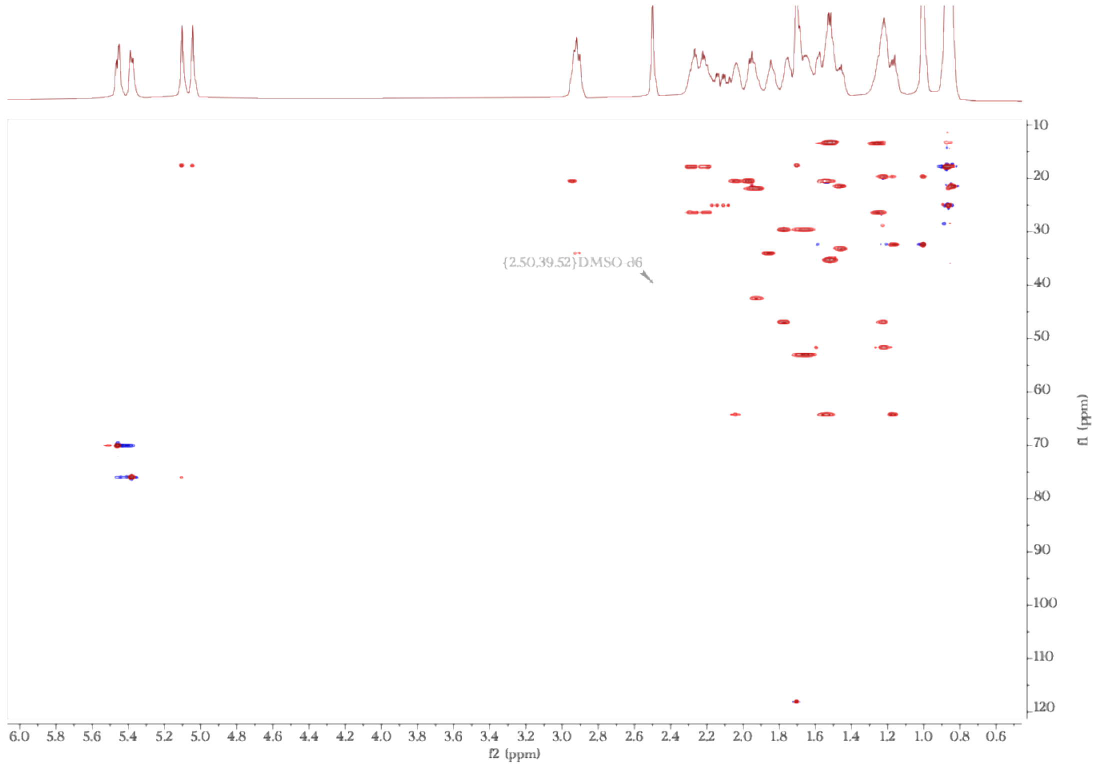

Figure S3. H2BC NMR spectra for andrognathine A (**1**) (600 MHz, d_6-DMSO). 64 scans, NUS50 and 400 increments

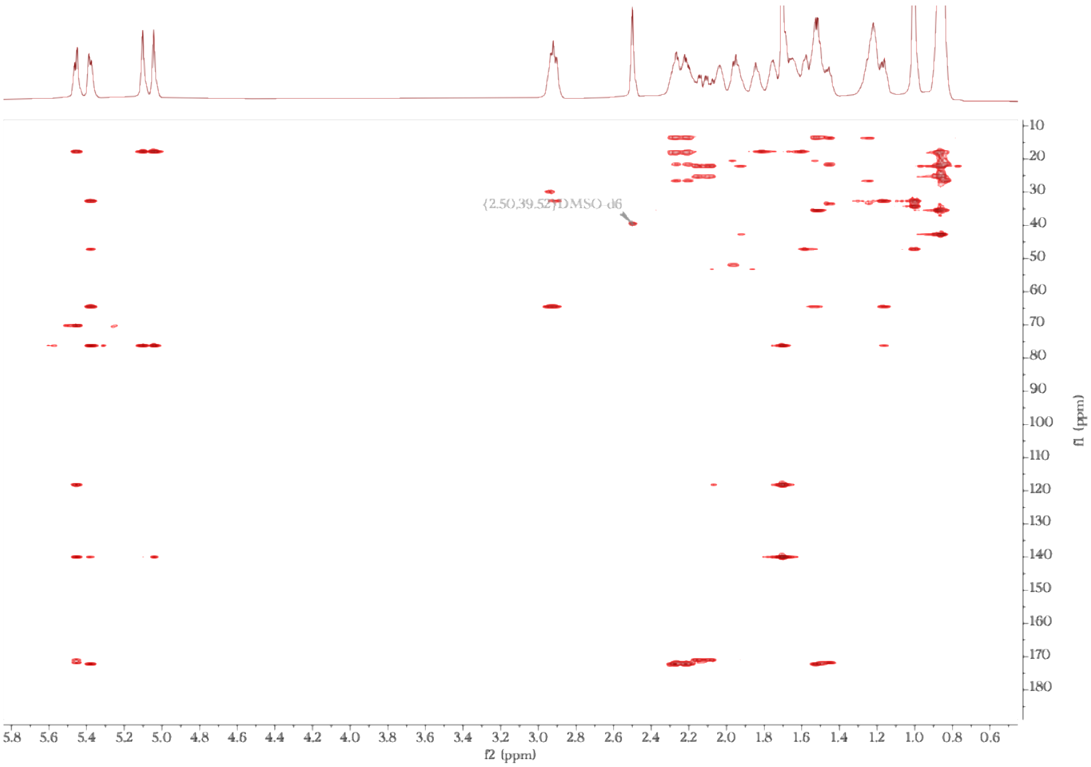

Figure S4. HMBC NMR spectra for andrognathine A (**1**) (600 MHz, d_6-DMSO). 64 scans, NUS50 and 512 increments

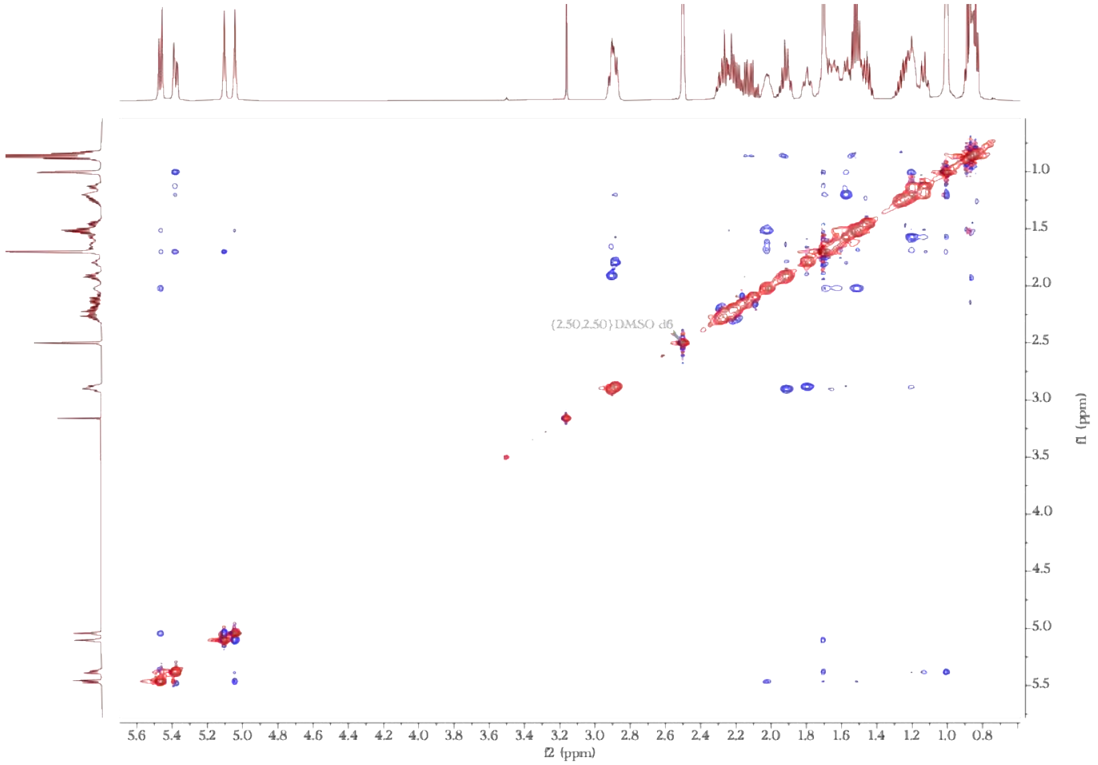

Figure S5. easyROESY NMR spectra for andrognathine A (**1**) (600 MHz, d_6-DMSO). 32 scans, NUS50 and 400 increments

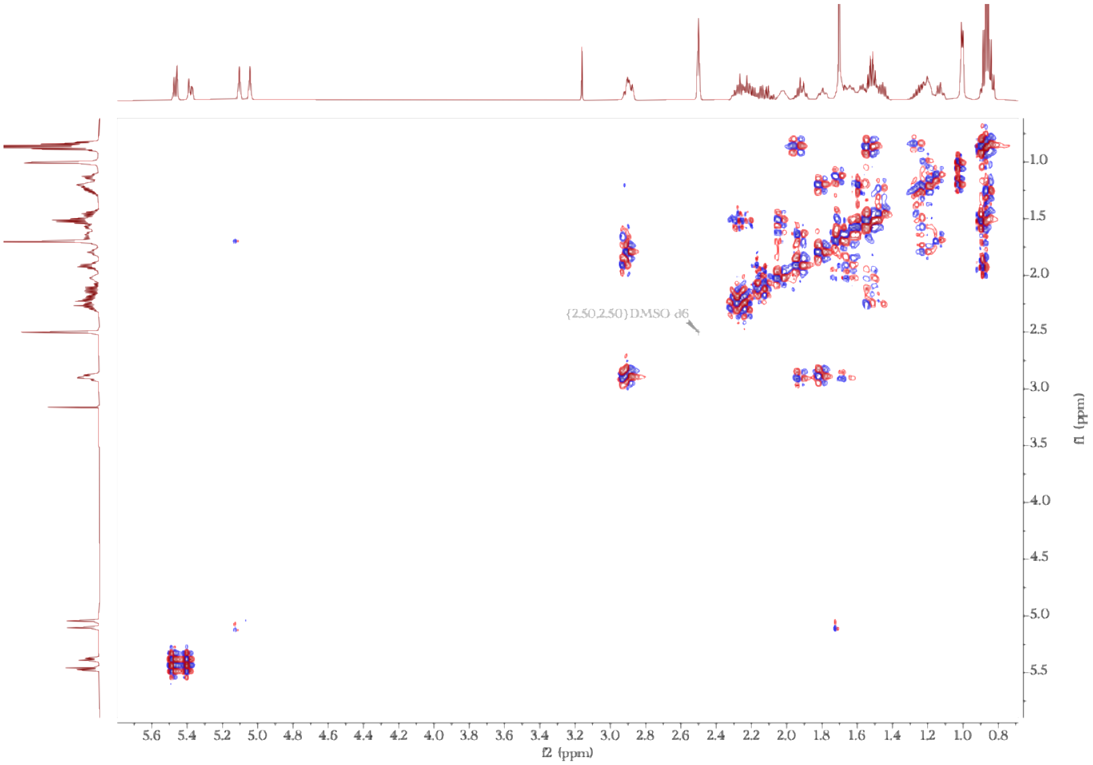

Figure S6. dqfCOSY NMR spectra for andrognathine A (**1**) (600 MHz, d_6-DMSO). 32 scans, NUS50 and 256 increments

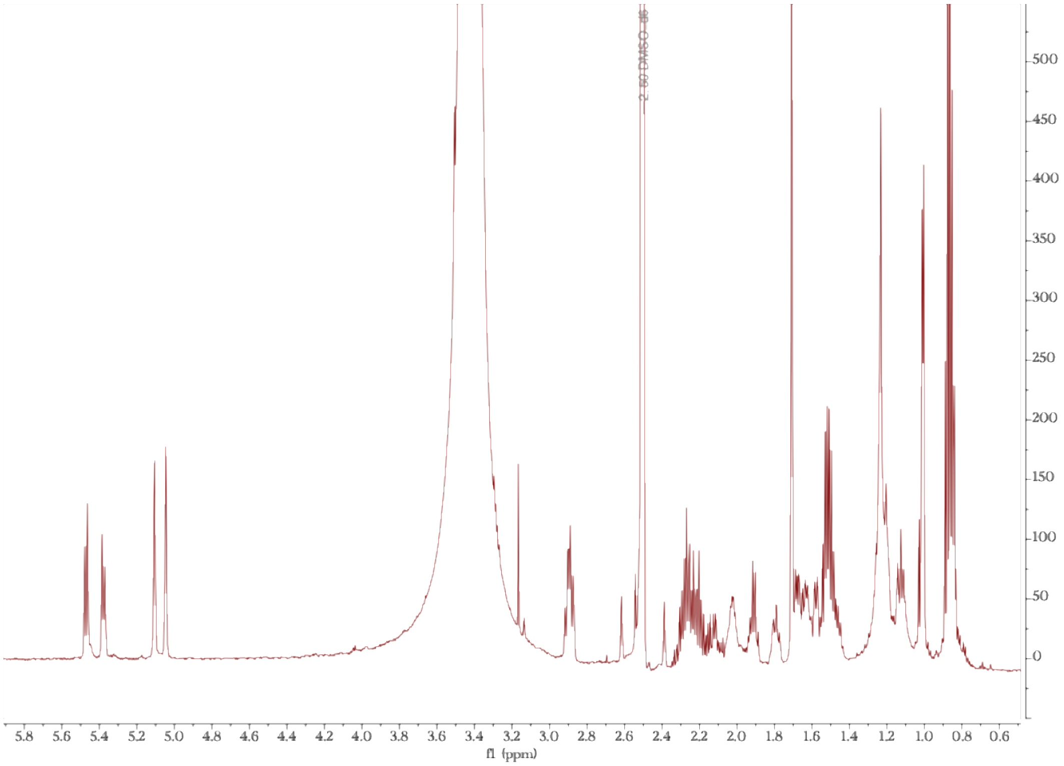

Figure S7. ^{1}H NMR spectra for andrognathine B (**2**) (600 MHz, d_6-DMSO). 64 scans

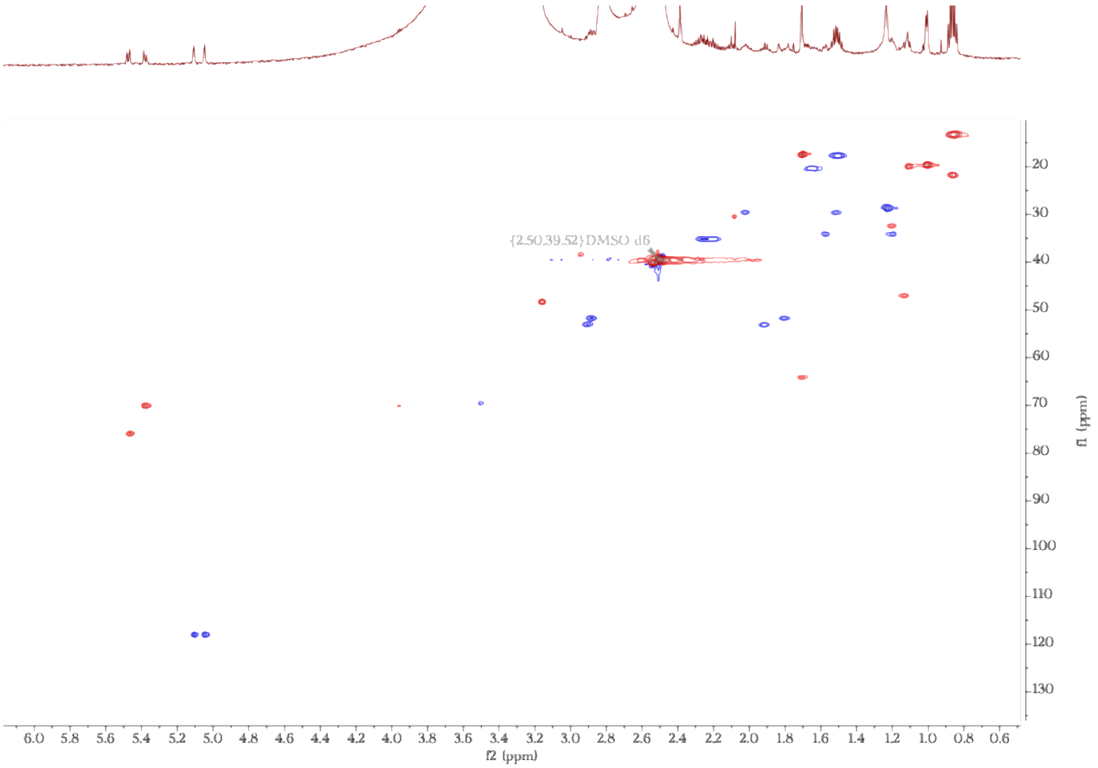

Figure S8. gHSQC NMR spectra for andrognathine B (**2**) (600 MHz, d_6-DMSO). 64 scans, NUS25 and 400 increments

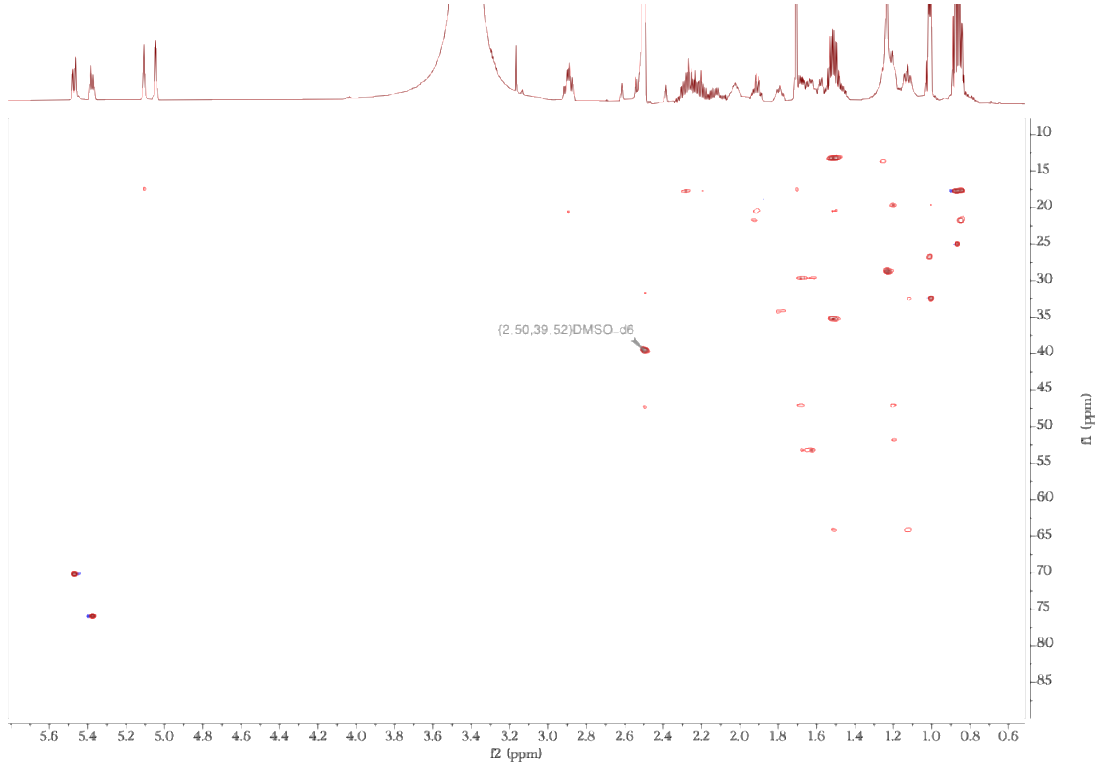

Figure S9. H2BC NMR spectra for andrognathine B (**2**) (600 MHz, d_6-DMSO). 64 scans, NUS50 and 400 increments

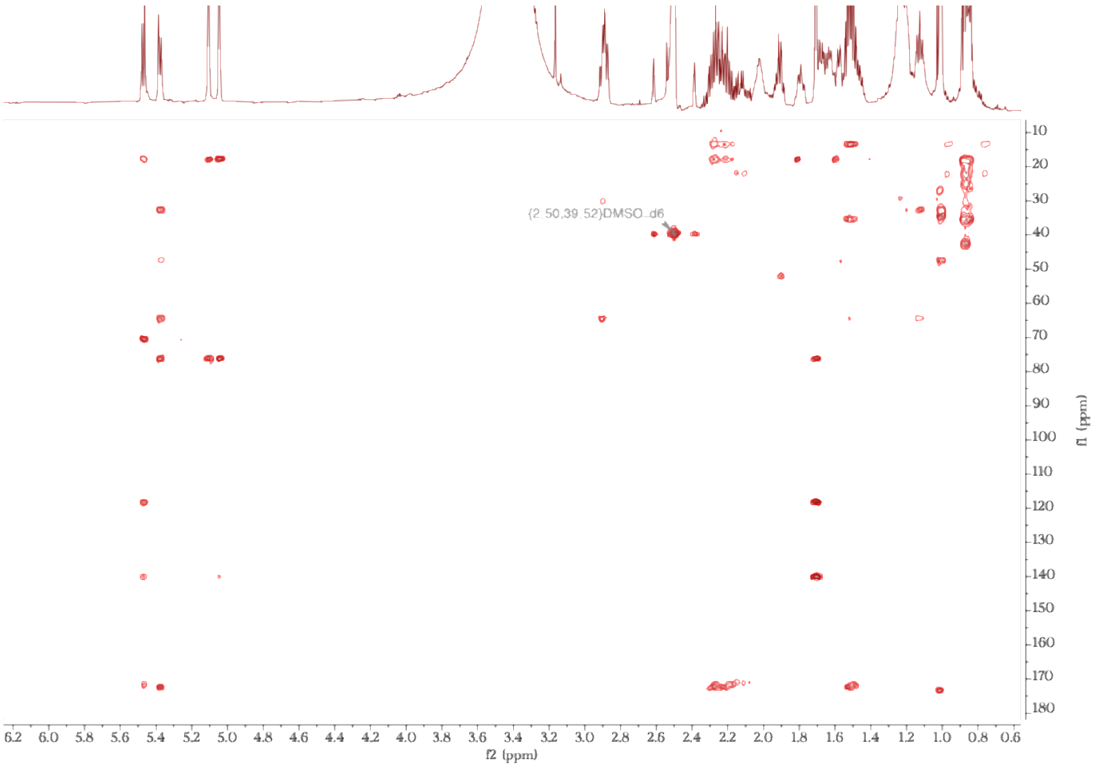

Figure S10. HMBC NMR spectra for andrognathine B (**2**) (600 MHz, d_6-DMSO). 64 scans, NUS50 and 256 increments

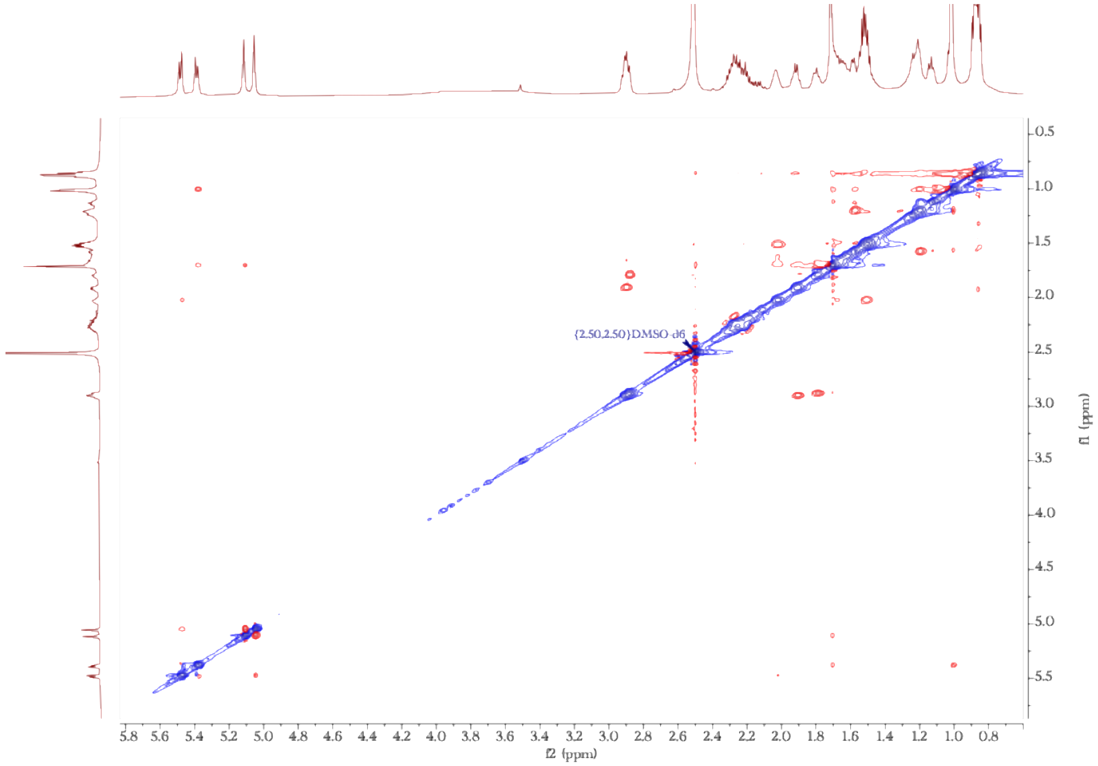

Figure S11. easyROESY NMR spectra for andrognathine B (**2**) (600 MHz, d_6-DMSO). 16 scans, NUS50 and 400 increments

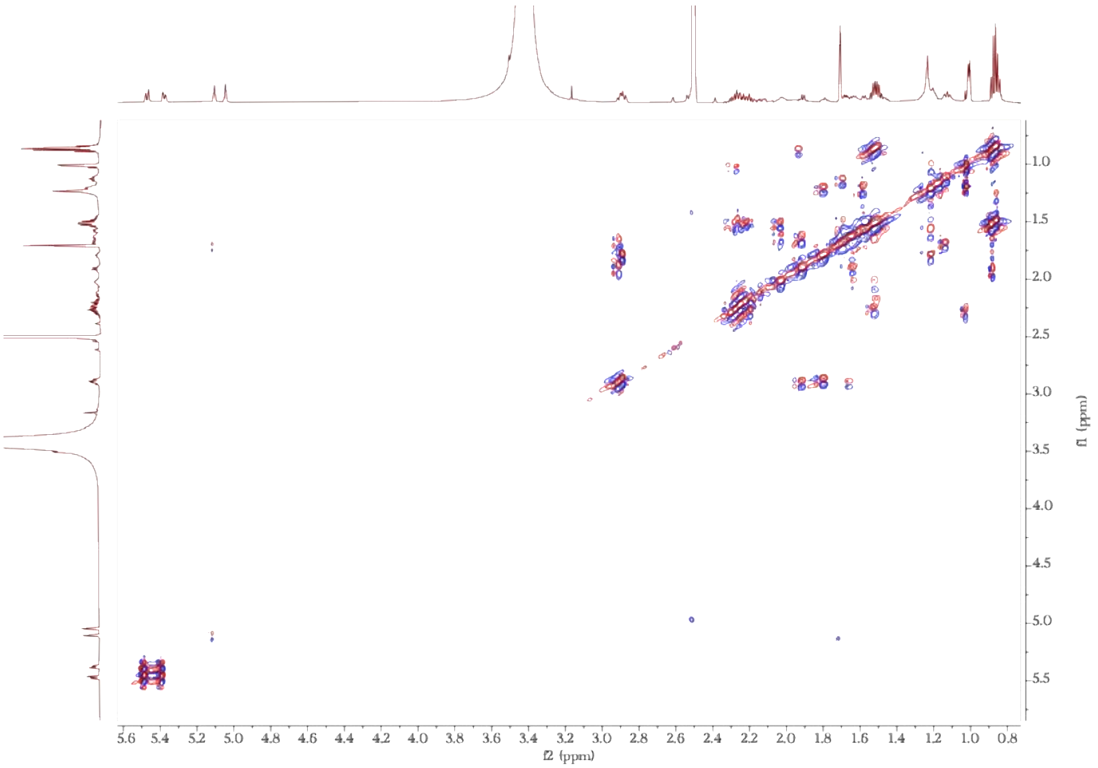

Figure S12. dqfCOSY NMR spectra for andrognathine B (**2**) (600 MHz, d_6-DMSO). 16 scans, NUS50 and 256 increments

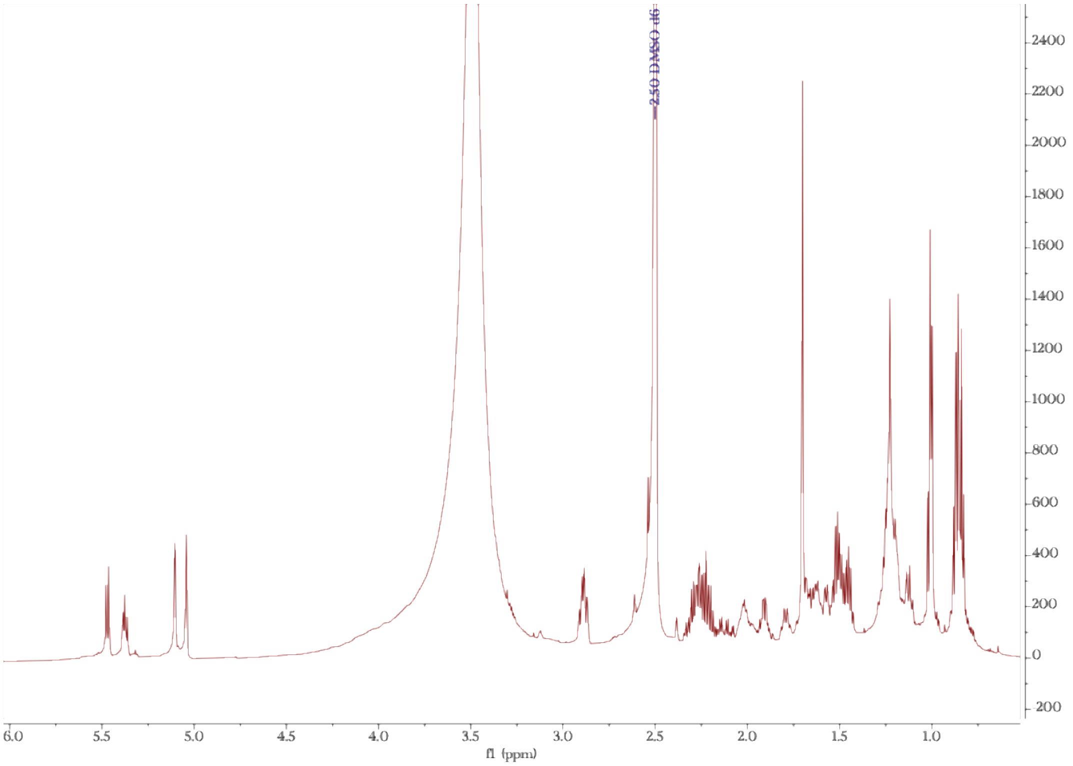

Figure S13. ^{1}H NMR spectra for andrognathine C (**3**) (600 MHz, d_6-DMSO). 128 scans

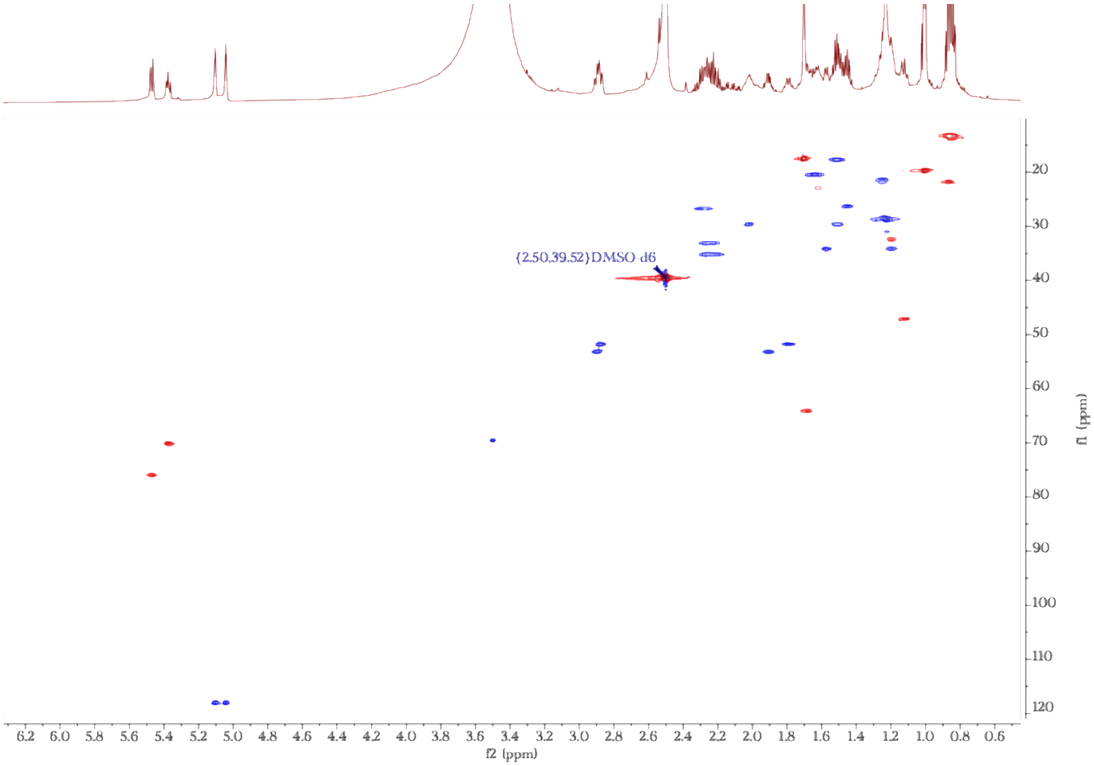

Figure S14. gHSQC NMR spectra for andrognathine C (**3**) (600 MHz, d_6-DMSO). 64 scans, NUS25 and 400 increments

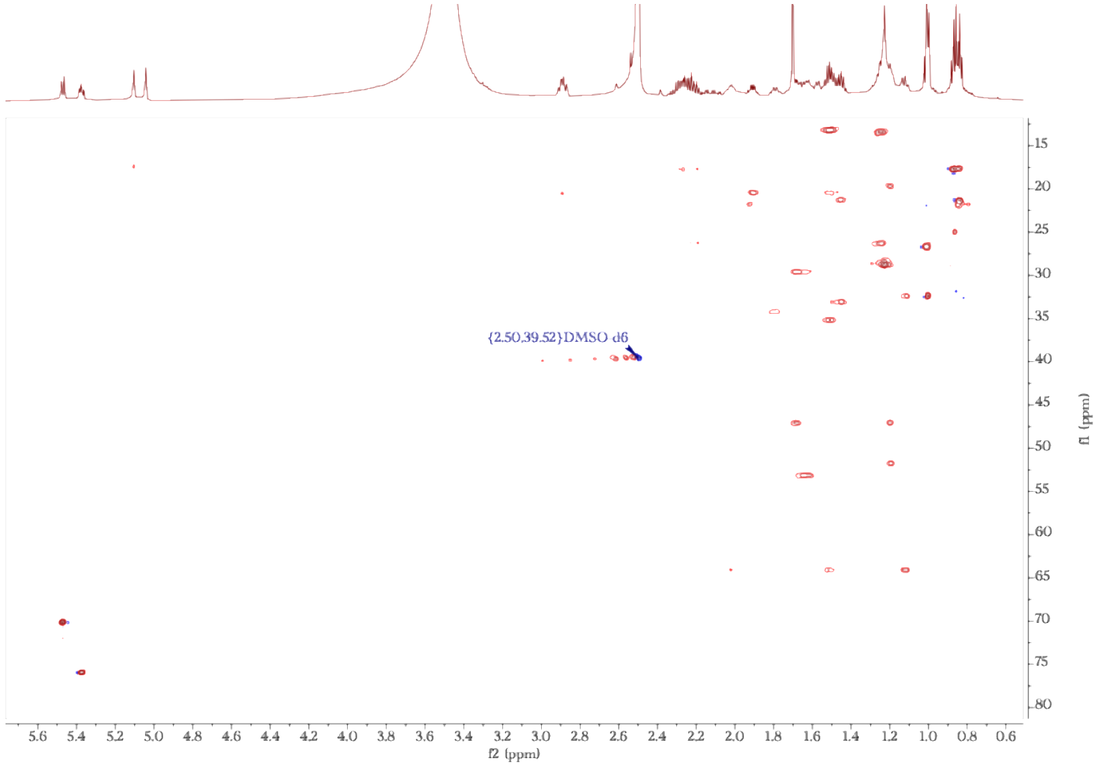

Figure S15. H2BC NMR spectra for andrognathine C (**3**) (600 MHz, d_6-DMSO). 48 scans, NUS50 and 400 increments

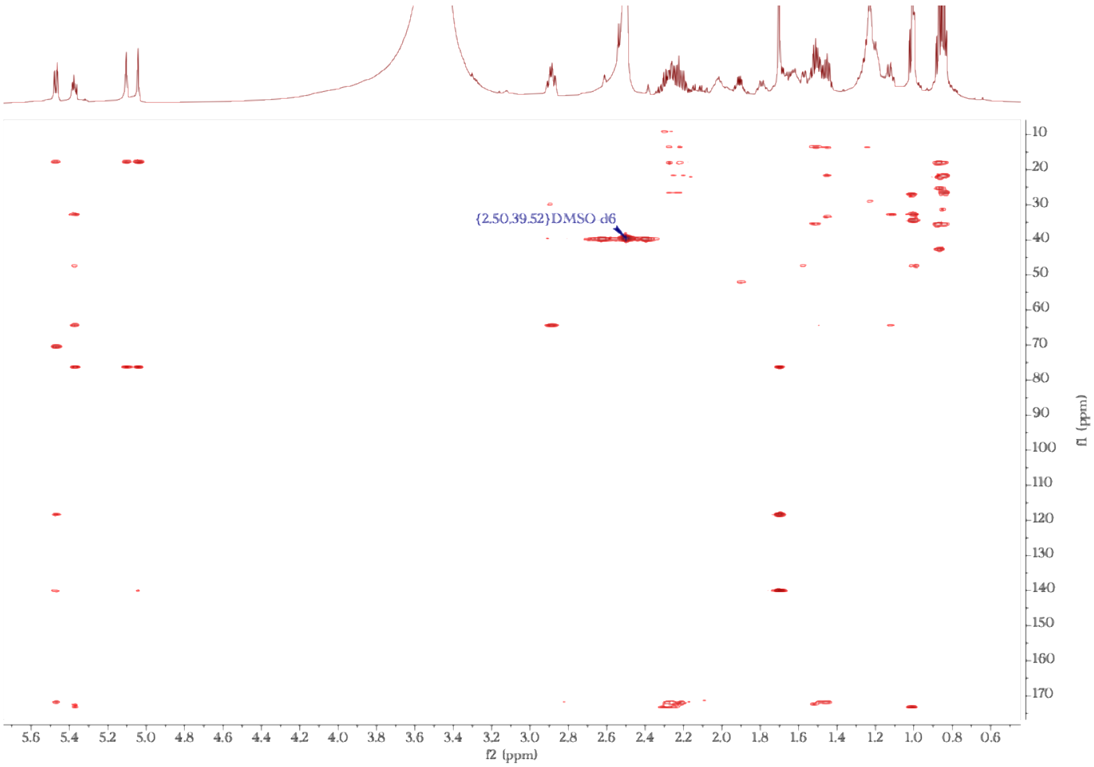

Figure S16. HMBC NMR spectra for andrognathine C (**3**) (600 MHz, d_6-DMSO). 48 scans, NUS50 and 400 increments

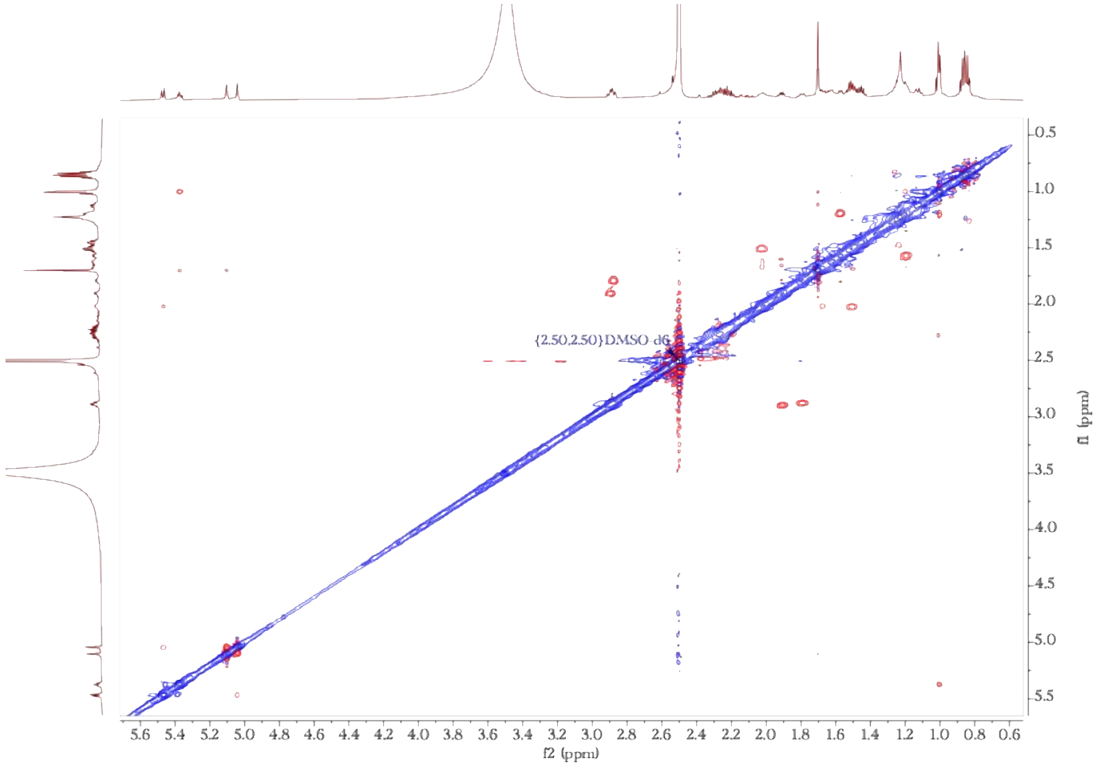

Figure S17. easyROESY NMR spectra for andrognathine C (**3**) (600 MHz, d_6-DMSO). 32 scans, NUS50 and 400 increments

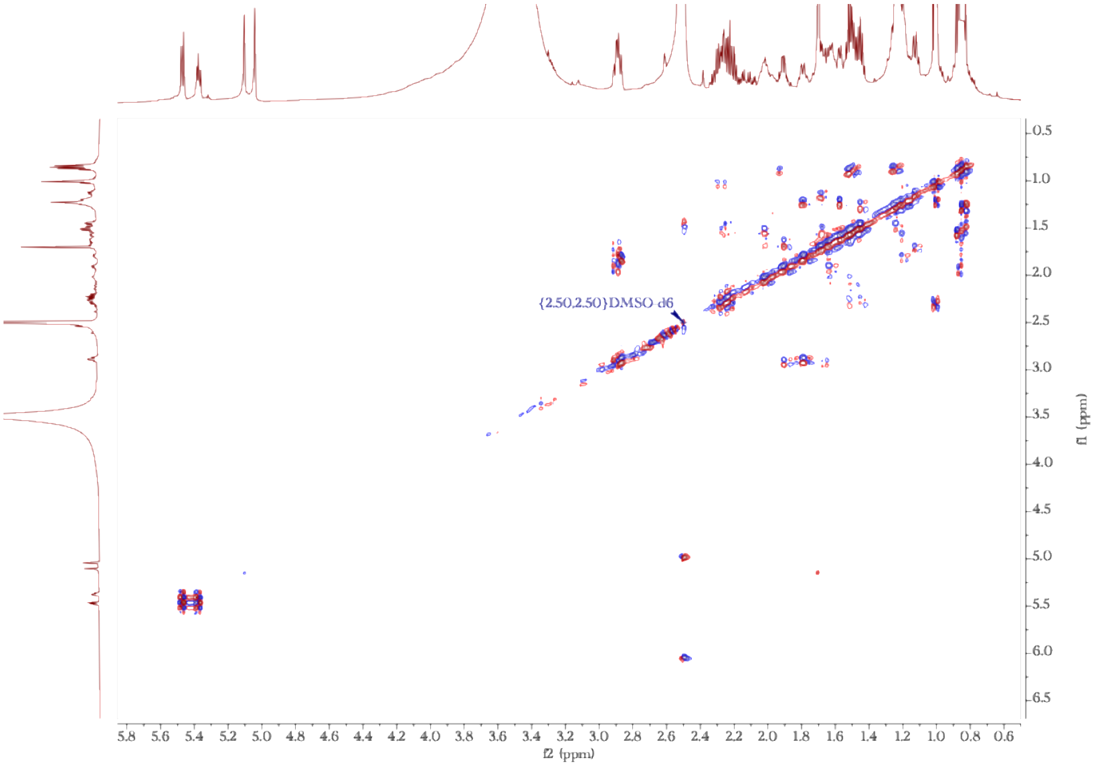

Figure S18. dqfCOSY NMR spectra for andrognathine C (**3**) (600 MHz, d_6-DMSO). 32 scans, NUS50 and 256 increments

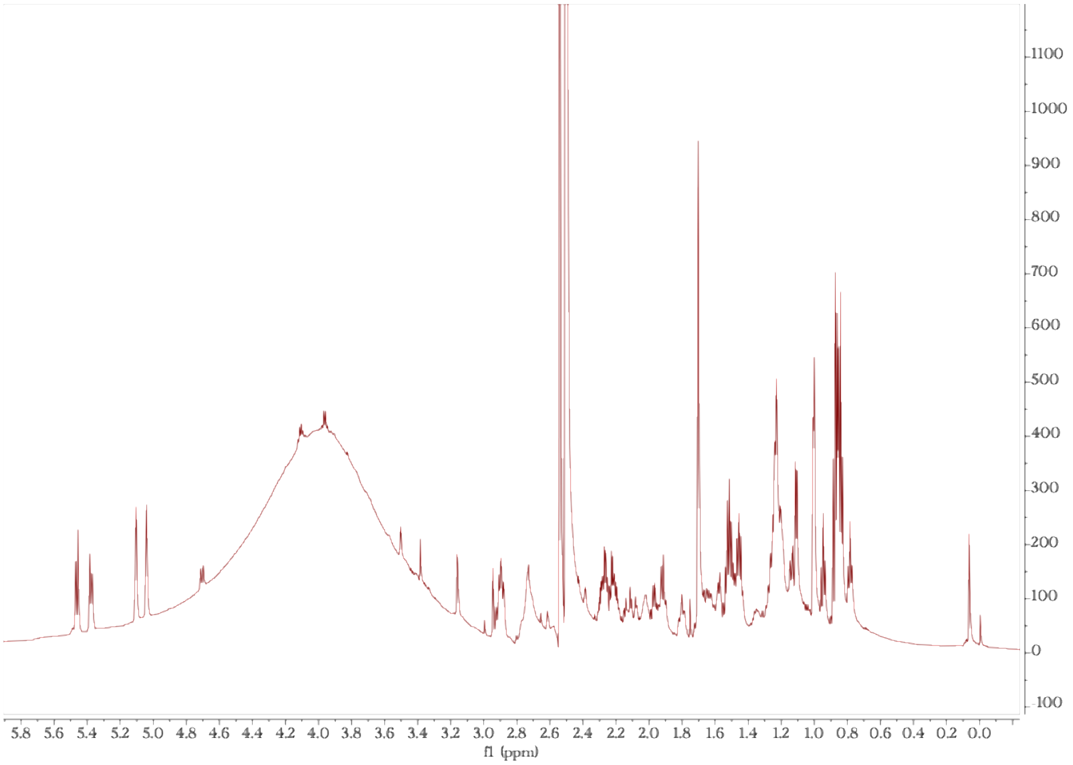

Figure S19. ^{1}H NMR spectra for andrognathine D (**4**) (600 MHz, d_6-DMSO). 128 scans

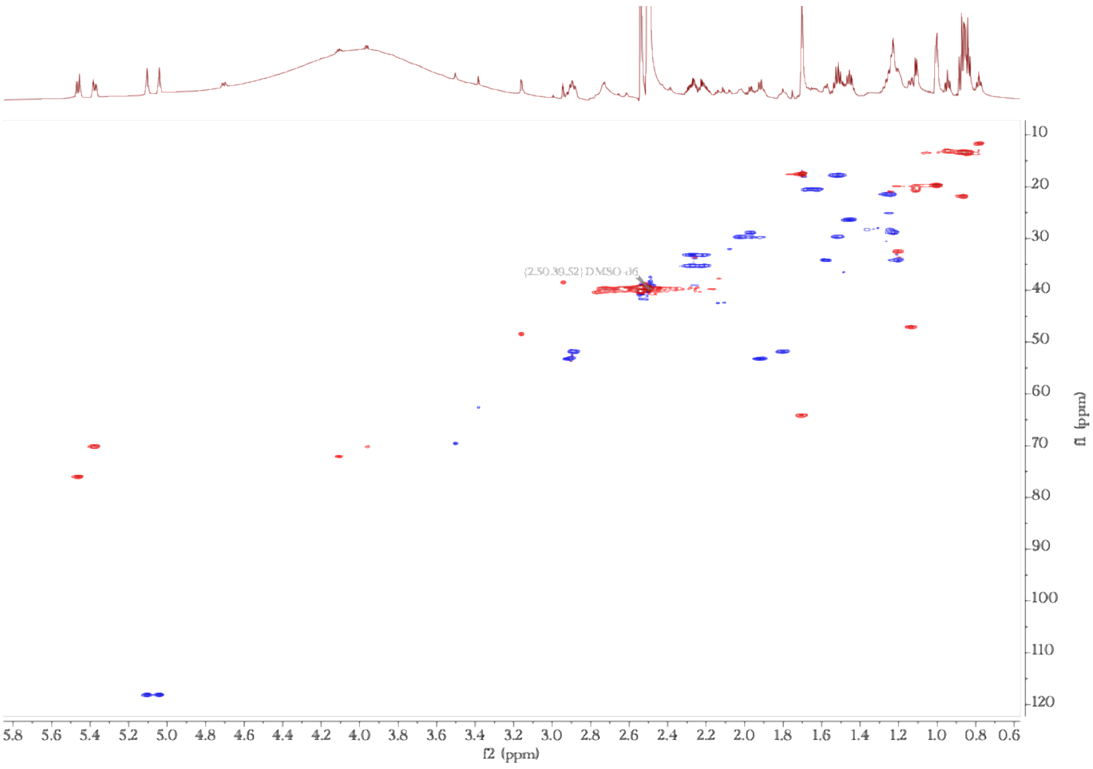

Figure S20. gHSQC NMR spectra for andrognathine D (**4**) (600 MHz, *d*$_6$-DMSO). 64 scans, NUS25 and 400 increments

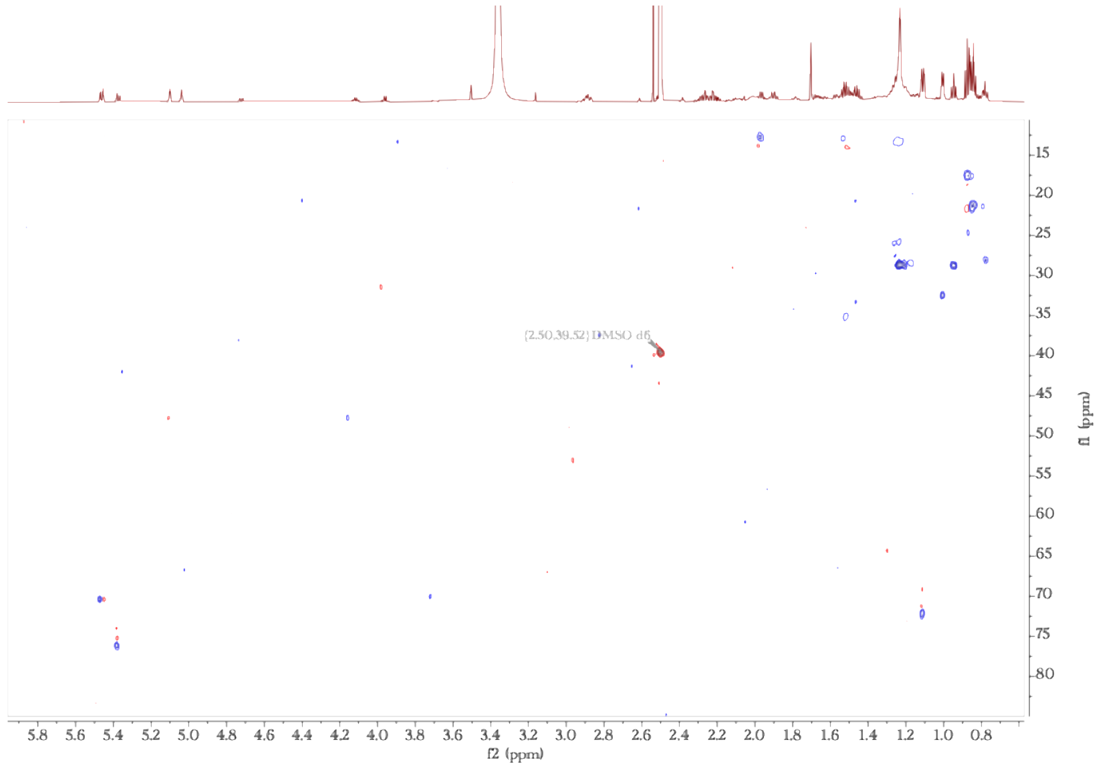

Figure S21. H2BC NMR spectra for andrognathine D (**4**) (600 MHz, *d*$_6$-DMSO). 128 scans, NUS50 and 256 increments

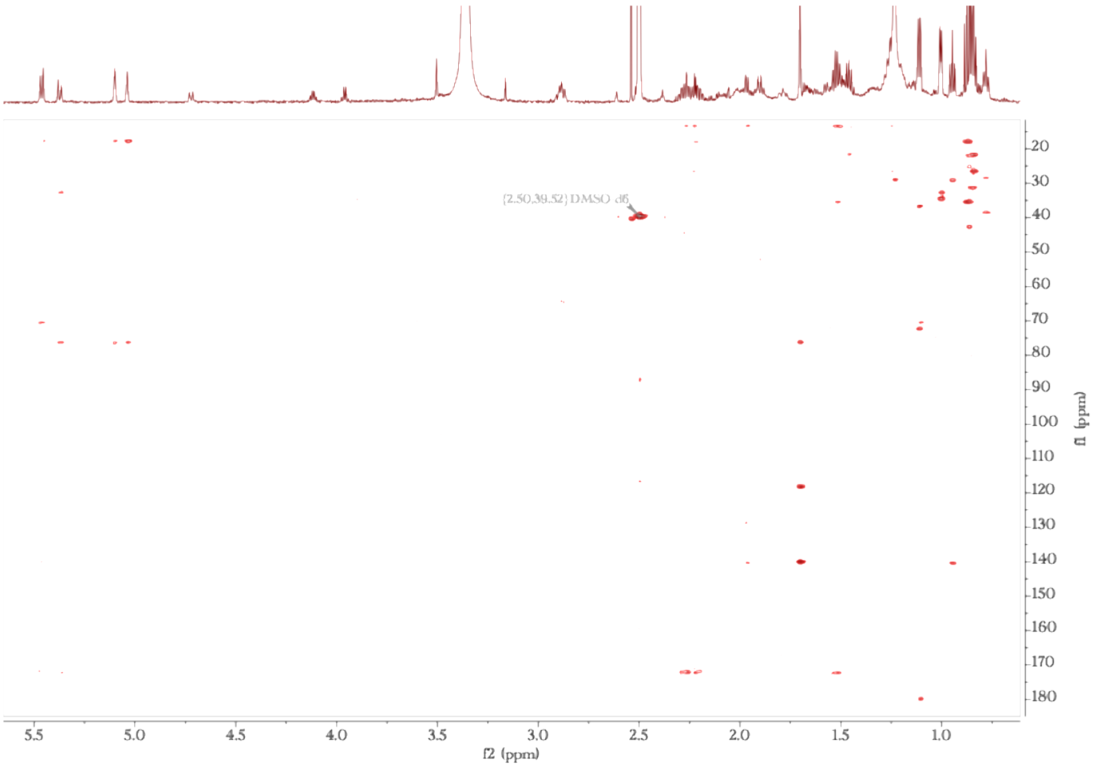

Figure S22. HMBC NMR spectra for andrognathine D (**4**) (600 MHz, d_6-DMSO). 96 scans, NUS50 and 256 increments

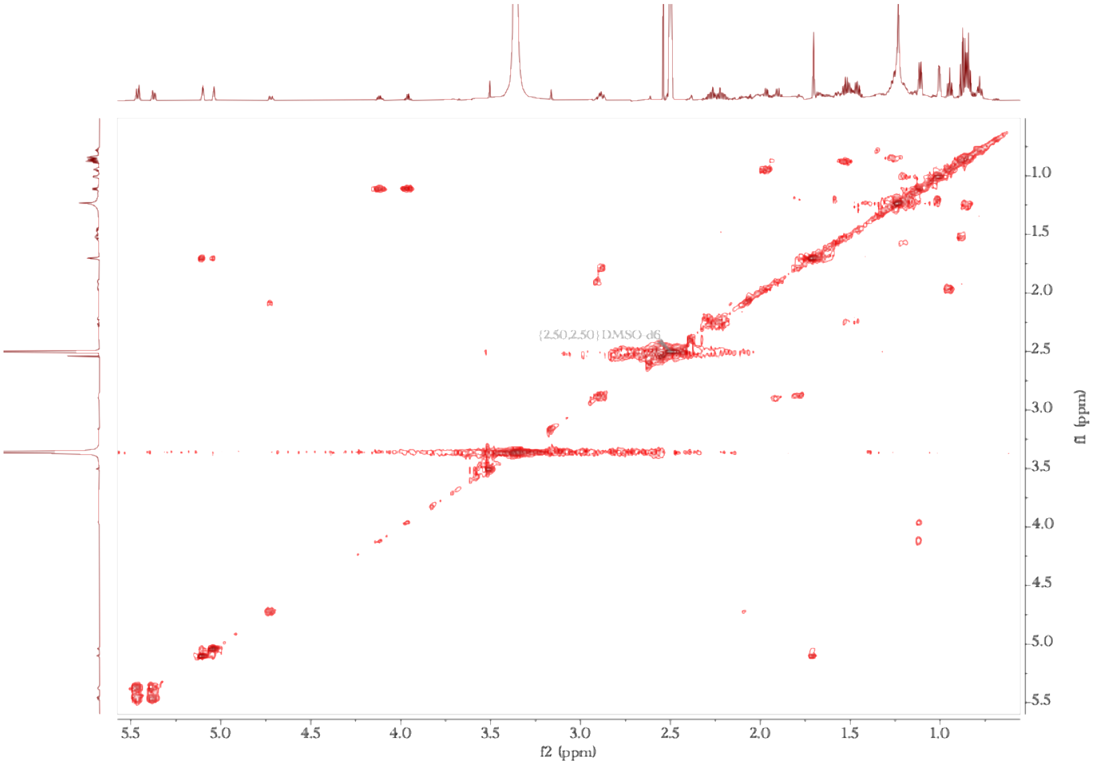

Figure S23. COSY NMR spectra for andrognathine D (**4**) (600 MHz, d_6-DMSO). 4 scans, NUS50 and 320 increments

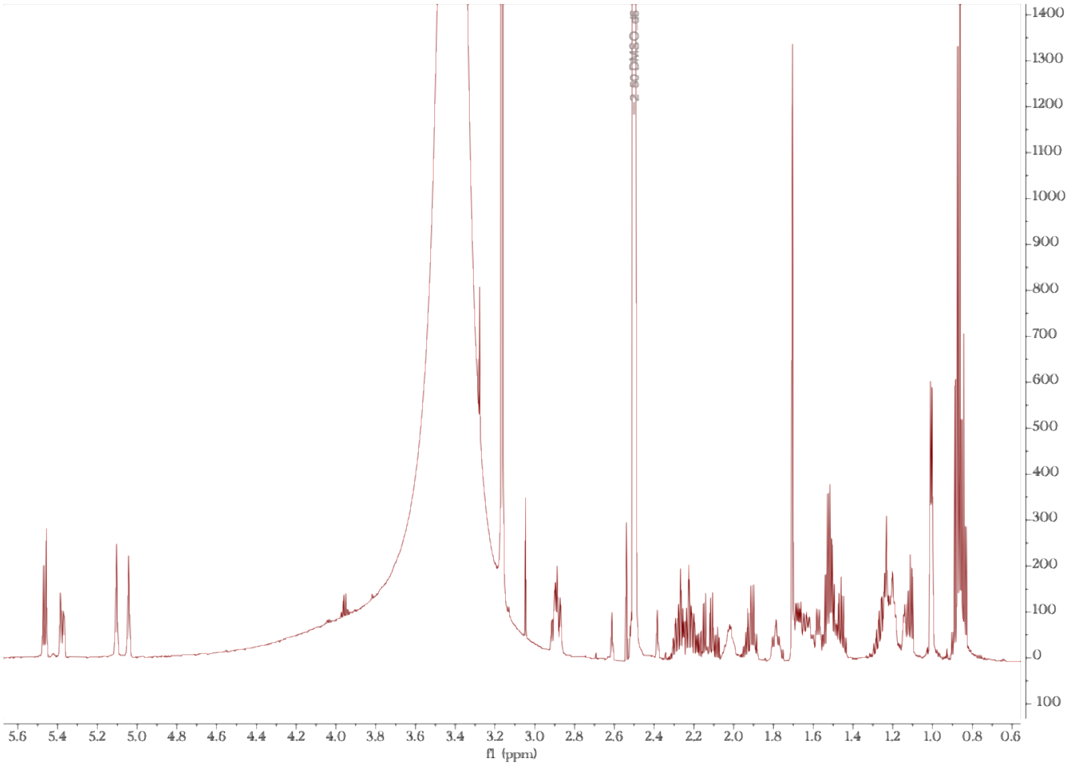

Figure S24. ^{1}H NMR spectra for andrognathine E (**5**) (600 MHz, d_6-DMSO). 128 scans

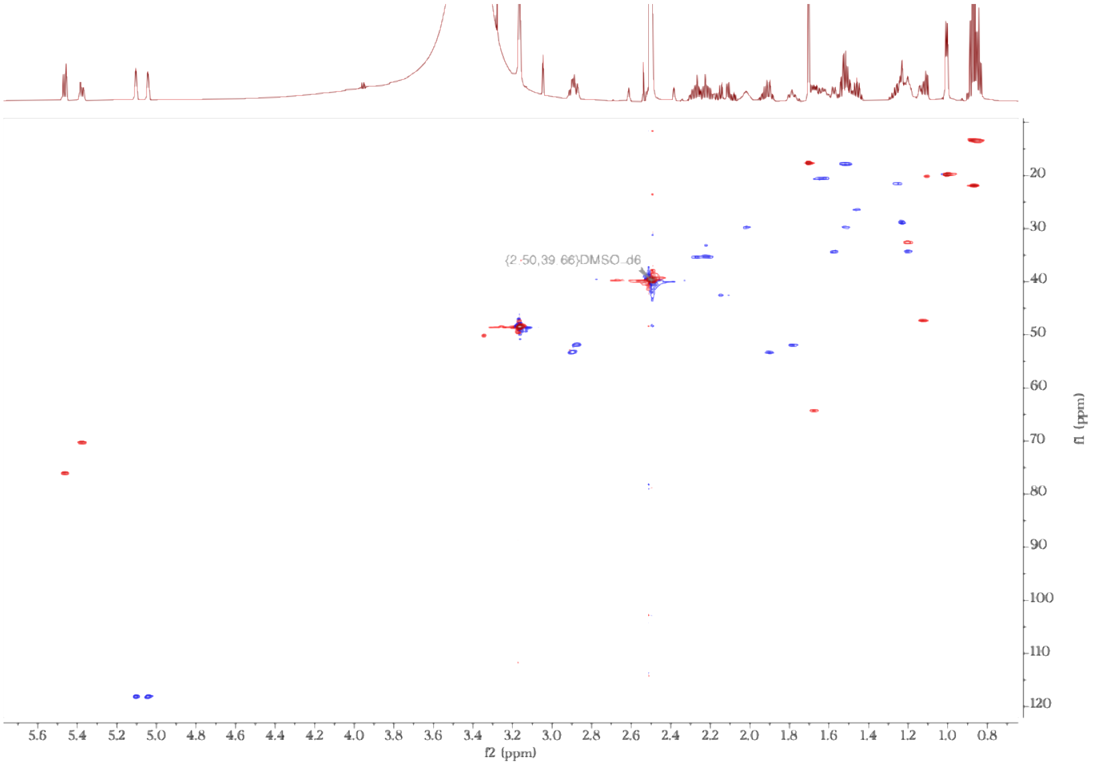

Figure S25. gHSQC NMR spectra for andrognathine E (**5**) (600 MHz, d_6-DMSO). 64 scans, NUS25 and 400 increments

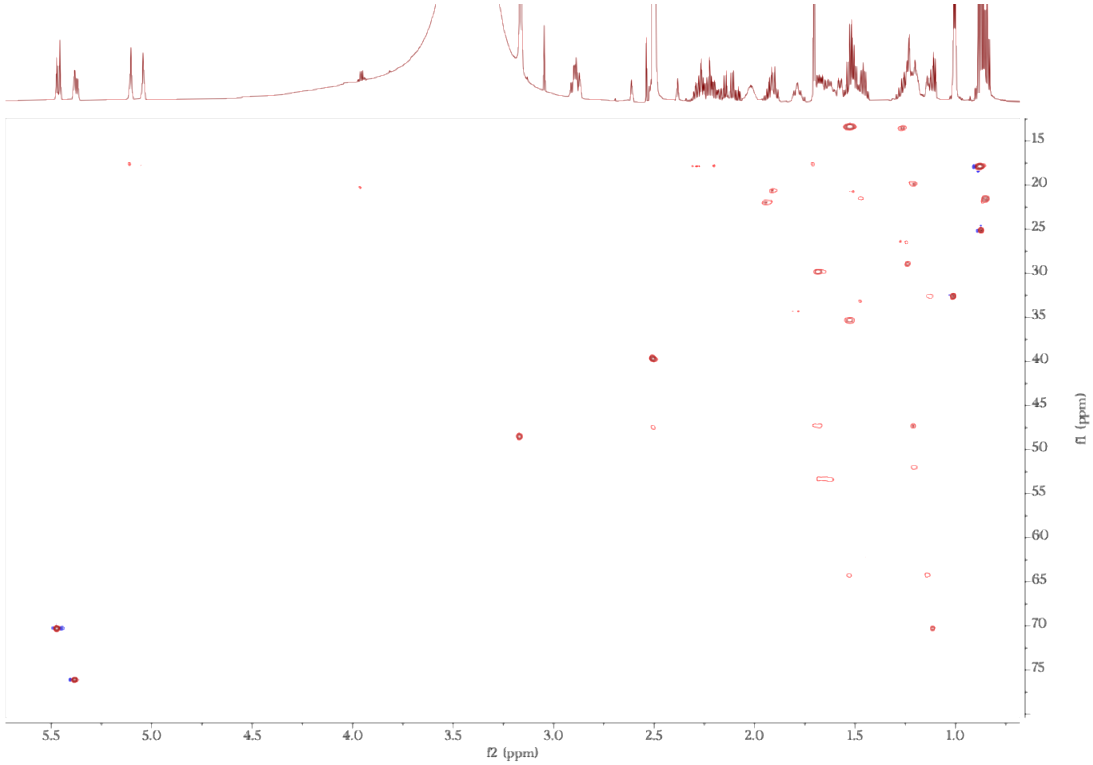

Figure S26. H2BC NMR spectra for andrognathine E (**5**) (600 MHz, d_6-DMSO). 64 scans, NUS50 and 400 increments

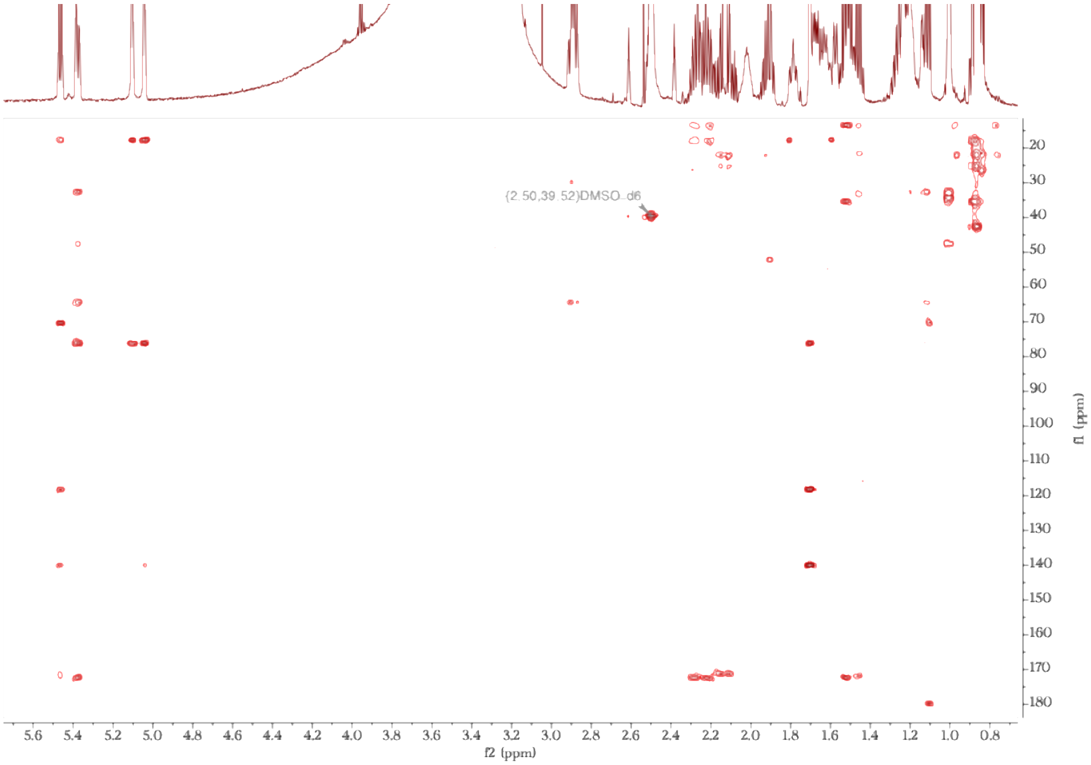

Figure S27. HMBC NMR spectra for andrognathine E (**5**) (600 MHz, d_6-DMSO). 64 scans, NUS50 and 256 increments

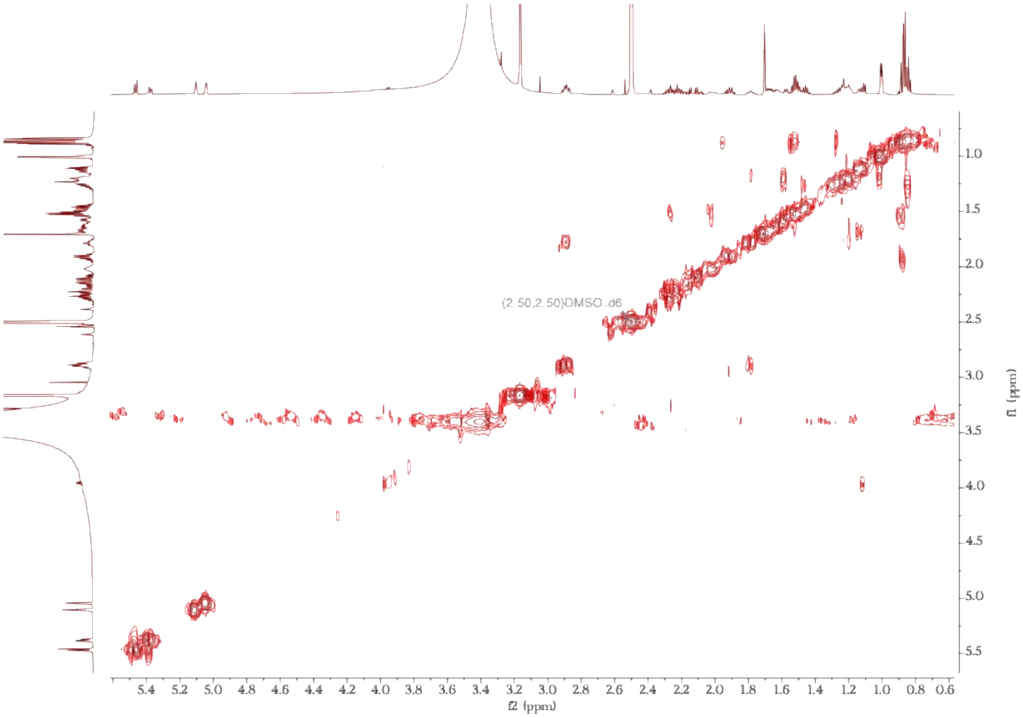

Figure S28. COSY NMR spectra for andrognathine E (**5**) (600 MHz, d_6-DMSO). 128 scans, NUS50 and 128 increments

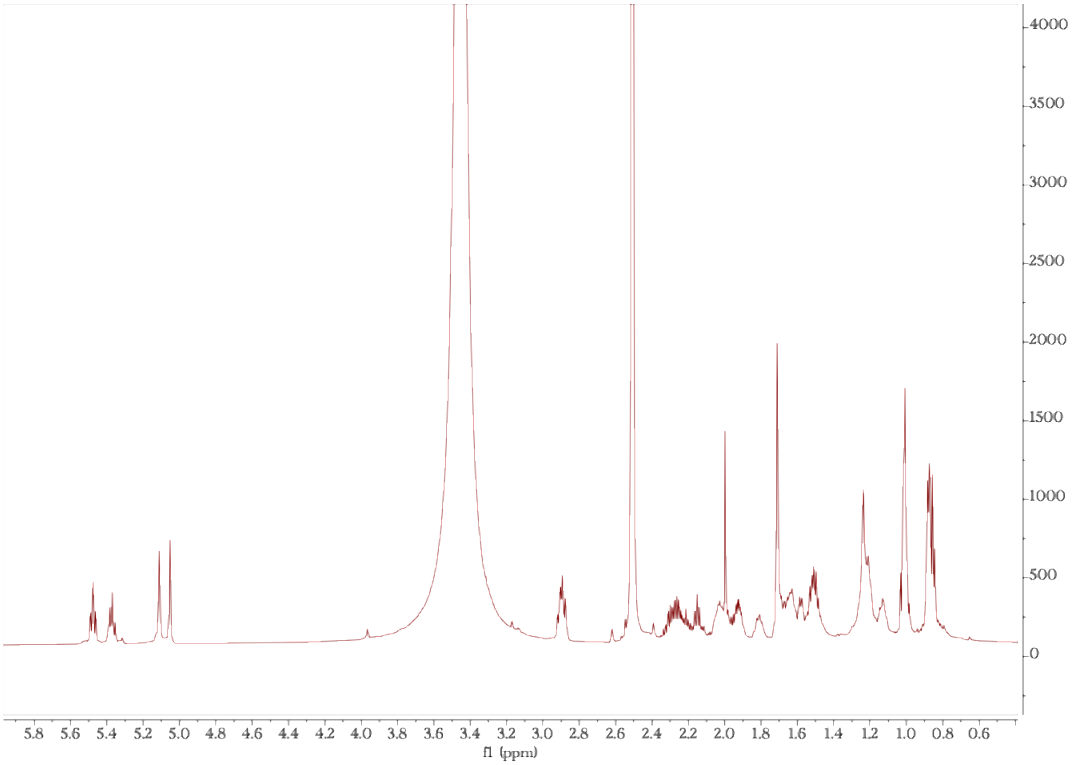

Figure S29. ^{1}H NMR spectra for andrognathine F (**6**) (600 MHz, d_6-DMSO). 128 scans

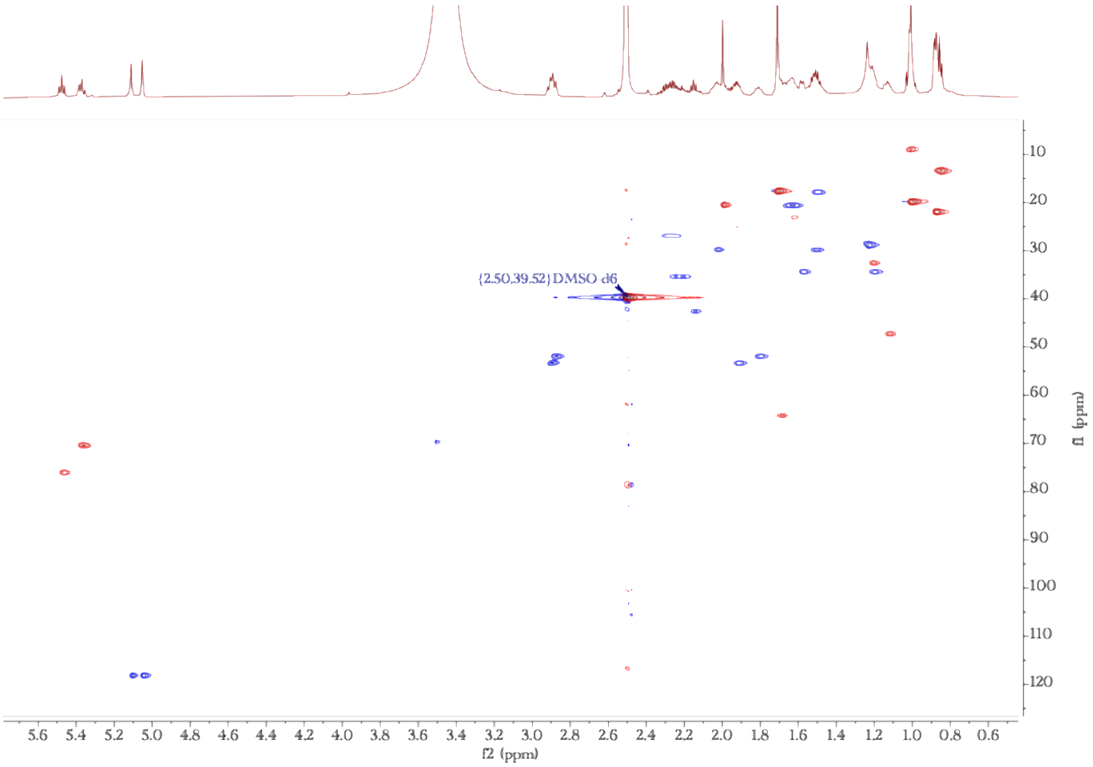

Figure S30. gHSQC NMR spectra for andrognathine F (**6**) (600 MHz, d_6-DMSO). 64 scans, NUS25 and 400 increments

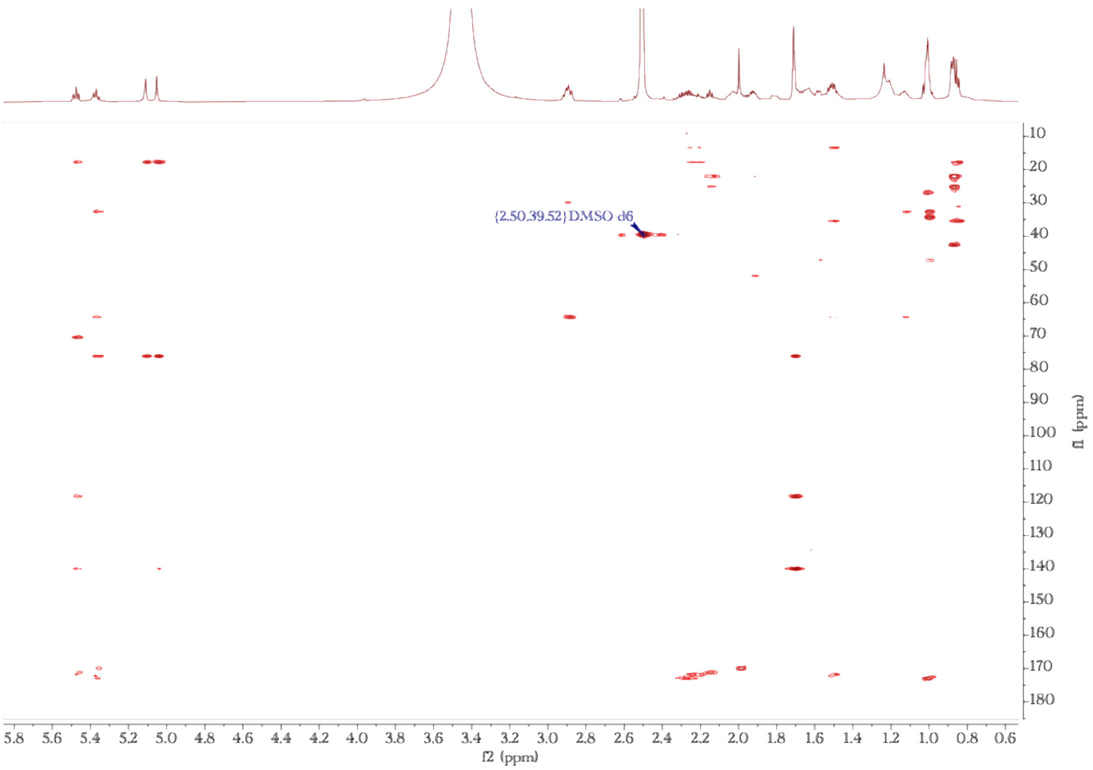

Figure S31. HMBC NMR spectra for andrognathine F (**6**) (600 MHz, d_6-DMSO). 32 scans, NUS50 and 512 increments

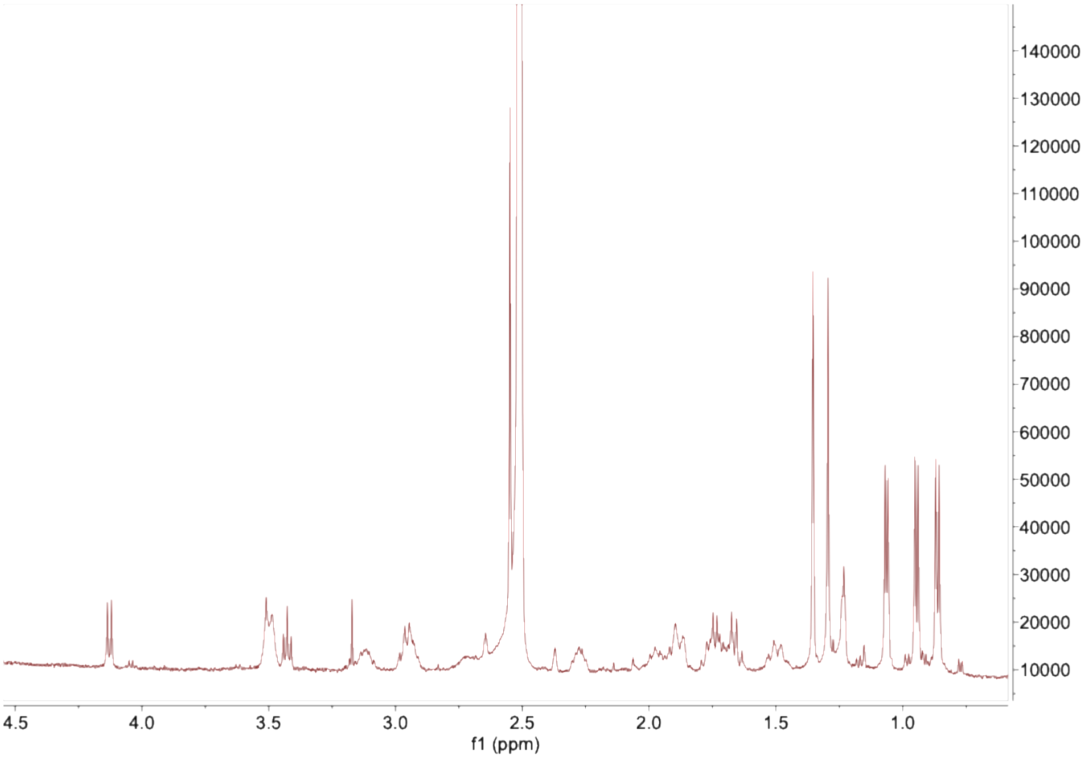

Figure S32. ^{1}H NMR spectra for andrognathine acetonide (**19**) (600 MHz, d_6-DMSO with fuming TFA). 128 scans

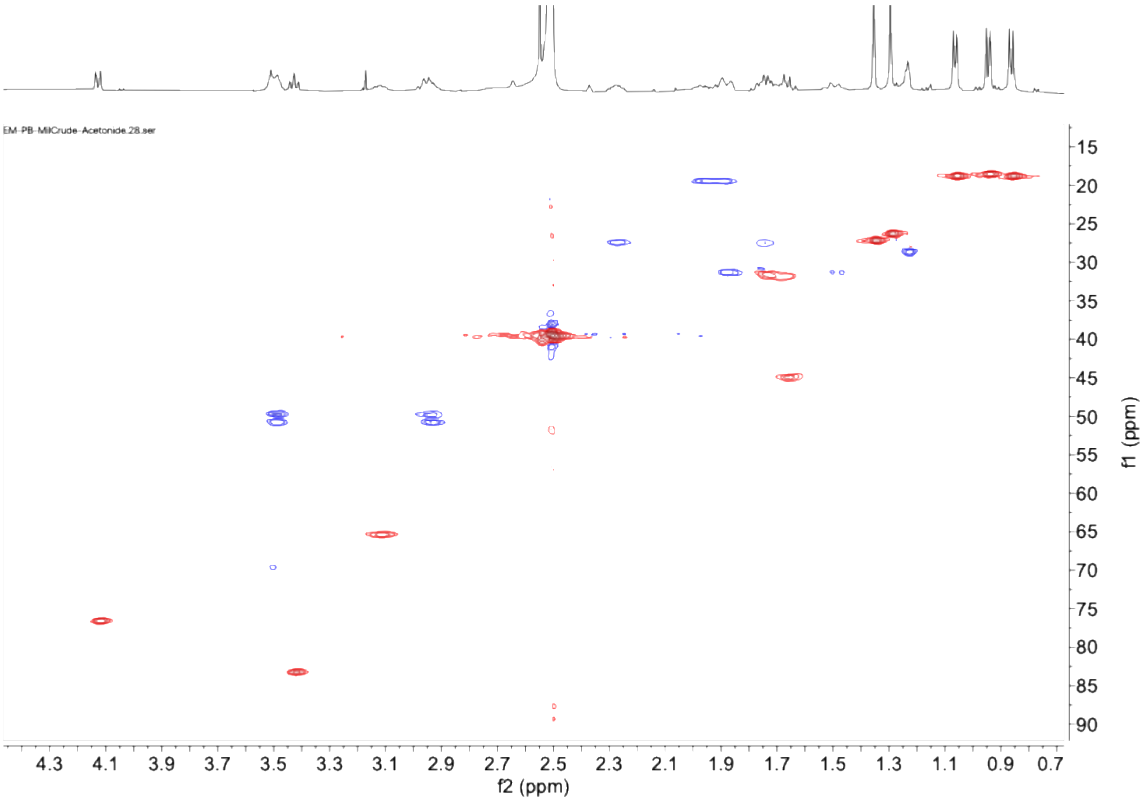

Figure S33. gHSQC NMR spectra for andrognathine acetonide (**19**) (600 MHz, d_6-DMSO with fuming TFA). 256 scans, NUS25 and 400 increments

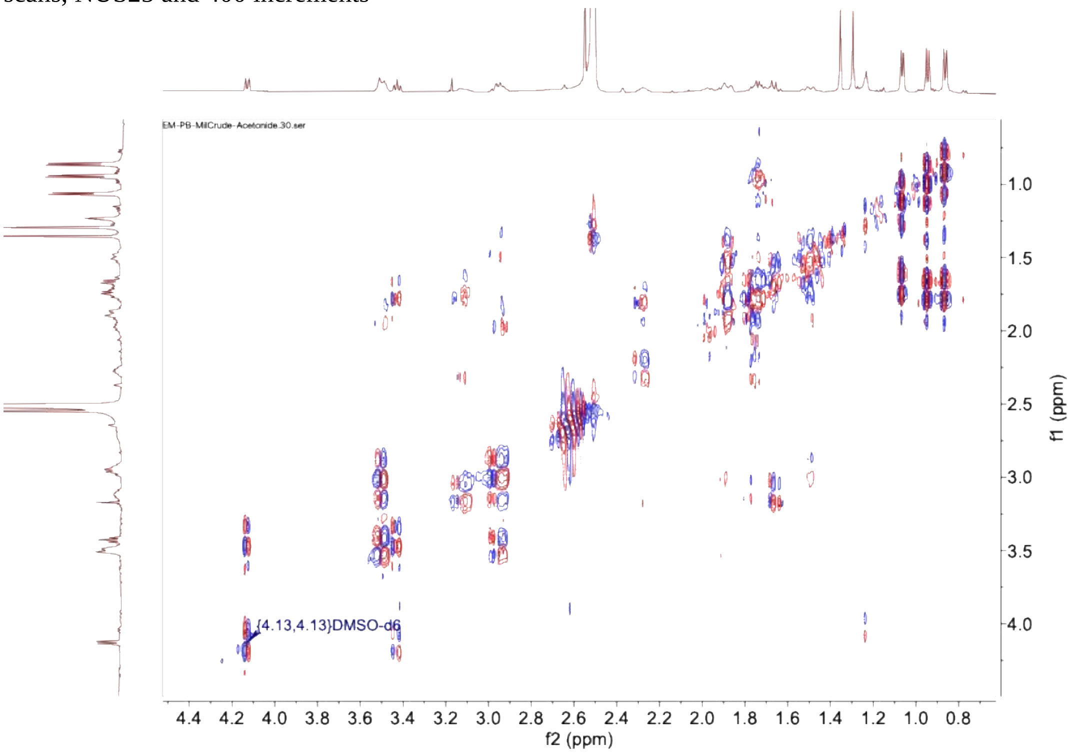

Figure S34. dqfCOSY NMR spectra for andrognathine acetonide (**19**) (600 MHz, d_6-DMSO with fuming TFA). 128 scans, NUS50 and 256 increments

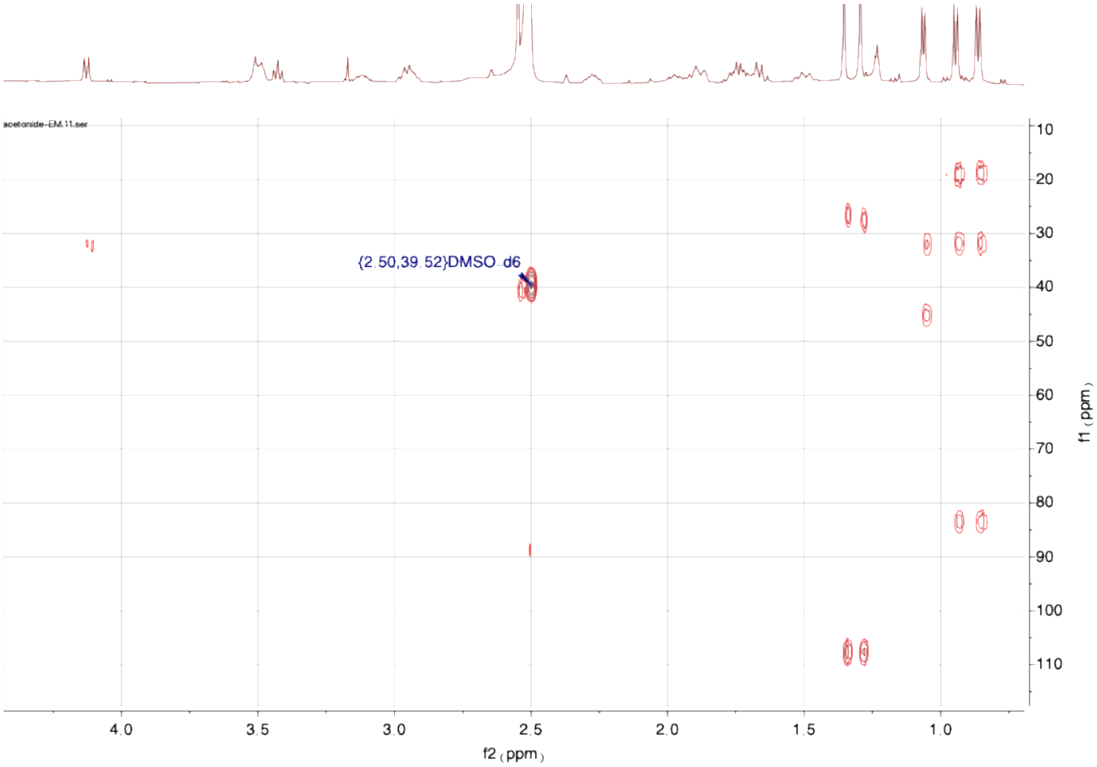

Figure S35. HMBC NMR spectra for andrognathine acetonide (**19**) (600 MHz, d_6-DMSO with fuming TFA). 128 scans, NUS50 and 256 increments

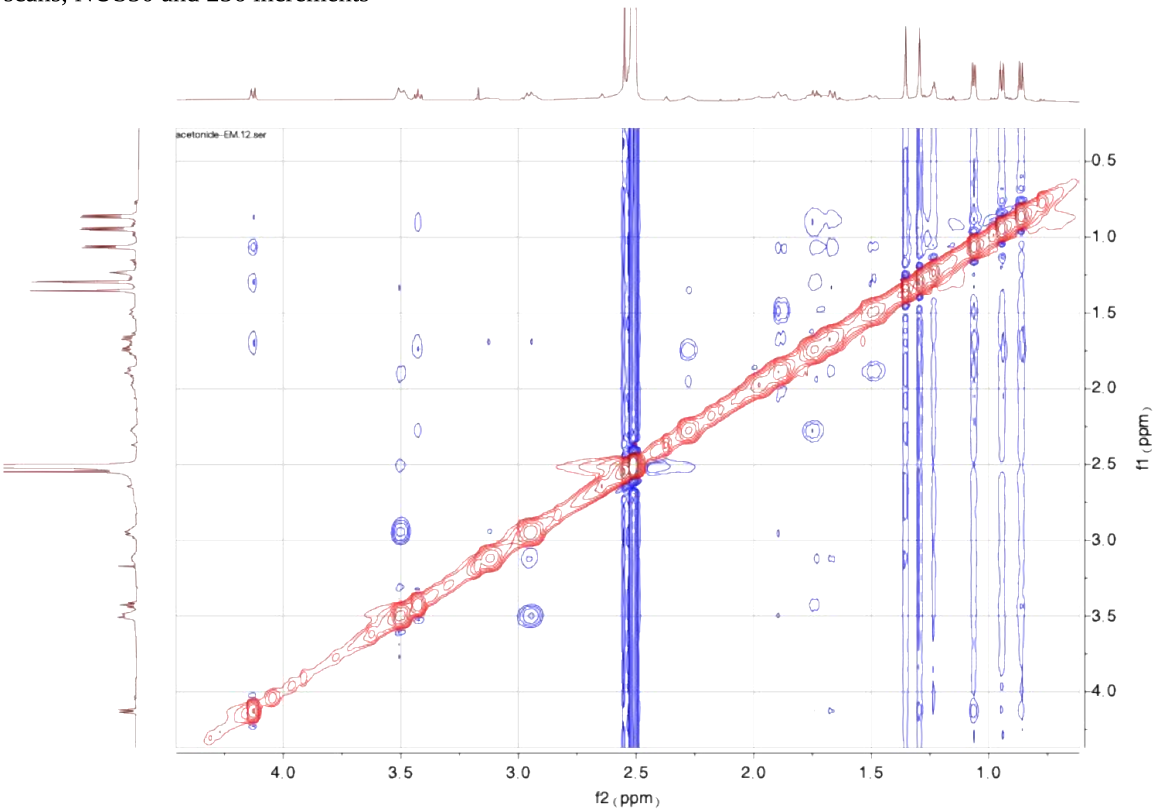

Figure S36. easyROESY NMR spectra for andrognathine acetonide (**19**) (600 MHz, d_6-DMSO with fuming TFA). 128 scans, NUS50 and 256 increments

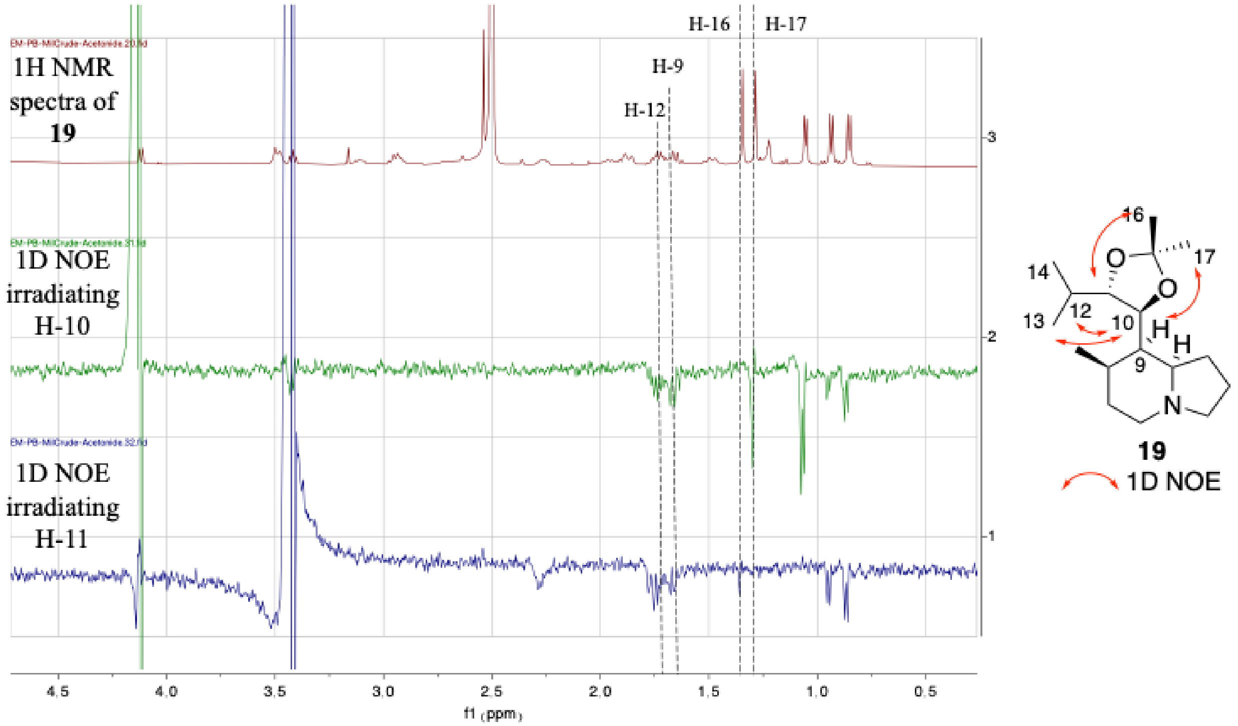

Figure S37. 1D NOE spectrum of andrognathine acetonide (**19**) (600 MHz, d_6-DMSO with fuming TFA). Top (red): 1H NMR spectra, middle (green): H-10 (4.10 ppm) was irradiated, and bottom (blue): H-11 (3.41 ppm) was irradiated. 128 scans

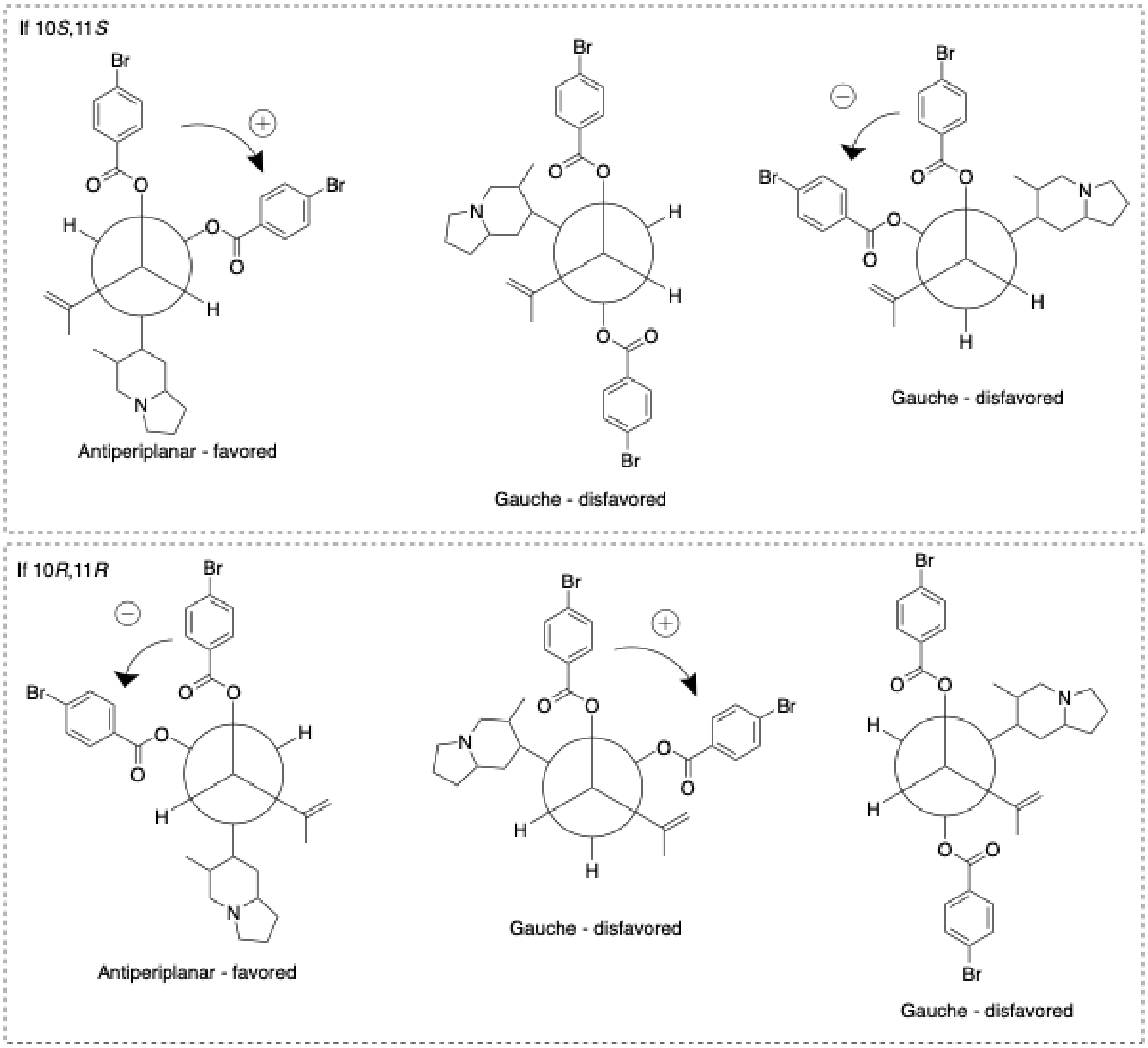

Figure S38. Newman projections for the conformers for the two *anti*-configurations. The 10*S*,11*S* absolute configuration should have a net positive split Cotton effect due to the stability of the antiperiplanar conformer. Conversely, the 10*R*,11*R* absolute configuration should have a net negative split Cotton effect.

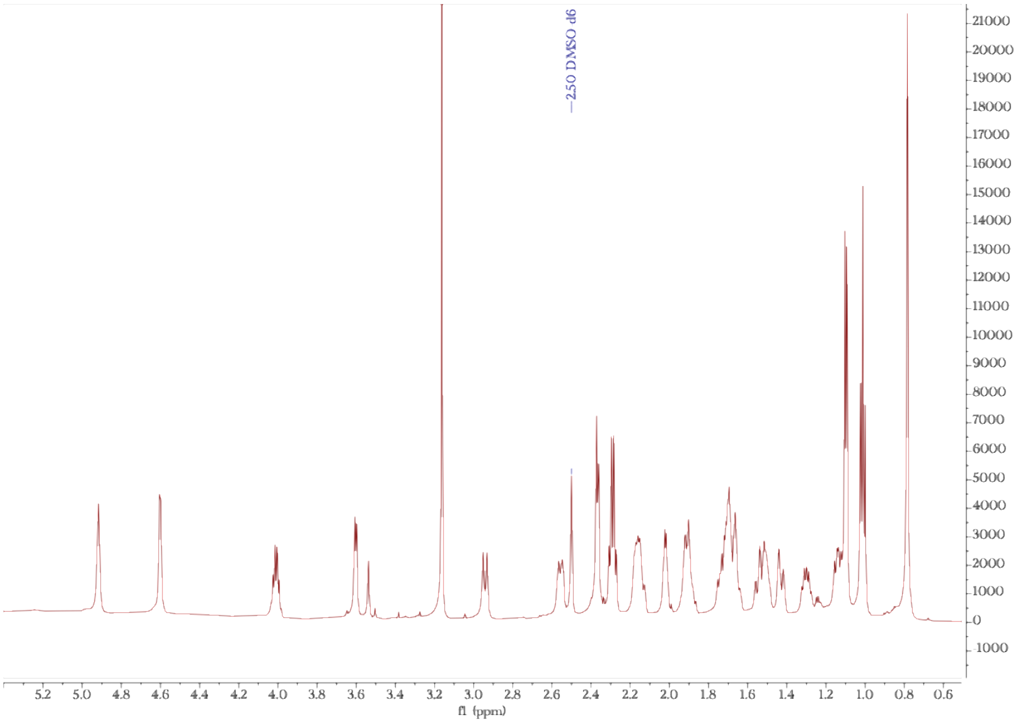

Figure S39. ^{1}H NMR spectra for andrognathanol A (**13**) (600 MHz, d_6-DMSO) 128 scans.

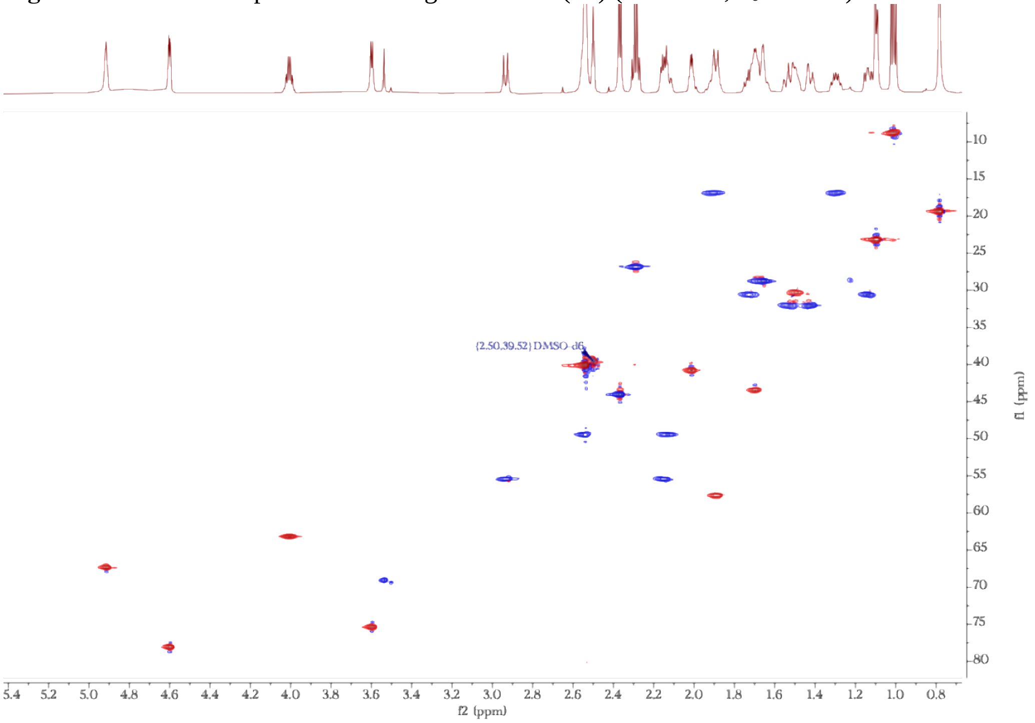

Figure S40. gHSQC NMR spectra for andrognathanol A (**13**) (600 MHz, d6-DMSO). 64 scans, NUS25 and 400 increments.

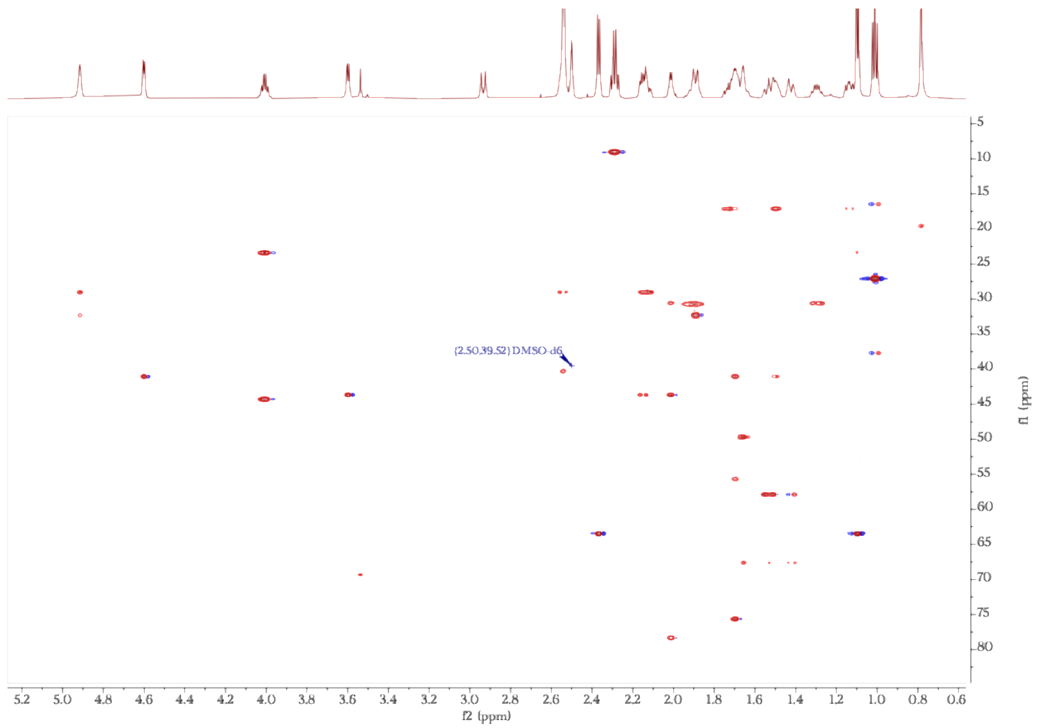

Figure S41. H2BC NMR spectra for andrognathanol A (**13**) (600 MHz, d_6-DMSO). 64 scans, NUS50 and 400 increments.

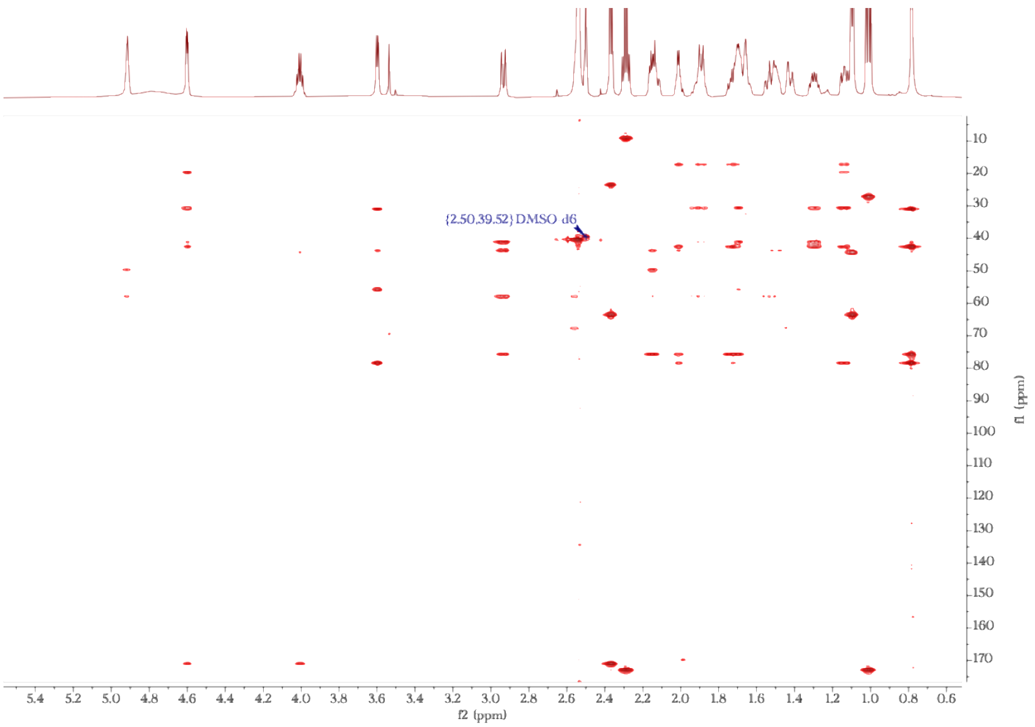

Figure S42. HMBC NMR spectra for andrognathanol A (**13**) (600 MHz, d_6-DMSO). 64 scans, NUS50 and 292 increments.

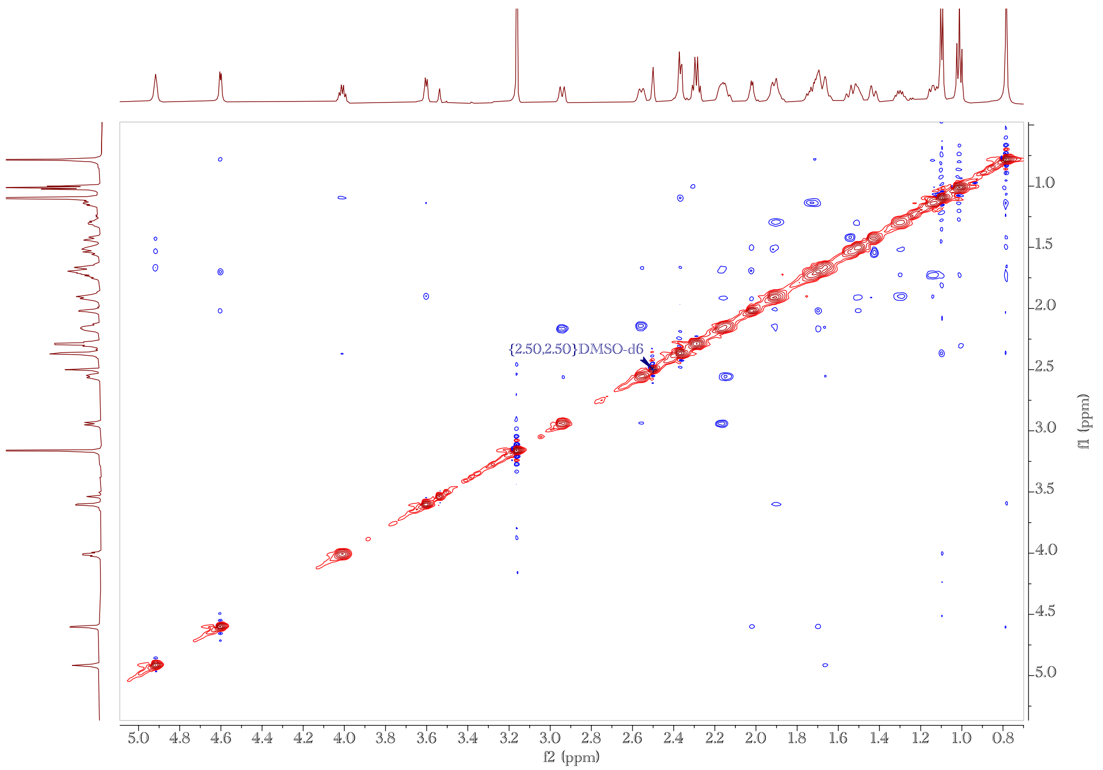

Figure S43. easyROESY NMR spectra for andrognathanol A (**13**) (600 MHz, d_6-DMSO). 64 scans, NUS50, and 356 increments

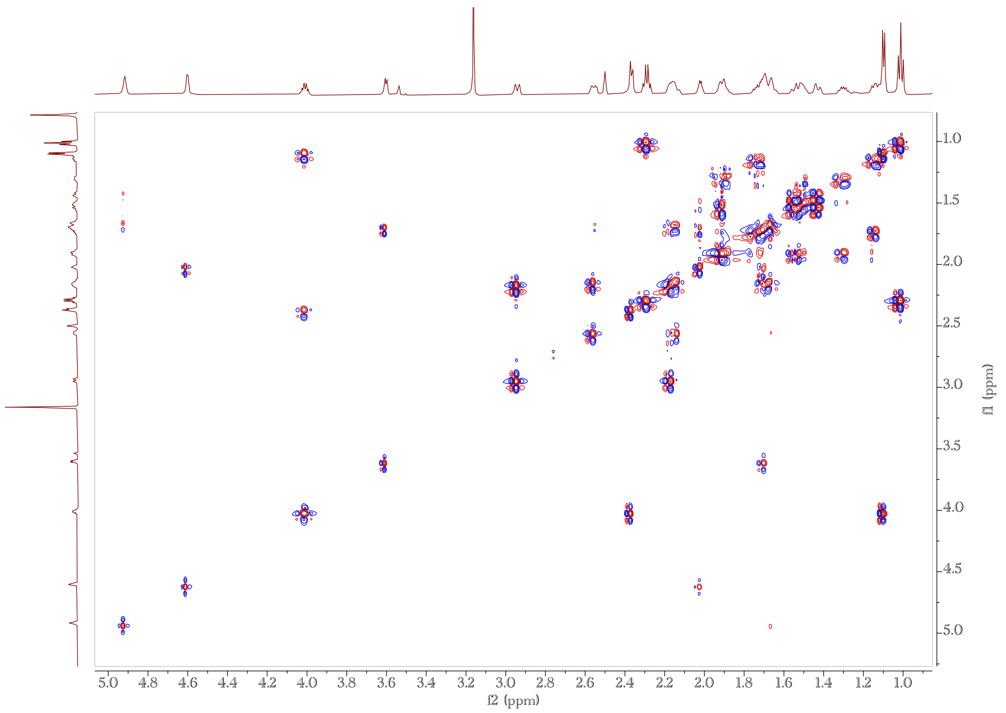

Figure S44. dqfCOSY NMR spectra for andrognathanol A (**13**) (600 MHz, d_6-DMSO). 48 scans, NUS50 and 256 increments

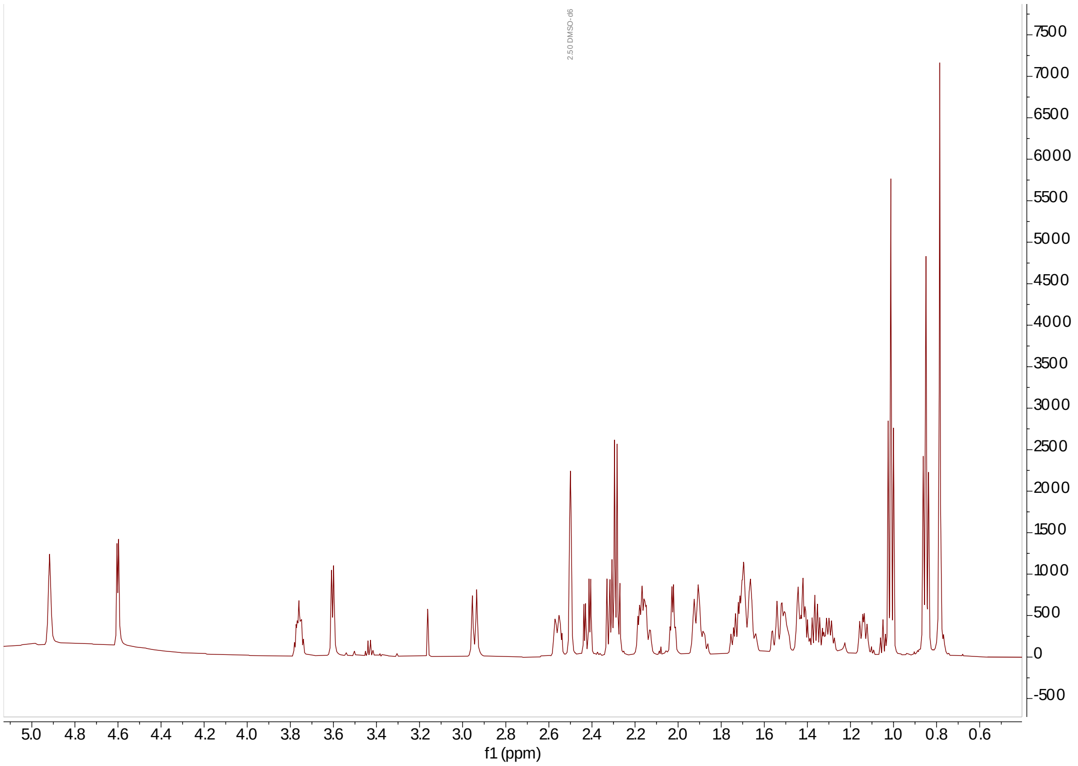

Figure S45. ^{1}H NMR spectra for andrognathanol B (**14**) (600 MHz, d_6-DMSO). 128 scans

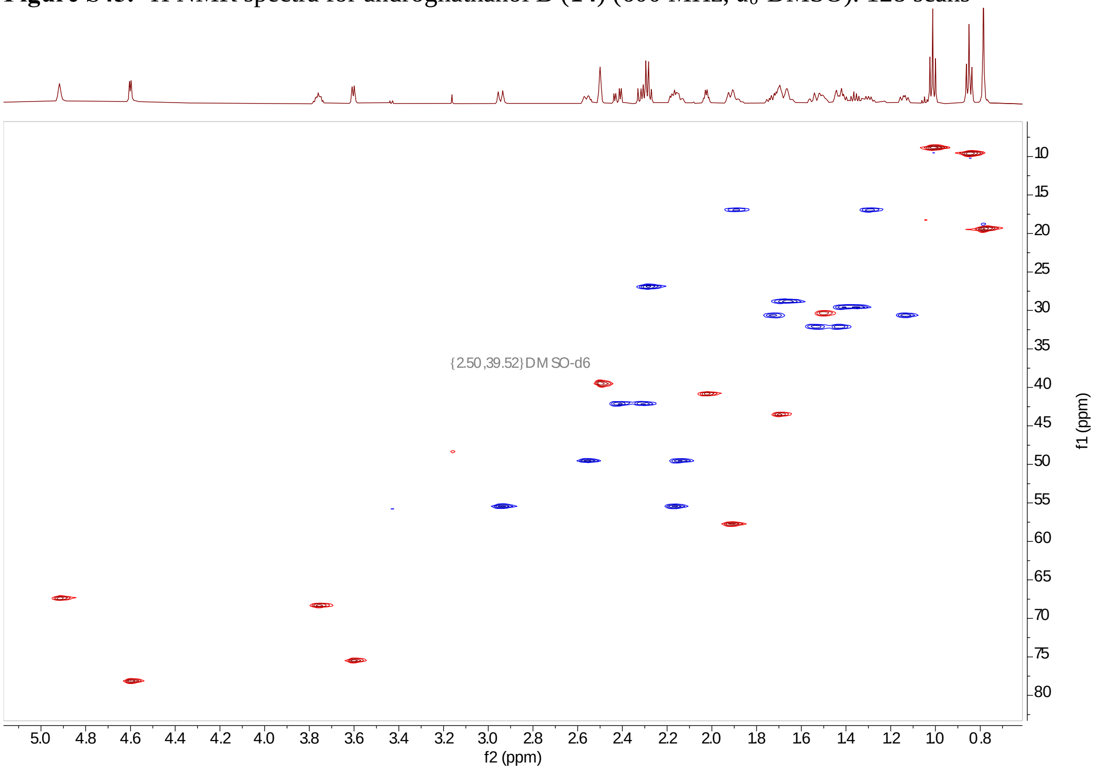

Figure S46. gHSQC NMR spectra for andrognathanol B (**14**) (600 MHz, d_6-DMSO). 32 scans, NUS25, and 400 increments.

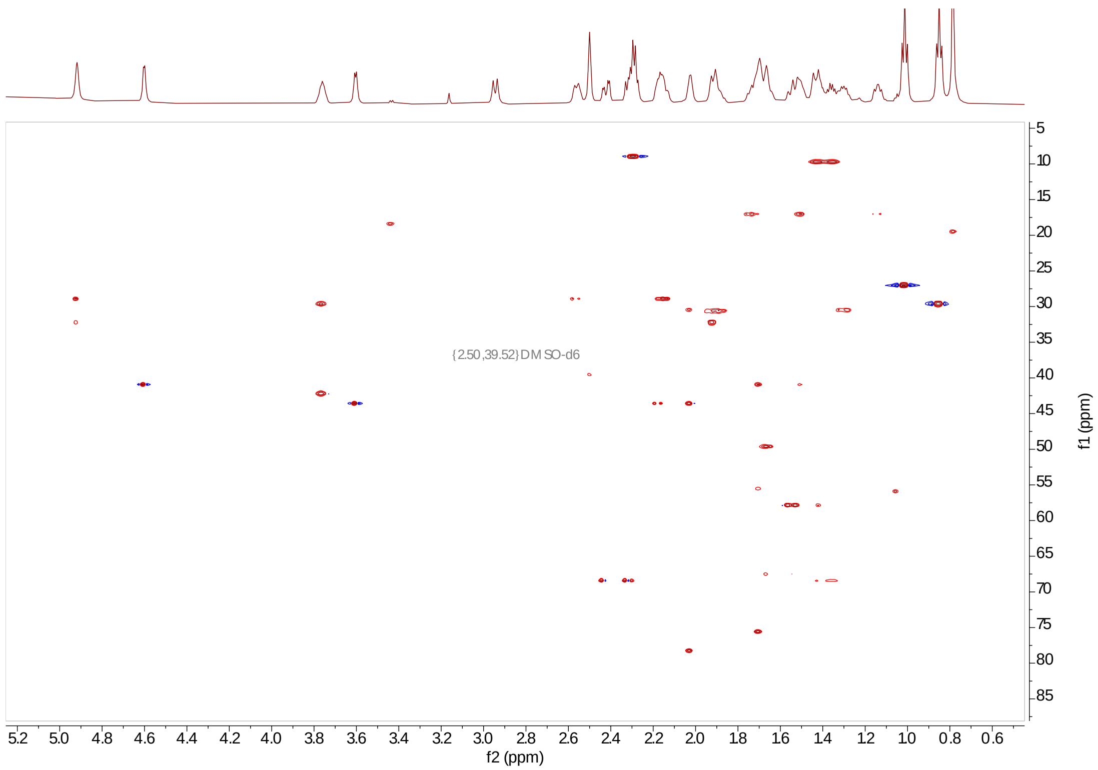

Figure S47. H2BC NMR spectra for andrognathanol B (**14**) (600 MHz, d_6-DMSO). 32 scans, NUS50, and 400 increments

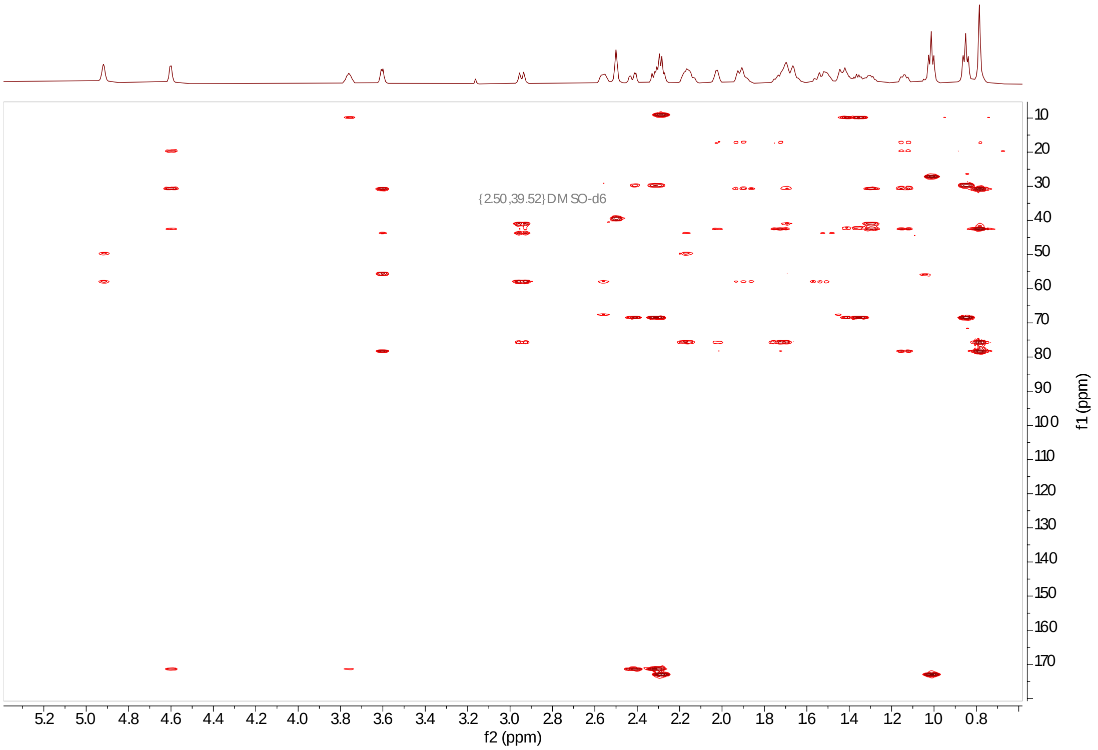

Figure S48. HMBC NMR spectra for andrognathanol B (**14**) (600 MHz, d_6-DMSO). 32 scans, NUS50, and 512 increments.

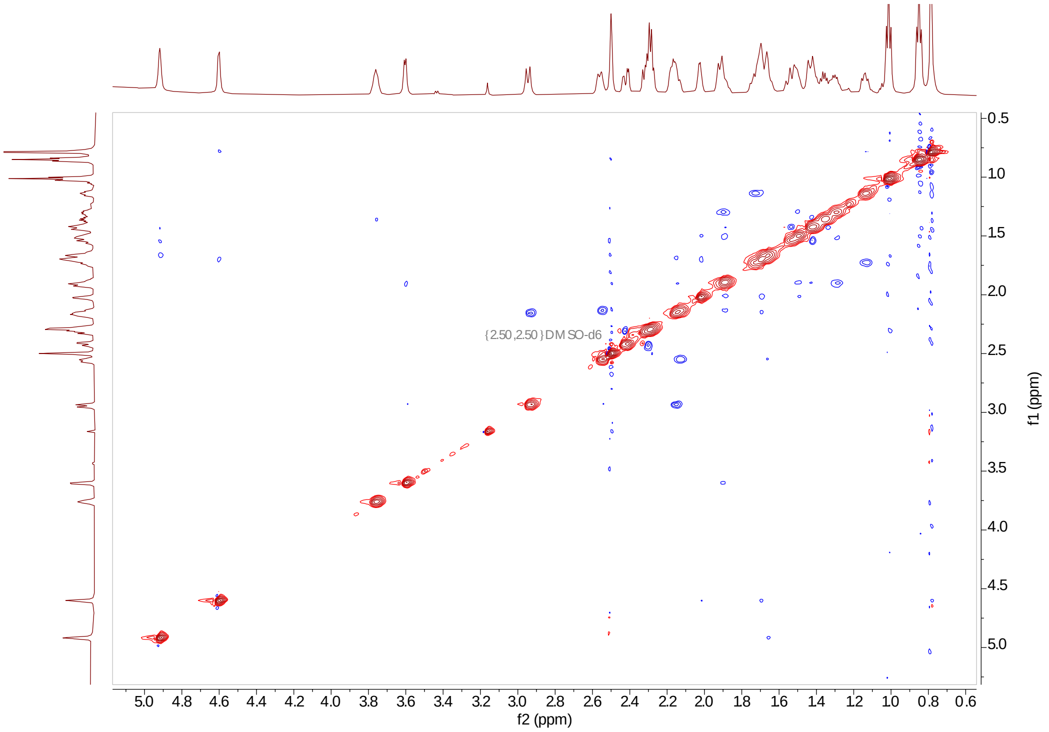

Figure S49. easyROESY NMR spectra for andrognathanol B (**14**) (600 MHz, d_6-DMSO). 32 scans, NUS50, and 400 increments.

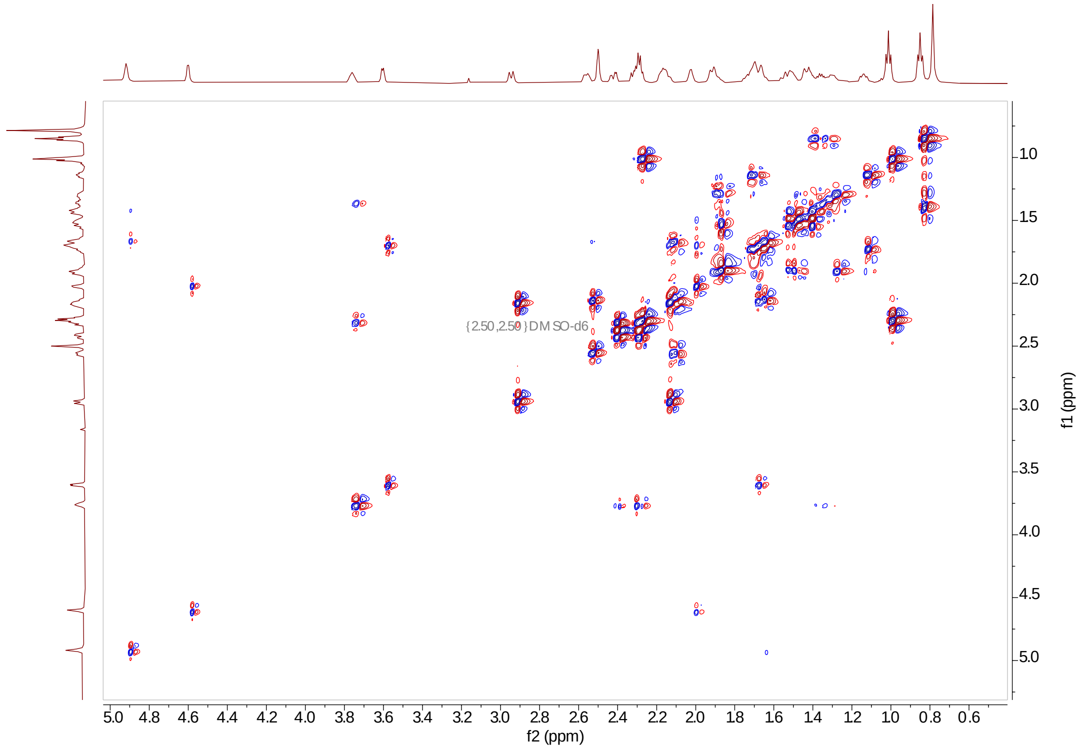

Figure S50. dqfCOSY NMR spectra for andrognathanol B (**14**) (600 MHz, d_6-DMSO). 16 scans, NUS50, and 256 increments.

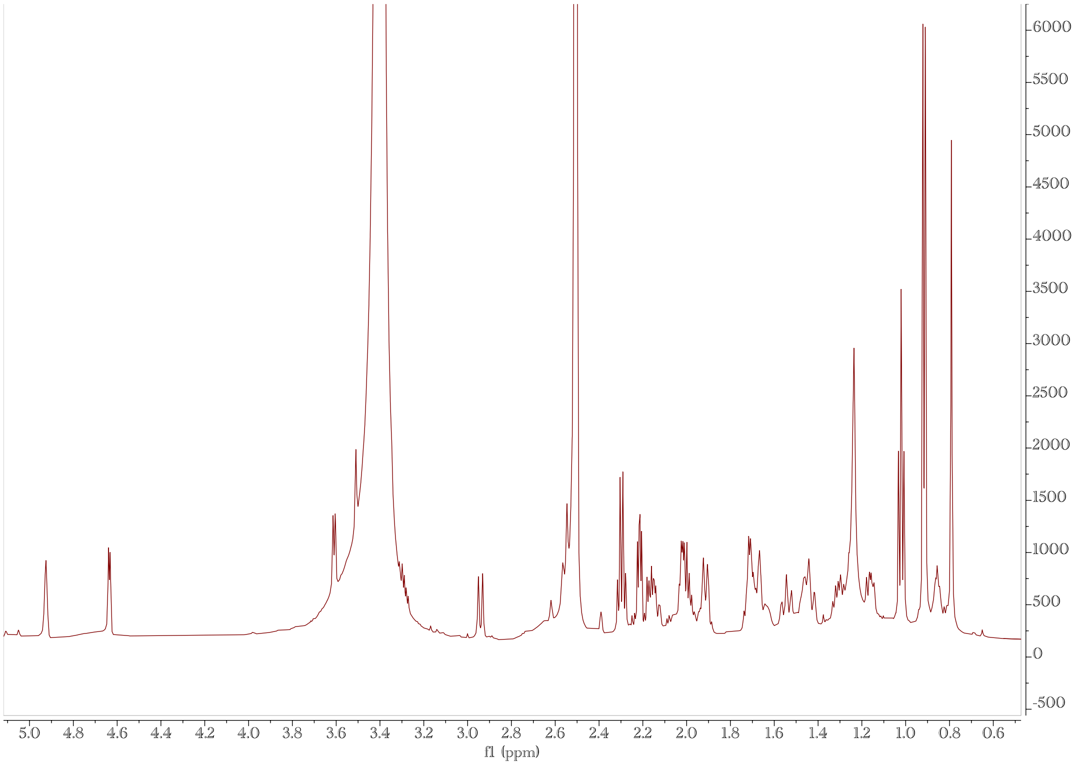

Figure S51. ^{1}H NMR spectra for andrognathanol C (**15**) (600 MHz, d_6-DMSO). 256 scans

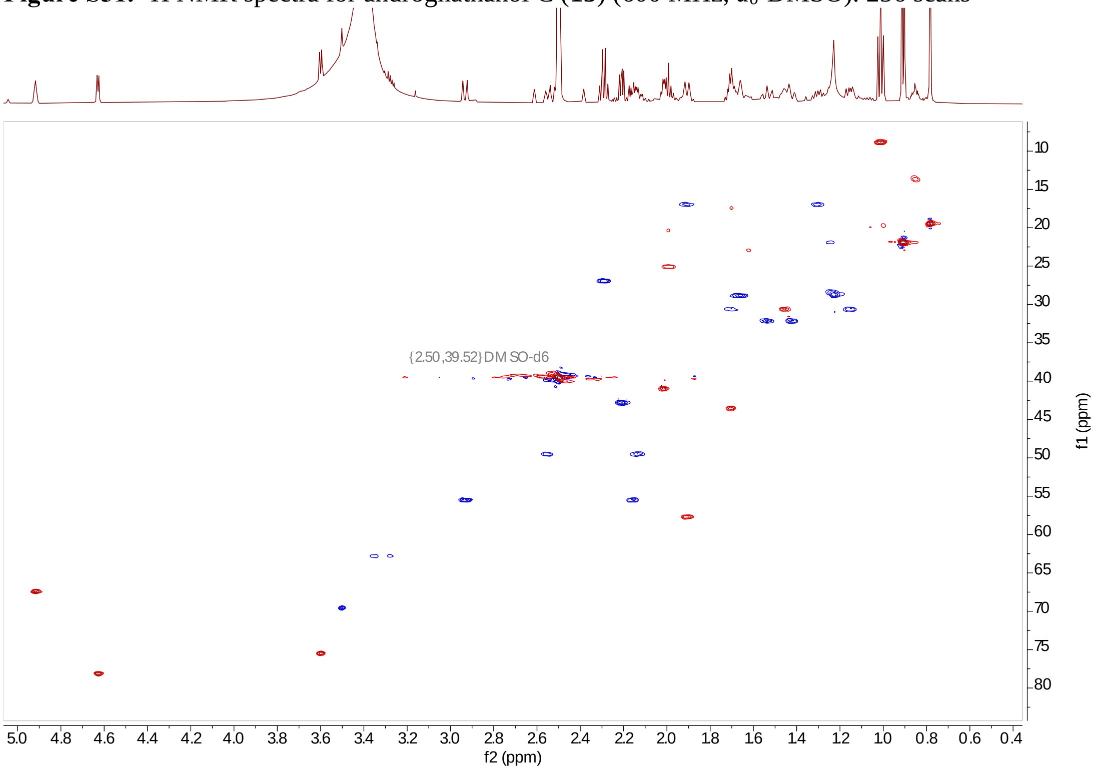

Figure S52. gHSQC NMR spectra for andrognathanol C (**15**) (600 MHz, d_6-DMSO). 64 scans, NUS25, and 400 increments.

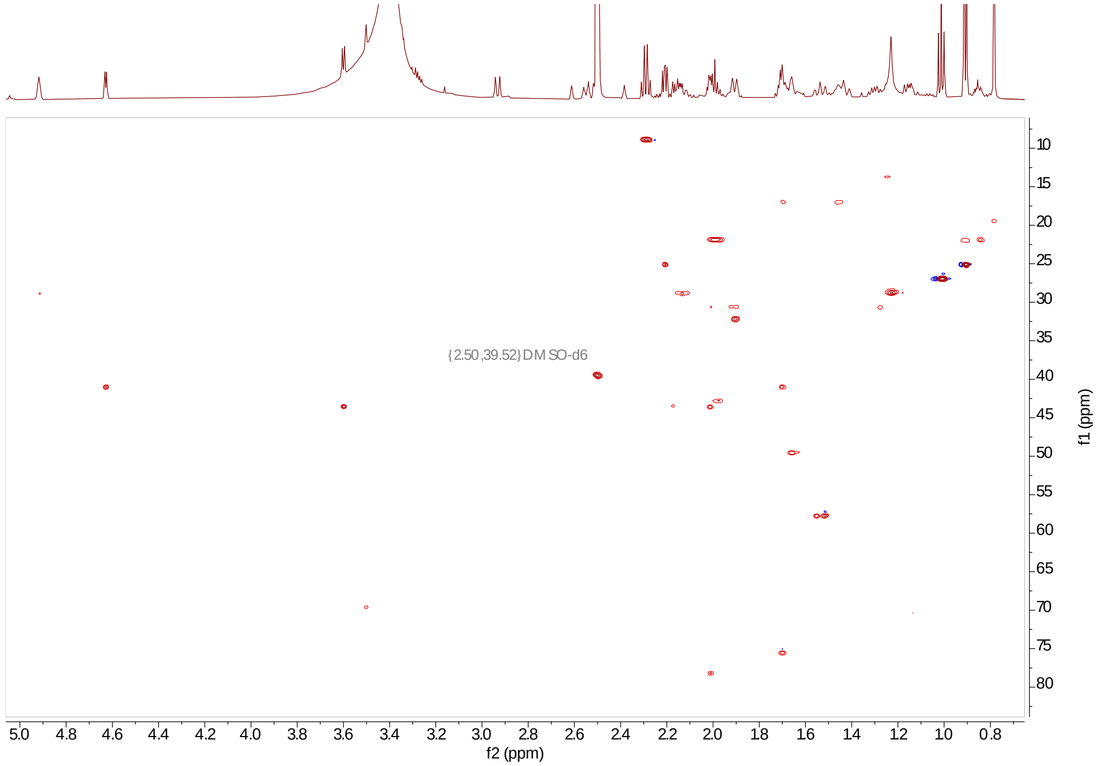

Figure S53. H2BC NMR spectra for andrognathanol C (**15**) (600 MHz, d_6-DMSO). 64 scans, NUS50, and 400 increments.

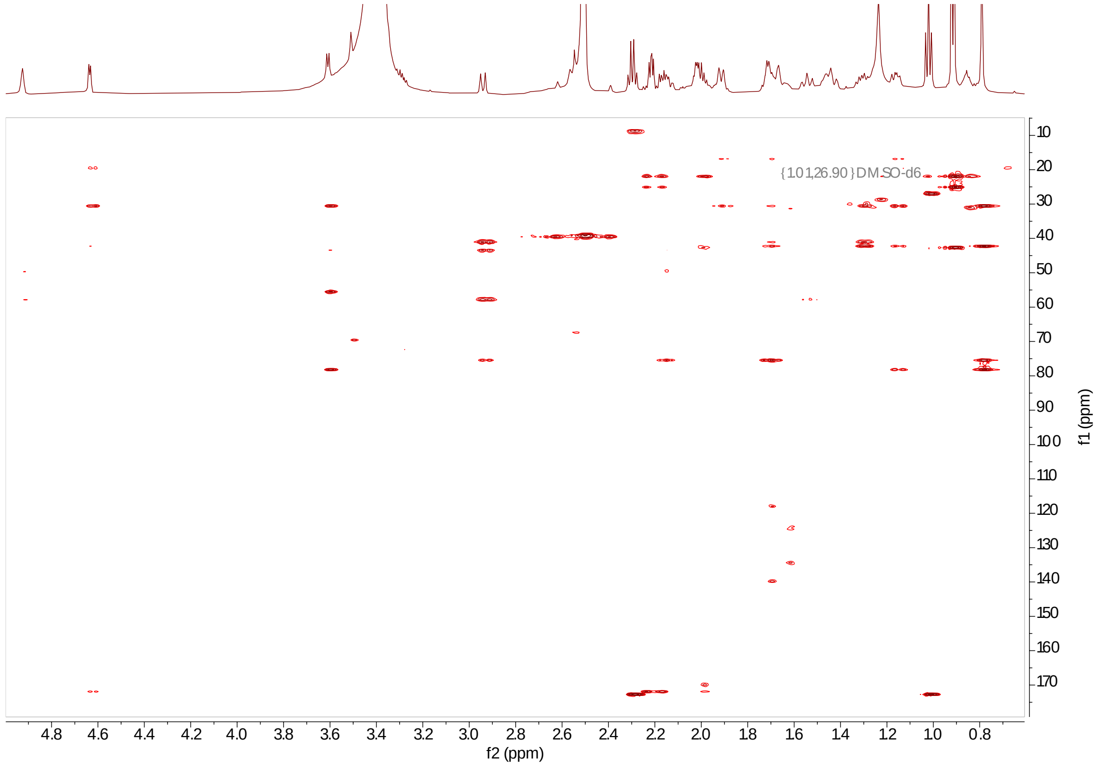

Figure S54. HMBC NMR spectra for andrognathanol C (**15**) (600 MHz, d_6-DMSO). 256 scans, NUS50, and 512 increments.

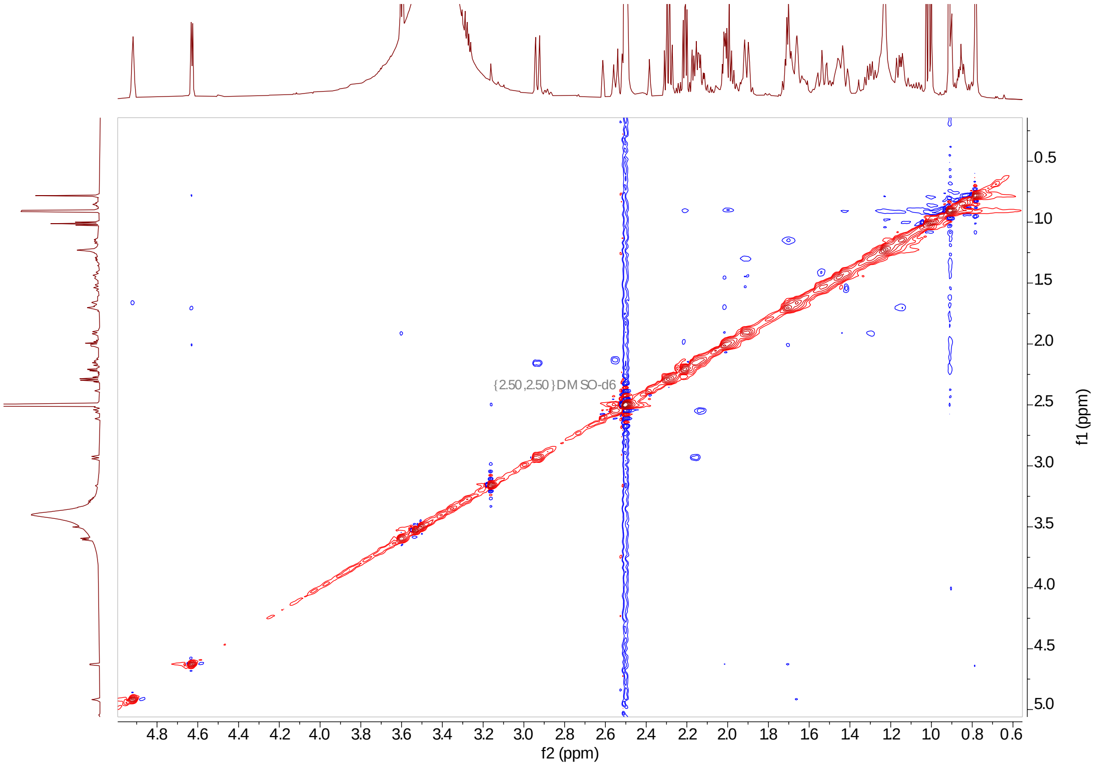

Figure S55. easyROESY NMR spectra for andrognathanol C (**15**) (600 MHz, d_6-DMSO). 64 scans, NUS50, and 400 increments.

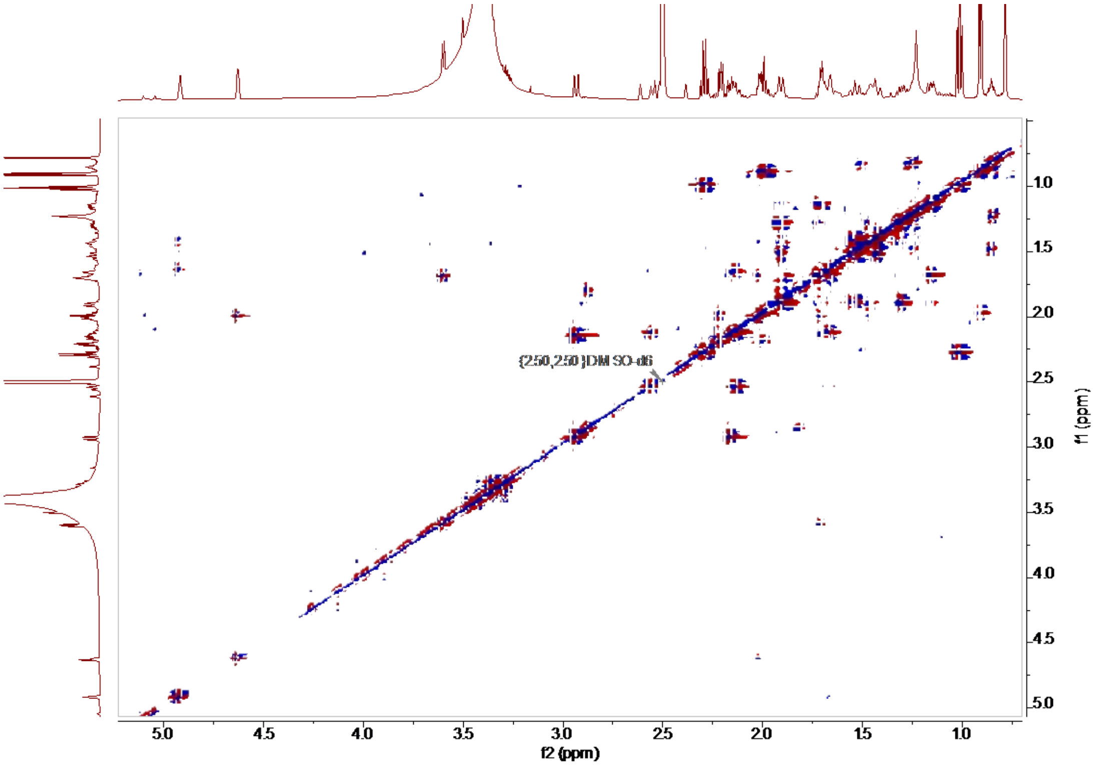

Figure S56. dqfCOSY NMR spectra for andrognathanol C (**15**) (600 MHz, d_6-DMSO). 64 scans, NUS50, and 400 increments.

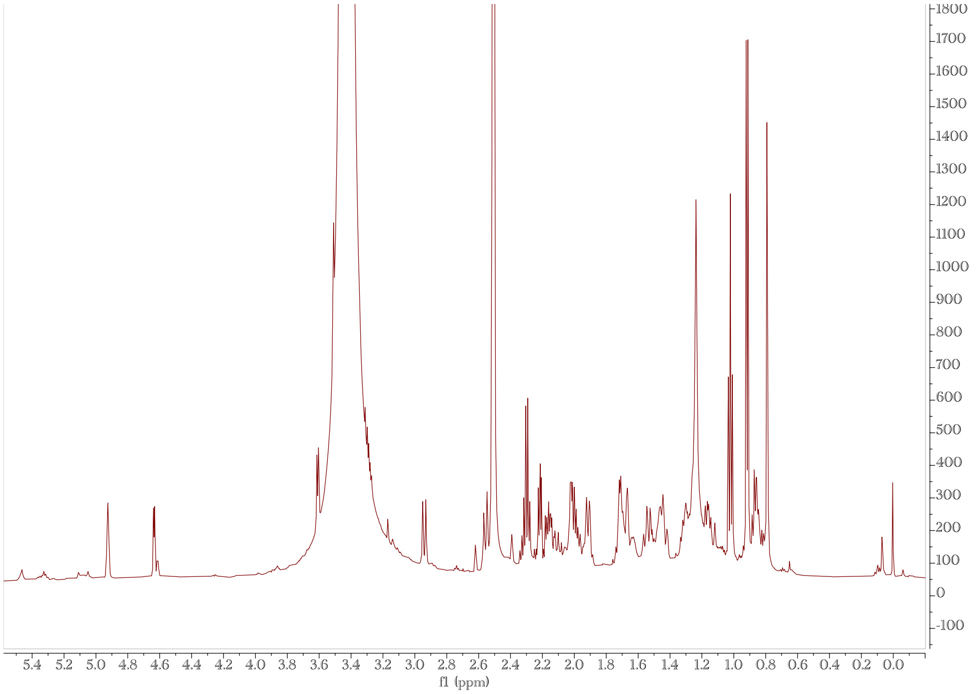

Figure S57. ^{1}H NMR spectra for andrognathanol D (**16**) (600 MHz, d_6-DMSO). 128 scans

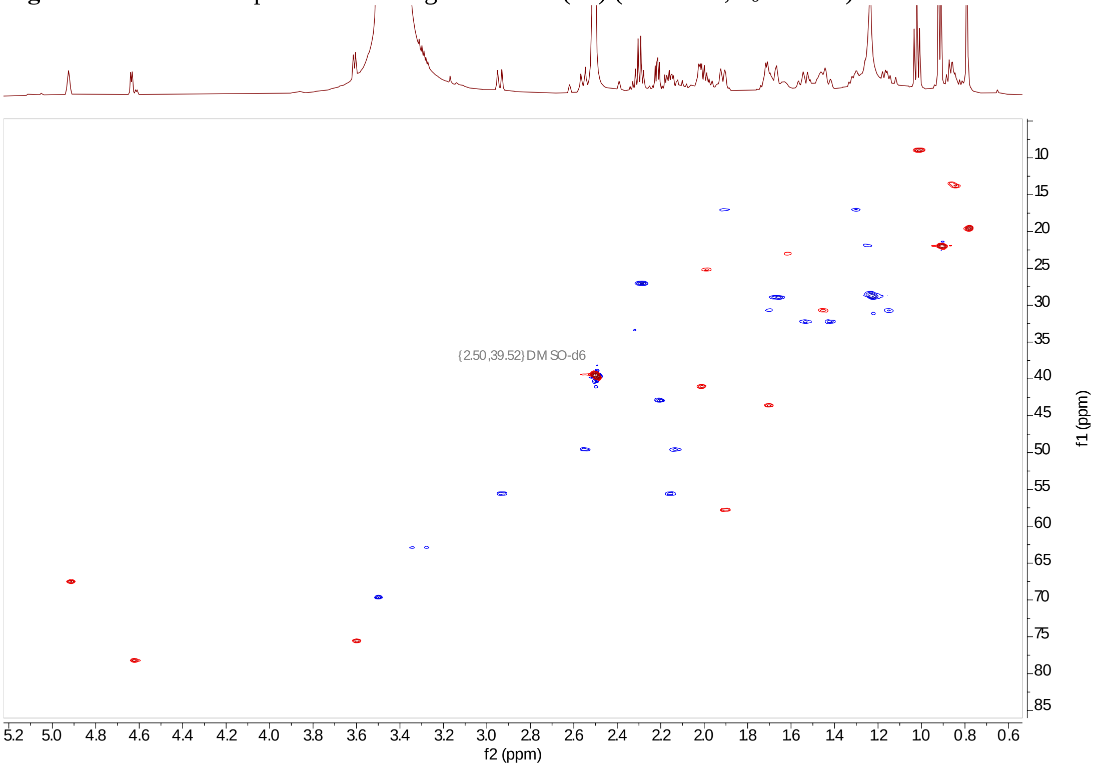

Figure S58. gHSQC NMR spectra for andrognathanol D (**16**) (600 MHz, d_6-DMSO). 64 scans, NUS25, and 400 increments.

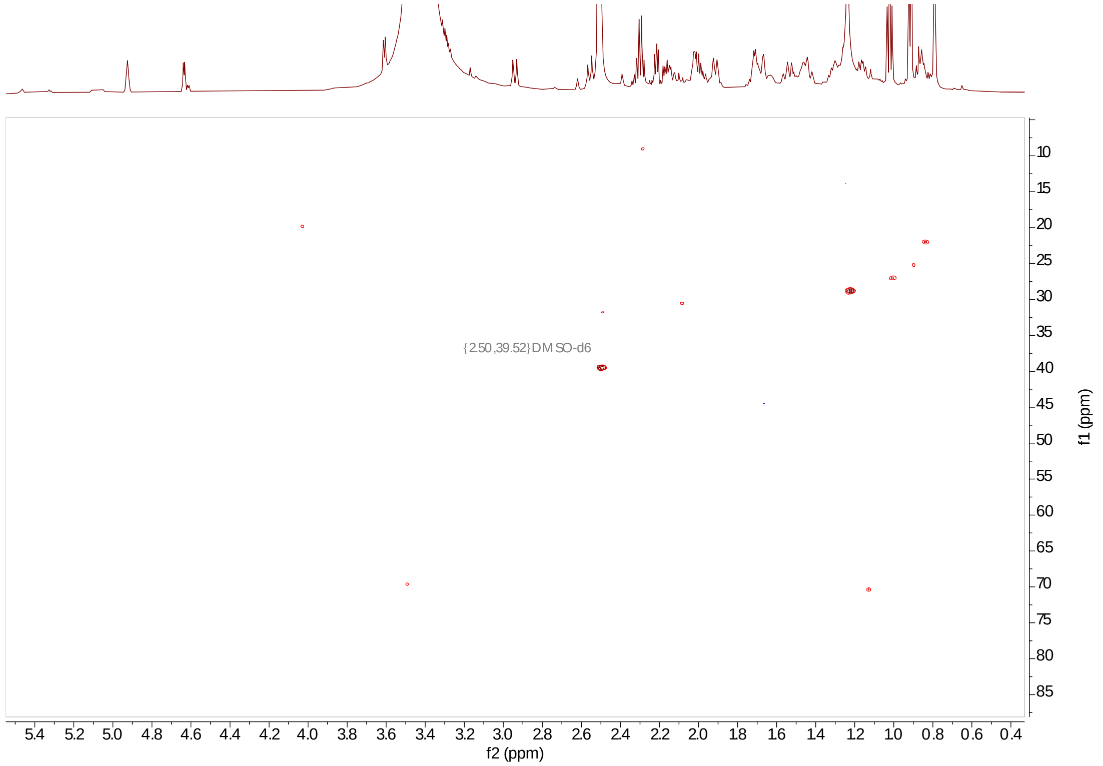

Figure S59. H2BC NMR spectra for andrognathanol D (**16**) (600 MHz, d_6-DMSO). 64 scans, NUS50, and 400 increments.

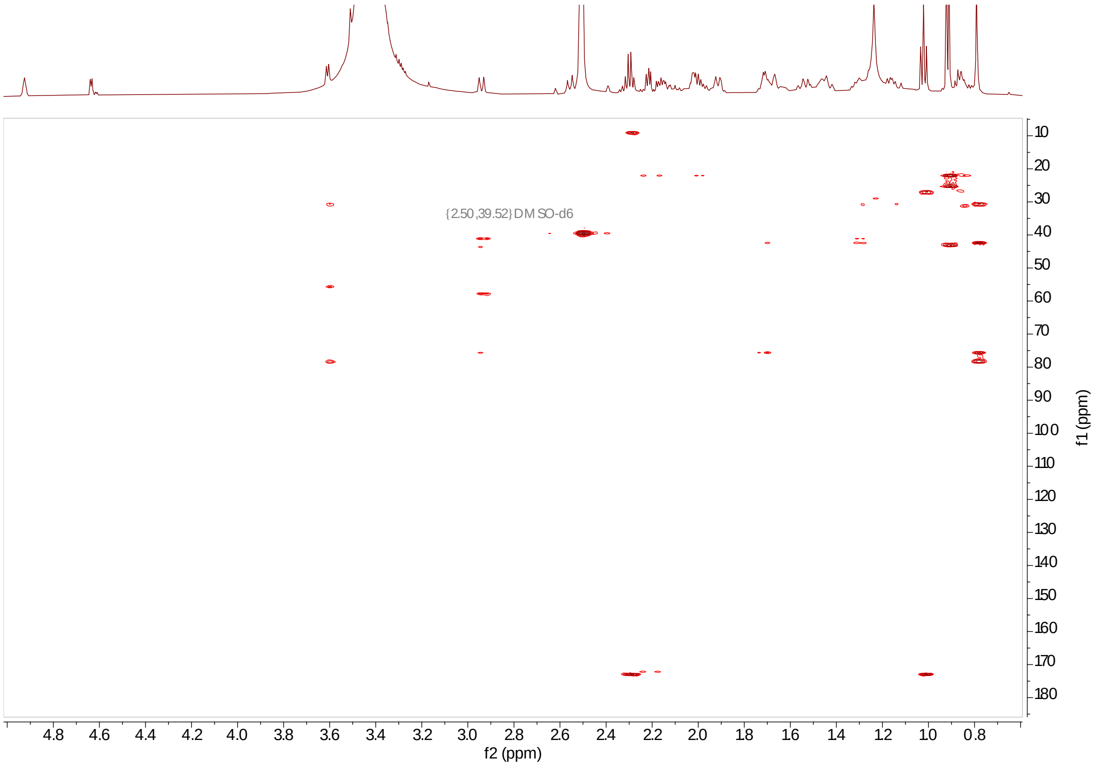

Figure S60. HMBC NMR spectra for andrognathanol D (**16**) (600MHz, d_6-DMSO). 32 scans, NUS50, and 512 increments.

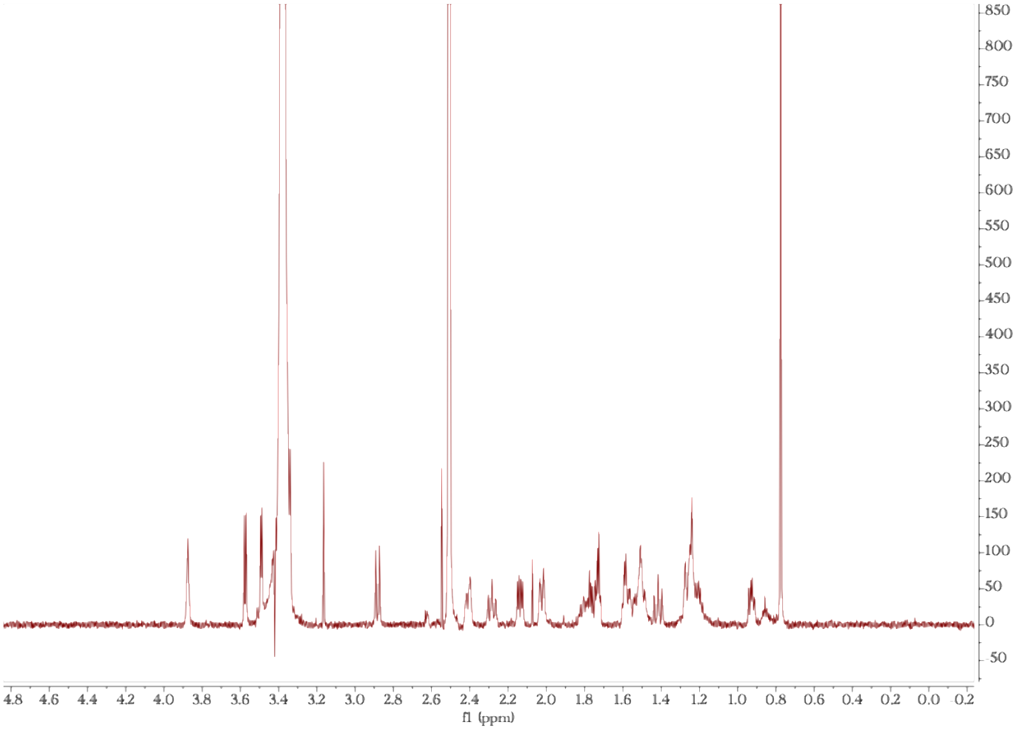

Figure S61. ^{1}H NMR spectra for andrognathanol hydrate (**21**) (600MHz, d_6-DMSO). 32 scans

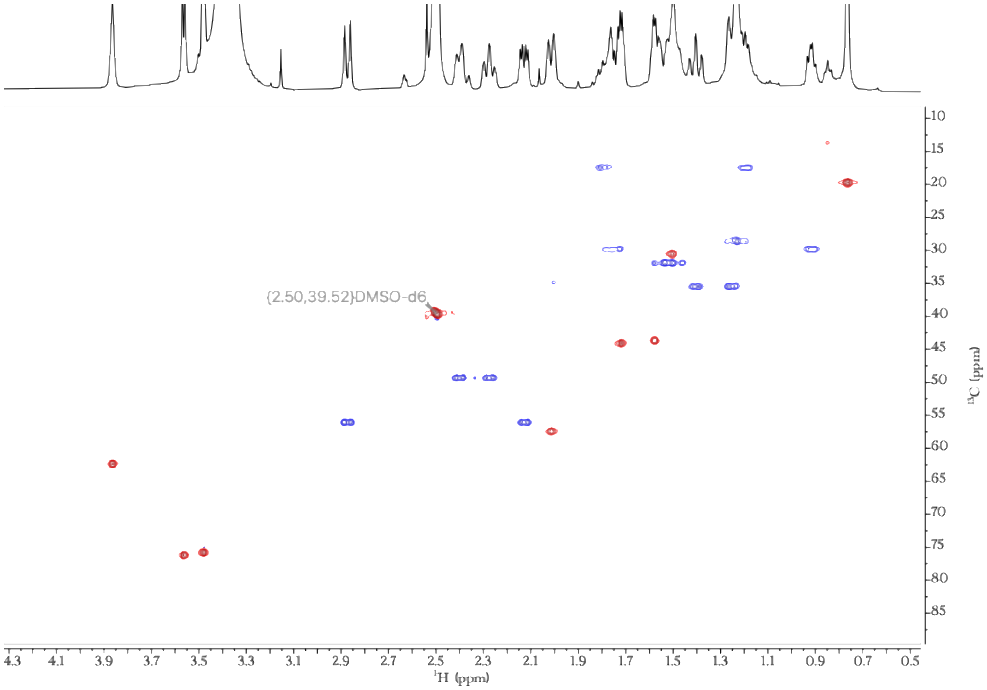

Figure S62. gHSQC NMR spectra for andrognathanol hydrate (**21**) (500MHz, d_6-DMSO). 128 scans, NUS50, and 256 increments.

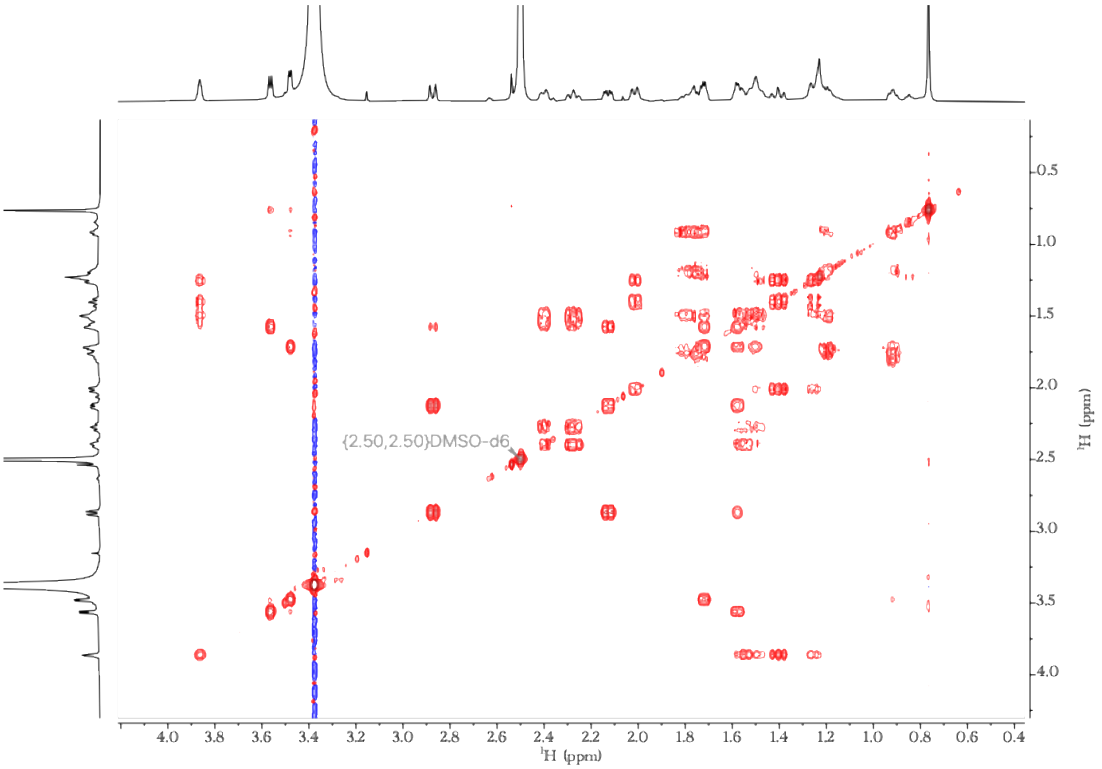

Figure S63. gCOSY spectra for andrognathanol hydrate (**21**) (500MHz, d_6-DMSO). 32 scans, NUS50, and 256 increments.

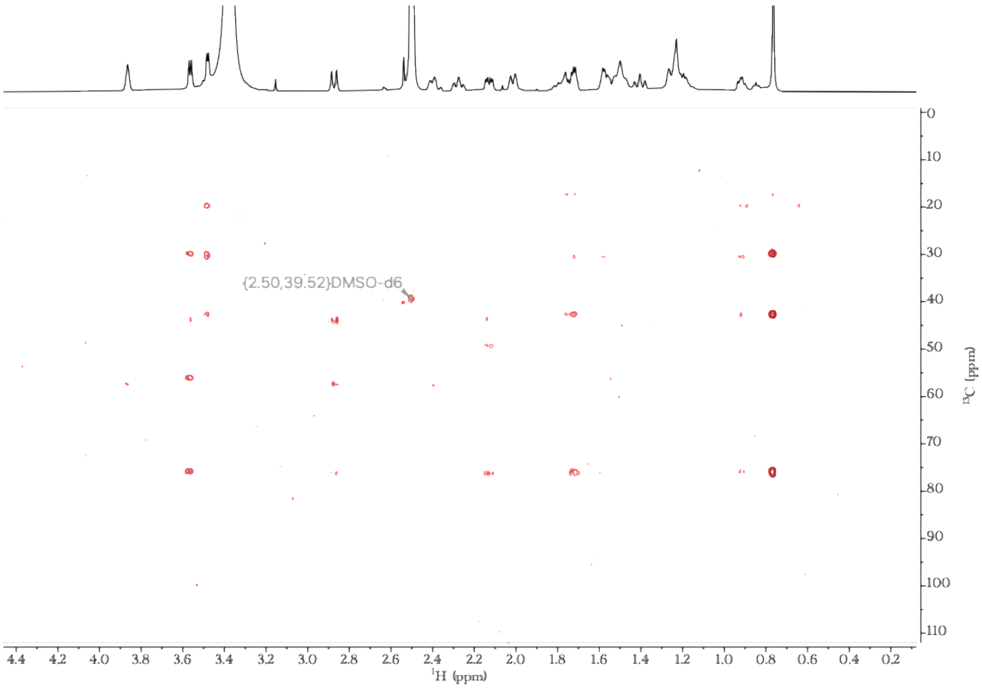

Figure S64. HMBC spectra for andrognathanol hydrate (**21**) (500MHz, d_6-DMSO). 128 scans, NUS50, and 256 increments.

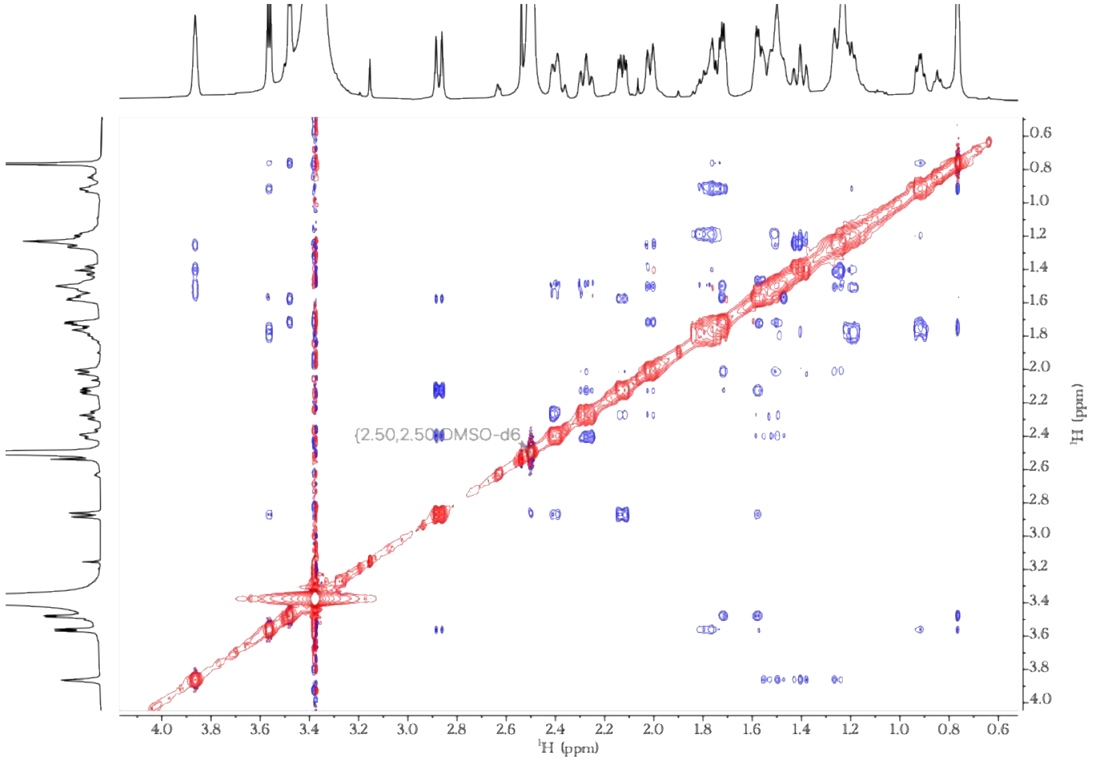

Figure S65. 2D easyROESY spectra for andrognathanol hydrate (**21**) (500MHz, d_6-DMSO). 32 scans, NUS50, and 256 increments.

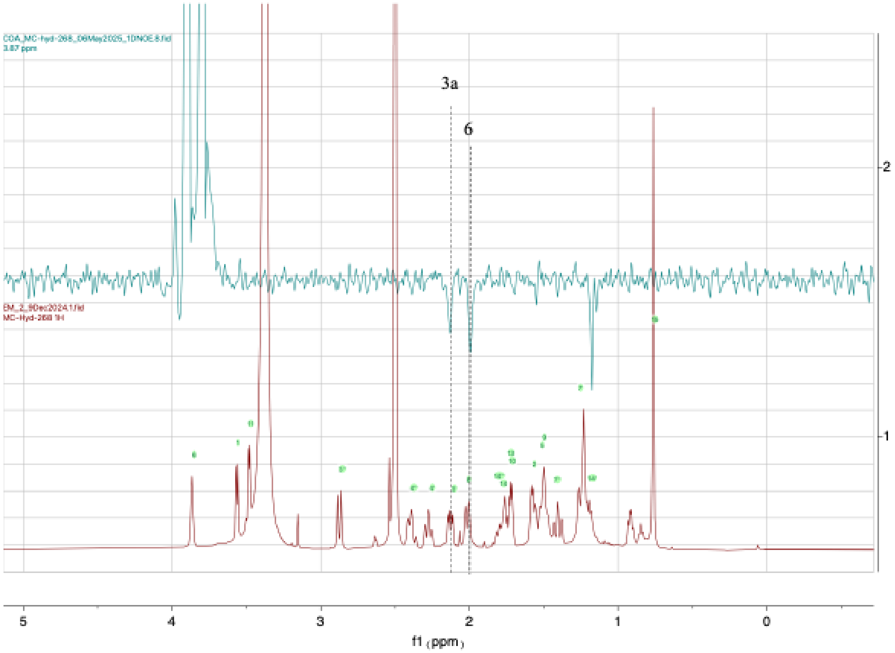

Figure S66. 1D NOE spectra for andrognathanol hydrate (**21**) (600MHz, d_6-DMSO). 96 scans, irradiating 3.87 ppm.

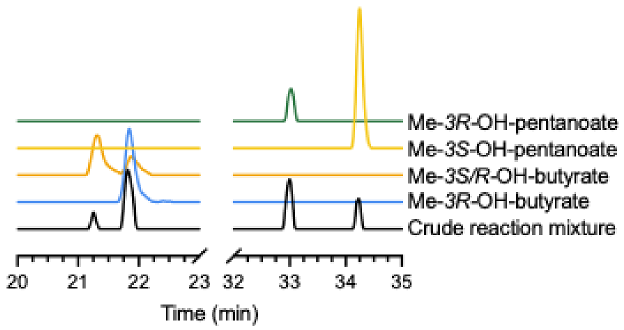

Figure S67. Ester side chain reactions stacked chromatograms of the methylated hydrolysate of crude extract along with Me-OH-pentanoate and Me-OH-butyrate standards, confirming the incorporation of both (*R*)- and (*S*)-Me-OH-pentanoate and (*R*)- and (*S*)-Me-OH-butyrate.

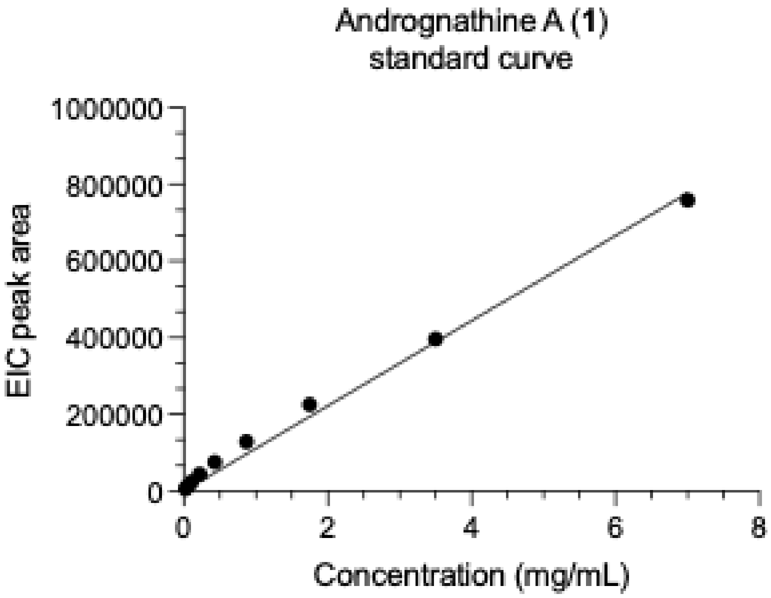

Figure S68. Absolute quantification of andrognathine A (**1**). The linear line was fitted to the LR-LCMS EIC peak area ($[M+H]^+$ 394.3 *m/z*) at eight concentrations (3.5, 1.75, 0.87, 0.43, 0.22, 0.11, 0.050, and 0.025 mg/mL). The line of best fit is y= 111000 (x), R^2= 0.99.

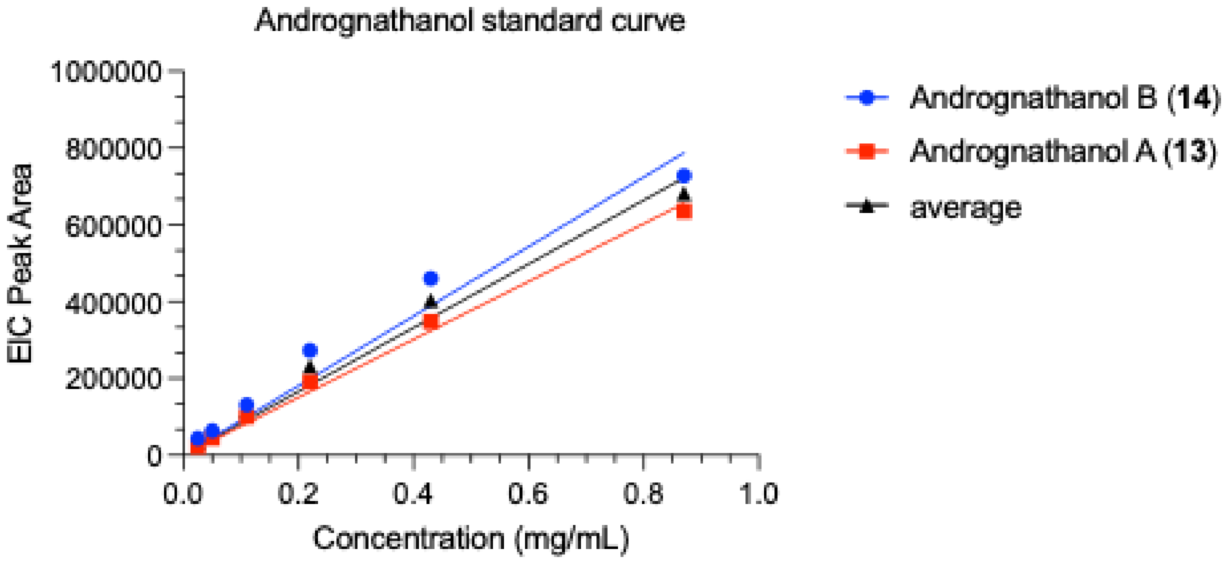

Figure S69. Absolute quantification of andrognathanol A (**13**) and B (**14**). The linear line was fitted to the LR-LCMS EIC peak area ($[M+H]^+$ 410.3 and 424.3 *m/z*) at six concentrations (0.87, 0.43, 0.22, 0.11, 0.050, and 0.025 mg/mL). The line of best fit for each is y= 685000 (x), R^2 = 0.99 (**13**); y= 789000 (x), R^2 = 0.99 (**14**). The lines are very similar, thus indicating similar ionization efficiencies. The lines were averaged to yield a best fit of y= 737000 (x), R^2 = 0.99.

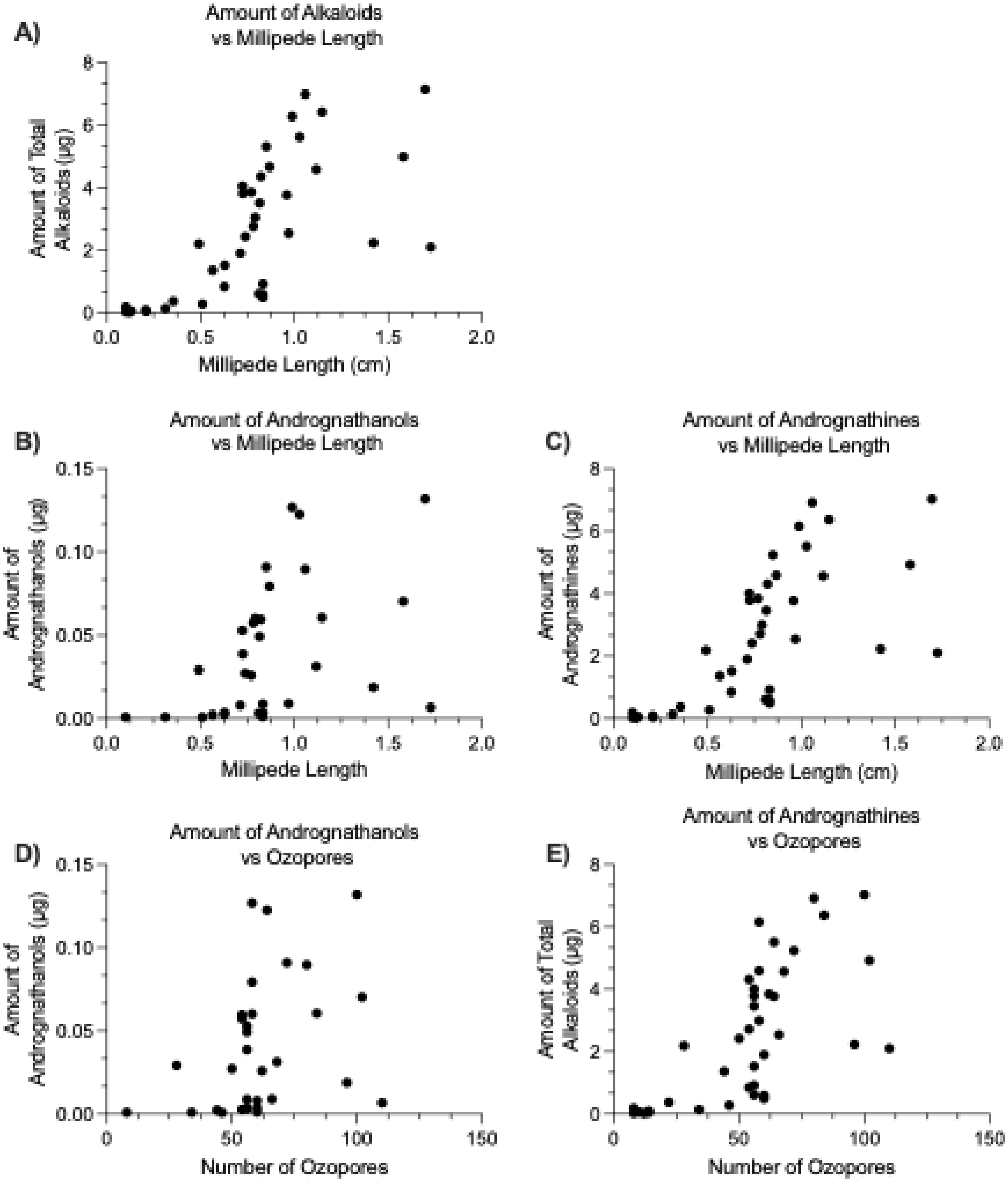

Figure S70. Alkaloids compared to length and number of ozopores. **A)** Amount of alkaloids extracted from the concentration curves compared to the length of the millipede. **B)** Amount of andrognathanols compared to the length of the millipedes. **C)** Amount of andrognathines compared to the millipede length. **D)** Amount of andrognathanols compared to the number of ozopores per millipede. **E)** Amount of andrognathines compared to the number of ozopores per millipede.

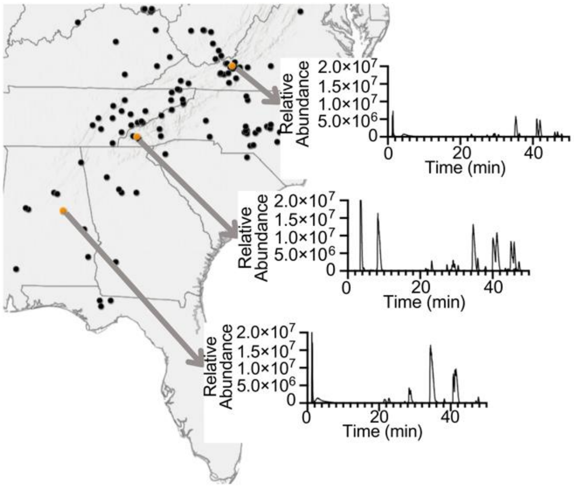

Figure S71. LR-LCMS analysis of *A. corticarius* samples collected in distinct geographical locations. Alabama, North Carolina and Virginia all contain both classes of compounds and do not show major differences in the composition.

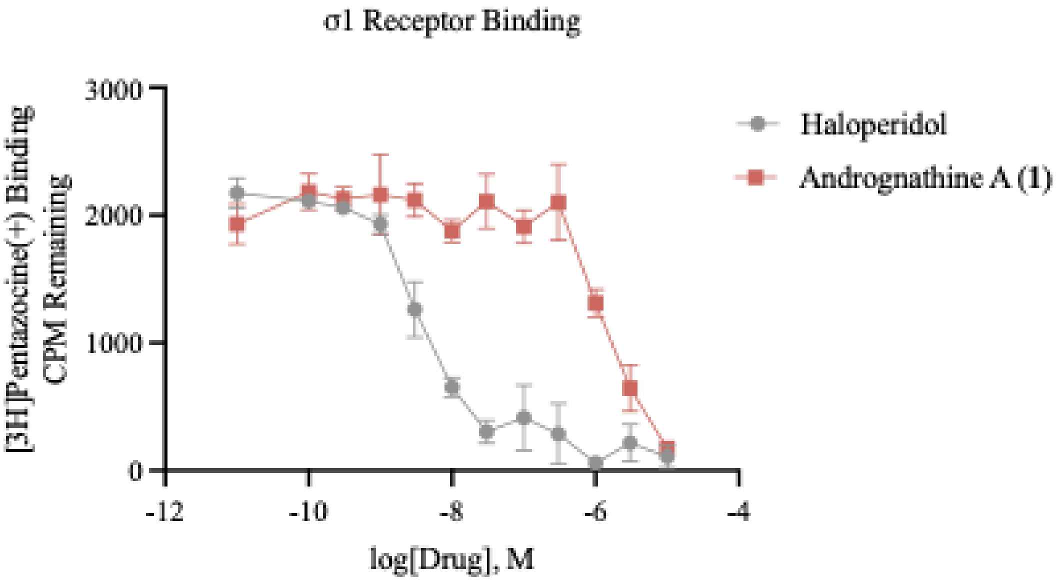

Figure S72. σ_1 Receptor Binding. Secondary screening of andrognathine A (**1**), and positive control Haloperidol.

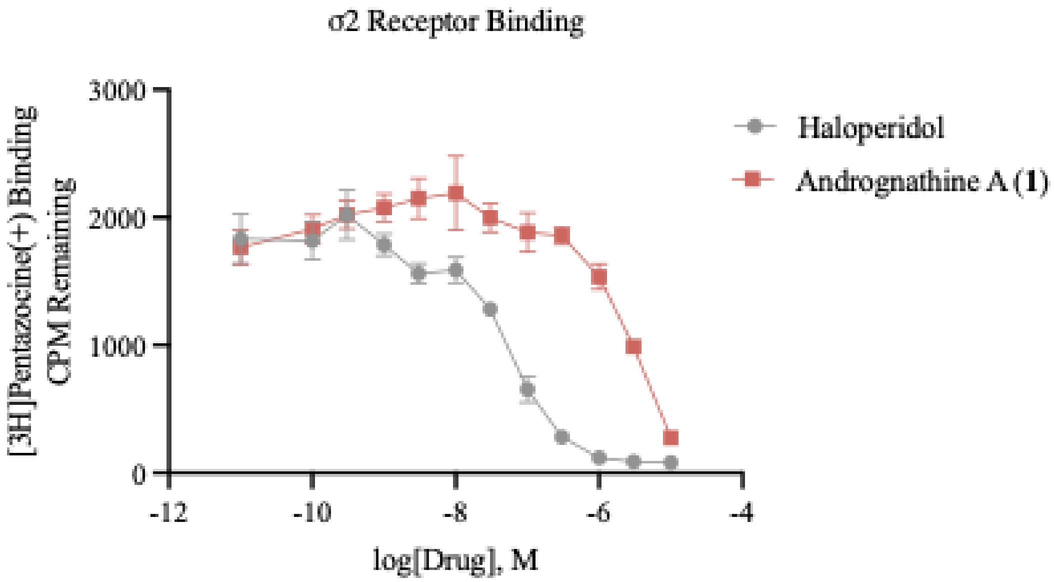

Figure S73. σ_2 Receptor Binding. Secondary screening of andrognathine A (**1**), and positive control Haloperidol.

Table S2. NMR Spectroscopy Data for Andrognathine A (**1**) (600 MHz, d_6-DMSO, 25ºC)

Position	δ_C, type[a]	δ_H (J in Hz)	H2BC	HMBC	COSY	ROESY
1	19.8, CH_3	1.00, d, 5.2	2	2, 3, 9	2	3b, 10
2	32.5, CH	1.21, m	1, 9	4	1	4b, 9
3a	34.1, CH_2	1.21, m	4	4, 9	4	10
3b		1.58, m	4	9		1
4a	51.8, CH_2	1.85, m	3	3, 5, 8	3, 8	4b
4b		2.91, m	3	2, 3		2, 3b, 4a
5a	53.1, CH_2	1.96, m	6	4, 6	6	5b
5b		2.94, m	6	7, 8		5a, 6
6	20.5, CH_2	1.65, m	5, 7		5	5b, 7b
7a	29.7, CH_2	1.53, m	6, 8	6, 8		7b, 11
7b		2.03, m				7a, 6
8	64.3, CH	1.76, t, 10.2	7, 9	4	4, 9	4b, 9
9	47.1, CH	1.16, m	2, 8	2, 8, 10	8	2, 8, 10
10	70.1, CH	5.38, d, 9.3	11	2, 8, 9, 11, 12, 15		1, 3a, 9, 11, 13
11	76.1, CH_2	5.45, d, 9.3	10	10, 12, 13, 14, 20		7a, 7b, 10, 13, 14a
12	139.9, C					
13	17.5, CH_3	1.70, s	14	11, 12, 14	14	10, 11, 14b
14a	118.1, CH_2	5.04, s	11, 13	11, 12, 13	13	11
14b		5.10, s	11, 13	11, 13		13
15	172.3, C					
16a	33.2, CH_2	2.21, m	17	15, 17, 18, 19		16b
16b		2.25, m	17	15, 17, 18, 19		16a
17	26.5, CH_2	1.45, m	16, 18	16, 18, 19	18	18
18	21.6, CH_2	1.24, m	17, 19	16, 17, 19	17, 19	17
19	13.3, CH_3	0.87, m	18	17, 18	18	
20	171.8, C					
21a	35.3, CH_2	2.22, m	22	20, 22, 23		21b, 22
21b		2.27, m	22	20		21a
22	17.9, CH_2	1.53, m	21, 23	20, 21, 23	23	21a
23	21.9, CH_3	0.86, m	22	21, 22	22	

[a] δ_C obtained indirectly through gHSQC and gHMBC data.

Table S3. NMR Spectroscopy Data for Andrognathine B (**2**) (600 MHz, d_6-DMSO, 25°C)

Position	δ_C, type[a]	δ_H (J in Hz)	H2BC	HMBC	COSY	ROESY
1	19.7, CH_3	1.01, d, 5.2	2	2, 3, 9	2	10, 13
2	32.5, CH	1.20, m	1, 9		1	9
3a	34.2, CH_2	1.20, m	4	1, 2	3b, 4a	3b
3b		1.58, m			3a	3a
4a	51.8, CH_2	1.79, m	3		3a, 4b	4b
4b		2.88, m		1	4a	4a
5a	53.2, CH_2	1.91, m	6	4	5b, 6	5b
5b		2.90, m	6	8	5a, 6	5a, 6
6	20.4, CH_2	1.63, m	5, 7		5a, 5b	
7a	29.7, CH_2	1.51, m	6	8	7b	11
7b		2.02, m			7a, 8	
8	64.1, CH	1.68, m	9		7b, 9	
9	47.1, CH	1.12, m	2	2, 8	8	2, 10, 11, 13
10	70.1, CH	5.38, d, 9.7	11	2, 8, 9, 11, 15	11	1, 9, 13
11	75.9, CH	5.47, d, 9.7	10	10, 12, 13, 14, 19	10	7a, 9, 13
12	140.0, C					
13	17.5, CH_3	1.71, s		11, 12, 14	14	1, 9, 10, 11, 22
14a	118.1, CH_2	5.05, s		11, 13	13	
14b		5.11, s	13	11, 13		
15	171.8, C					
16a	35.2, CH_2	2.21, m		15, 17	17	16b, 17
16b		2.27, m	17	15, 17		16a
17	17.7, CH_2	1.51, m	16, 18	15, 16	16, 18	16a
18	13.2, CH_3	0.87, m	17	15, 16, 17	17	
19	171.8, C					
20a	35.2, CH_2	2.21, m		19, 21	21	20b
20b		2.27, m	21	19, 21	21	20a
21	17.7, CH_2	1.51, m	20, 22	19, 20	20, 22	
22	13.2, CH_3	0.87, m	21	15, 20. 21	21	13

[a]δ_C obtained indirectly through gHSQC and gHMBC data.

Table S4. NMR Spectroscopy Data for Andrognathine C (**3**) (600 MHz, d_6-DMSO, 25ºC)

Position	δ_C, type[a]	δ_H (J in Hz)	H2BC	HMBC	COSY	ROESY
1	19.6, CH_3	1.00, m	2	2, 3, 9	2	3b, 10
2	32.4, CH	1.20, m	1, 9		1, 3b	
3a	34.1, CH_2	1.20, m	4	9	3b, 4a	
3b		1.57, m			2, 3a	1
4a	51.7, CH_2	1.79, m	3		3a, 4b	4b
4b		2.87, m			4a	4a
5a	53.2, CH_2	1.90, m	6	4	5b, 6	5b
5b		2.90, m	6	7, 8	5a	5a
6	20.4, CH_2	1.64, m	5		5a, 7b	
7a	29.6, CH_2	1.51, m		8	7b	7b, 11
7b		2.02, m	8		6, 7a	7a
8	64.1, CH	1.68, m	7, 9	9	9	9
9	47.1, CH	1.12, t, 9.3	2, 8	2, 8	8	8, 10
10	70.1, CH	5.37, d, 8.4	11	2, 9, 11, 15	11	1, 9, 13
11	75.9, CH	5.47, d, 8.4	10	10, 12, 13, 14, 19	10	7a, 13
12	140.0, C					
13	17.5, CH_3	1.71, s		11, 12, 14	14b	10, 11
14a	118.1, CH_2	5.04, s		11, 12, 13		13
14b		5.10, s	13	11, 12, 13	13	13
15	173.2, C					
16a	26.7, CH_2	2.26, m	17	15, 17	16b, 17	16b
16b		2.30, m			16a	16a
17	8.8, CH_3	1.02, m	16	15, 16	16a	
19	171.9, C					
20	33.1, CH_2	2.26, m	21	19, 21, 22, 23	21	
21	26.3, CH_2	1.45, m	20, 22	19, 20, 22, 23	20, 22	
22	17.7, CH_2	1.51, m	21, 23	20, 23	21, 23	
23	13.3, CH_3	0.85, m	22	21, 22	22	

[a] δ_C obtained indirectly through gHSQC and gHMBC data.

Table S5. NMR Spectroscopy Data for Andrognathine D (**4**) (600 MHz, d_6-DMSO, 25°C)

Position	δ_C, type[a]	δ_H (J in Hz)	H2BC	HMBC	COSY
1	19.6, CH_3	1.01, m	2	2, 3	2
2	32.5, CH	1.20, m			1
3a	34.3, CH_2	1.20, m			3b
3b		1.57, m	2		3a, 4b
4a	51.8, CH_2	1.80, m		8	4b
4b		2.87, m			3b, 4a
5a	53.1, CH_2	1.92, m		4	5b
5b		2.90, m			5a
6	20.5, CH_2	1.63, m			
7	26.3, CH_2	1.52, m	6		8
8	64.1, CH	1.67, m			7, 9
9	47.1, CH	1.13, m	9	10	8
10	70.3, CH	5.37, d, 9.0	11	11 15, 16	11
11	75.9, CH	5.46, d, 9.0	10	10, 13, 19	10
12	140.4, C				
13	17.6, CH_3	1.71, s		11	14a, 14b
14a	117.9, CH_2	5.04, s		12, 13, 14	13
14b		5.10, s		12, 13, 14	13
15	172.2, C				
16a	35.4, CH_2	2.23, m		15, 17	16b
16b		2.26, m		15, 17	16a
17	17.5, CH_2	1.52, q, 7.1	16	16, 18	18
18	21.7, CH_3	0.87, m	17	16, 17	17
19	171.8, C				
20a	33.2, CH_2	2.22, m		19, 23, 21	20b
20b		2.27, m		19, 23, 21	20a
21	26.2, CH_2	1.46, t, 7.7	20		22
22	21.2, CH_2	1.27, m	21, 23	23	21, 23
23	13.1, CH_3	0.85, m	22	21, 22	22

[a]δ_C obtained indirectly through gHSQC and gHMBC data.

Table S6. NMR Spectroscopy Data for Andrognathine E (**5**) (600 MHz, d_6-DMSO, 25°C)

Position	δ_C, type[a]	δ_H (J in Hz)	H2BC	HMBC	COSY
1	19.8, CH_3	1.01, d, 5.4	2	3, 9	2
2	32.6, CH	1.20, m	1, 9		1, 3b
3a	34.3, CH_2	1.21, m	4		3b, 4a
3b		1.57, d, 9.1		5	2, 3a
4a	51.9, CH_2	1.79, t, 10.9	3, 5		3a, 4b
4b		2.87, m		8	4a
5a	53.3, CH_2	1.90, m	6	4	5b
5b		2.90, m		8	5a
6	20.5, CH_2	1.62, m	5	7	
7	17.9, CH_2	1.52, m	6		
8	64.3, CH	1.68, m	9		9
9	47.3, CH	1.13, m	2, 10	2, 10	8
10	70.3, CH	5.38, d, 9.2	11	2, 8, 9, 11, 15	11
11	76.1, CH	5.46, d, 9.2	10	10, 12, 13, 14, 19	10
12	139.9, C				
13	17.6, CH_3	1.70, s		11, 12, 14	
14a	118.1, CH_2	5.04, s		11, 12, 13	
14b		5.10, s		11, 12, 13	
15	171.2, C				
16a	35.4, CH_2	2.27, m	17	15, 16, 18	16b, 17
16b		2.22, m	17	15, 16, 18	16a
17	17.6, CH_2	1.52, m	16, 18	15, 16, 18	16a, 18
18	13.5, CH_3	0.88, m	17	17	17
19	172.2, C				
20a	42.6, CH_2	2.11, m		19, 21	
20b		2.15, m		19, 21	
21	21.5, CH_3	0.88, m		20	

[a]δ_C obtained indirectly through gHSQC and gHMBC data.

Table S7. NMR Spectroscopy Data for Andrognathine F (**6**) (600 MHz, d_6-DMSO, 25°C)

**Position**	**δ_C, type[a]**	**δ_H (J in Hz)**	**HMBC**
1	19.8, CH_3	1.01, m	2
2	32.5, CH	1.20, m	
3a	34.4, CH_2	1.20, m	
3b		1.57, m	9
4a	52.0, CH_2	1.80, m	
4b		2.87, m	7, 8
5a	53.2, CH_2	1.91, m	4
5b		2.90, m	
6	20.5, CH_2	1.63, m	
7a	29.9, CH_2	1.50, m	8
7b		2.02, m	
8	64.1, CH	1.68, m	
9	47.3, CH	1.12, m	2, 9
10	70.3, CH	5.36, m	8, 11
11	76.1, CH	5.47, m	10, 12, 13, 14
12	139.9, C		
13	17.7, CH_3	1.70, s	11, 12, 14
14a	118.1, CH_2	5.05, s	11, 12, 13
14b		5.11, s	
15	173.1, C		
16	26.9, CH_2	2.27, m	17
17	9.09, CH_3	1.01, m	10, 16
18	171.7, C		
19a	35.3, CH_2	2.20, m	18, 20, 21
19b		2.27, m	
20	17.9, CH_2	1.50, m	19, 21
21	13.5, CH_3	0.85, m	19, 20

[a]δ_C obtained indirectly through gHSQC and gHMBC data.

Table S8. NMR Spectroscopy Data for Andrognathine Acetonide (**19**) (500 MHz, d_6-DMSO with fuming TFA, 25ºC)

Position	δ_C, type[a]	δ_H (J in Hz)	HMBC	COSY	ROESY
**1**	18.8, CH_3	1.05, d, 5.8	2, 9	2	11
**2**	31.9, CH	1.66, m		1	
**3**	28.7, CH_2	1.21, m			
**4a**	49.7, CH_2	3.48, m			
**4b**		2.91, m			
**5a**	50.8, CH_2	3.47, m		5b, 6a	
**5b**		2.92, m		5a, 6b	
**6a**	19.5, CH_2	1.88, m		5a, 6b, 7a	
**6b**		1.95, m		5b, 6b, 7b	
**7a**	27.4, CH_2	2.26, m		6a, 8	
**7b**		1.74, m		6b, 8	
**8**	65.4, CH	3.10, m		7a, 7b, 9	
**9**	45.0, CH	1.63, m		8	
**10**	76.6, CH	4.11, d, 8.3		11	
**11**	83.2, CH	3.41, t, 7.4		10, 12	
**12**	31.6, CH	1.72, m		11, 13, 14	10, 13, 14, 16
**13**	18.5, CH_3	0.93, d, 6.4	11, 12, 14	12	12
**14**	18.9, CH_3	0.84, d, 5.8	11, 12, 13	12	12
**15**	107.9, C				
**16**	27.1, CH_3	1.34, s	15, 17		10, 12
**17**	26.3, CH_2	1.27, s	15, 16		

Table S9. NMR Spectroscopy Data for Andrognathanol A (**13**) (600 MHz, d_6-DMSO, 25ºC)

Position	δ_C, type[a]	δ_H (J in Hz)	H2BC	HMBC	COSY	ROESY
1	75.4, CH	3.60, d, 5.5	2	2, 3, 11,13	2	13a
2	43.4, CH	1.70, m	1, 3, 10	3, 9, 10	1, 3, 10	3a, 10, 11
3a	55.4, CH_2	2.15, m	2	1, 2, 4, 8	2, 3b, 4b	3b, 8, 5
3b		2.94, d, 10.7	2	1, 2, 8, 10	3a	2, 3a, 4b
4a	49.5, CH_2	2.13, m	5	3, 5	4b, 5	4b
4b		2.55, d, 12.0	5	6, 8	4a, 5	3b, 4a, 5
5a	28.8, CH_2	1.66, m	4, 6	7	4a, 6	5,<br>4a, 4b, 6
6	67.7, CH	4.92, s	5, 7	4, 8	5, 7a	5, 7a, 7b
7a	32.0, CH_2	1.43, m	6, 8	5, 6	6, 7a	6, 7b, 8
7b		1.53, m	6, 8	8	8, 7b	6, 7a
8	57.6, CH	1.90, m	7, 9	7, 8, 9, 10, 14	9, 14a	3a, 7b, 9
9	30.3, CH	1.49, m	10, 14	2, 10	8	8, 10, 14a
10	40.8, CH	2.01, q, 4.3	2, 9, 11	1, 2, 11, 12, 14	2, 9, 11	2, 8, 9, 11
11	78.0, CH	4.60, d, 4.4	10	9, 12, 15, 19	10	2, 10, 15
12	42.6, C					
13a	30.6, CH_2	1.14, dd, 8.6, 12.5	14	1, 11, 12, 14, 15	13b, 14b	13b
13b		1.73, m	14	1, 11, 12, 14	13a	13a
14a	16.9, CH_2	1.30, pent, 6.7	13	10, 12, 13	9, 14b	13a, 13b, 14b
14b		1.91, m	13	8, 13	9, 13a, 14a	1, 14a
15	19.4, CH_3	0.78, s		1, 11, 12, 15	13a	11
16	172.9, C					
17	26.9, CH_2	2.29, q, 7.3	18	16, 18	18	18
18	8.8, CH_3	1.01, t, 7.4	17	16, 17	17	17
19	171.1, C					
20	44.1, CH_2	2.37, m	21	19, 21, 22	21	21, 22
21	63.2, CH	4.01, sext, 6.4	20, 22	19, 20	20, 22	20, 22
22	23.2, CH_3	1.10, d, 6.0	21	20, 21	21	20

[a]δ_C obtained indirectly from gHSQC and gHMBC experiments.

Table S10. NMR Spectroscopy Data for Andrognathanol B (**14**) (600 MHz, d_6-DMSO, 25°C)

Position	δ_C, type[a]	δ_H (J in Hz)	H2BC	HMBC	COSY	ROESY
1	75.5, CH	3.60, d, 5.7	2	2, 3, 11, 13	2	3b, 14b
2	43.5, CH	1.70, m	1, 3, 10	1, 9, 10, 12	1	3a, 10, 11
3a	55.4, CH_2	2.16, m	2	1, 2, 4	3b	4b, 8
3b		2.94, d, 12.4		1, 2, 8, 10	3a	4a, 4b
4a	49.6, CH_2	2.14, m	5		4b	3b, 5
4b		2.56, m	5	3, 6	4a, 5	3a, 3b
5	28.8, CH_2	1.66, m	4, 6		4, 6	4b, 6
6	67.4, CH	4.91, s	5, 7	4, 8	5, 7a	5, 7a, 7b
7a	32.1, CH_2	1.43, m	6, 8	6	7b	6, 7b, 8
7b		1.54, m	6, 8	8	7a, 8	6, 7a
8	57.7, CH	1.91, m	7, 9	9, 14	7b	9, 10, 11
9	30.3, CH	1.50, m	8, 10	8	10	8, 10
10	40.8, CH	2.02, m	2, 9, 11	1, 12	9, 11	2, 9, 11
11	78.1, CH	4.60, d, 4.6	10	12, 13, 15	10	2, 10, 15
12	42.4, C					
13a	30.6, CH_2	1.14, m	14	9, 11, 12, 14, 15	13b, 14b	13b, 15
13b		1.72, m	14	12	13a	13a, 14b
14a	16.9, CH_2	1.29, m	13	8, 10, 11, 13	14b	14b
14b		1.89, m	13	8, 13	13a, 14a	13b, 14a
15	19.4, CH_3	0.78, s		1, 11, 12, 13		11, 13a
16	172.9, C					
17	26.9, CH_2	2.28, m	18	16, 17	18	18
18	8.8, CH_3	1.01, t, 7.4	17	16, 17	17	17
19	171.3, C					
20a	42.1, CH_2	2.31, m	21	19, 21, 22	20b, 21	20b
20b		2.41, d, 5.6			20a, 21	20a
21	68.3, CH	3.76, m	20, 22	19, 23	20a, 22	22
22	29.5, CH_2	1.36, m	21, 23	20, 21, 23	23	21
23	9.6, CH_3	0.85, t, 7.4	22	21, 22	22	

[a]δ_C obtained indirectly through gHSQC and gHMBC data.

Table S11. NMR Spectroscopy Data for Andrognathanol C (**15**) (600 MHz, d_6-DMSO, 25°C)

Position	δ_C, type[a]	δ_H (J in Hz)	H2BC	HMBC	COSY	ROESY
1	75.5, CH	3.60, d, 5.9	2	2, 3, 11, 13	2	13a
2	43.6, CH	1.70, m	1, 3, 10	1, 10	1, 3a	3a, 10, 11
3a	55.4, CH_2	2.15, m	2	1, 4	2, 3b	2, 3b
3b		2.92, d, 12.1		1, 2, 8, 10	3a	3a, 4b
4a	49.5, CH_2	2.13, m	5		4b, 5b	4b
4b		2.54, m	5	6, 8	4a	3b, 4a, 5
5	28.7, CH_2	1.67, m	4, 6	4, 6, 7	4a, 6	4b, 6
6	67.4, CH	4.91, s	5	4, 8	5, 7a	5, 7a, 7b
7a	32.1, CH	1.43, m		5	6, 7b	6, 7b, 8
7b		1.54, m	8	8	7a, 8	7a, 6
8	57.6, CH	1.90, m	7, 9	9, 14	7b	1, 7a
9	30.5, CH	1.46, m	14			10
10	40.9, CH	2.02, m	2, 9, 11	1, 11, 12, 14	11	2, 9, 11
11	78.1, CH	4.63, d, 4.7	10	9, 12, 15	10	2, 10, 15
12	42.4 C					
13a	30.6, CH_2	1.15, m	14	1, 12, 14, 15	13b	13b
13b		1.71, m	14	1, 12, 14	13a, 14b	13a
14a	16.9, CH_2	1.30, m	9, 13	9, 10, 12, 13	14b	14b
14b		1.91, m	13	8, 13	14a	14a
15	19.5, CH_3	0.78, s		1, 11, 12, 13		11
16	172.1, C					
17	42.7, CH_2	2.21, m	18	16, 17	18	18, 19
18	25.1, CH	1.97, m	17, 18	17, 19	17, 19	17, 19
19	21.9, CH_3	0.91, d, 6.6	18	17, 18	18	17, 18
20	172.9, C					
21	26.9, CH_2	2.29, q, 7.5	22	20, 22	22	22
22	8.8, CH_3	1.01, t, 7.5	21	20, 21	21	21

[a]δ_C obtained indirectly through gHSQC and gHMBC data.

Table S12. NMR Spectroscopy Data for Andrognathanol D (**16**) (600 MHz, d_6-DMSO, 25ºC)

**Position**	**δ_C, type[a]**	**δ_H (J in Hz)**	**H2BC**	**HMBC**
1	75.6, CH	3.60, d, 6.1		3, 11, 13
2	43.6, CH	1.70, m		1, 12
3a	55.6, CH_2	2.16, m		
3b		2.92, d, 11.7		1, 2, 8, 10
4a	49.6, CH_2	2.13, m		
4b		2.55, m		
5	28.8, CH_2	1.66, m		
6	67.5, CH	4.91, s		
7a	32.2, CH_2	1.43, m		
7b		1.52, m		
8	57.8, CH	1.91, m		
9	30.7, CH	1.45, m		
10	41.0, CH	2.01, m		
11	78.2, CH	4.62, d, 4.6		9
12	42.4, C			
13a	30.7, CH_2	1.15, m		
13b		1.70, m		12
14a	17, CH_2	1.30, m		10, 12, 13
14b		1.91, m		
15	19.6, CH_3	0.78, s		1, 11, 12, 13
16	172.9, C			
17	27, CH_2	2.29, q, 7.7	18	16, 18
18	9.0, CH	1.01, t, 7.7	17	16, 17
19	172.2, C			
20	42.9, CH_2	2.21, dd, 4.5, 2.4		19, 21, 22
21	25.2, CH	1.99, m		22
22	22.0, CH_3	0.90, d, 6.8	21	20, 21

[a]δ_C obtained indirectly through gHSQC and gHMBC data.

Table S13. NMR Spectroscopy Data for Andrognathanol Hydrate (**21**) (500 MHz, d_6-DMSO, 25°C)

Position	δ_C, type[a]	δ_H (J in Hz)	COSY	HMBC	ROESY
1	76.3, CH	3.57, d 6.0	2, 15	2, 11, 13	3b, 14b, 15
2	44.0, CH	1.72, m	1, 3a, 3b, 10	1, 9, 10	3a, 9, 10, 11
3a	56.1, CH_2	2.11, dd 5.4, 11.8	2, 10	1, 2, 4, 8	2
3b		2.86, d 11.8	2		1, 4b
4a	49.4, CH_2	2.26, t 13.1	5a, 5b		
4b		2.38, t 11.7	5a, 5b		3b, 5b
5a	31.9, CH_2	1.50, m	4a, 4b, 6		7a
5b		1.53, m	4a, 4b, 6		4b, 6
6	62.4, CH	3.86, m	5a, 5b, 7a, 7b	8	5b, 7a, 7b
7a	35.5, CH_2	1.26, m	6, 8		5a, 6, 8
7b		1.42, d 12.3	6, 8		6
8	57.4, CH	2.01, d 11.3	7a, 7b, 9		7a, 9, 10
9	30.5, CH	1.50, m	8, 10, 14a	2	8, 10, 14a
10	43.7, CH	1.57, m	2, 3a, 9, 11	2, 9, 11, 14	2, 8, 9, 11
11	75.8, CH	3.48, d 4.5	10, 13a, 15	12, 13, 15	2, 10, 15
13a	29.9, CH_2	0.91, m	11, 14a, 14b	11, 12	
13b		1.73, m			
14a	17.4, CH_2	1.18, m	9, 13a		9
14b		1.81, m	13a		1
15	19.7, CH_3	0.76, s	1, 11	1, 11, 12, 13	1, 11

[a]δ_C obtained indirectly through gHSQC and gHMBC data.

Table S14. Quantification of alkaloids produced by *A. corticarius*

Millipede	Ozopores	Length (cm)	Amount of andrognathines (µg)	Amount of andrognathanols (µg)	All alkaloids (µg)
Mil-1	56	0.809	0.605	0.0032	0.608
Mil-2	56	0.723	4.01	0.053	4.06
Mil-3	100	1.696	7.03	0.132	7.16
Mil-4	102	1.579	4.92	0.070	4.99
Mil-5	110	1.726	2.09	0.0067	2.10
Mil-6	60	0.712	1.90	0.0079	1.91
Mil-7	58	0.79	2.99	0.060	3.05
Mil-8	80	1.058	6.91	0.090	7.00
Mil-9	54	0.781	2.71	0.057	2.77
Mil-10	96	1.422	2.22	0.019	2.24
Mil-11	54	0.626	0.841	0.0027	0.844
Mil-12	22	0.355	0.369	ND	0.369
Mil-13	50	0.737	2.42	0.027	2.45
Mil-14	56	0.832	0.912	0.0087	0.921
Mil-15	60	0.831	0.592	0.0012	0.594
Mil-16	46	0.509	0.275	0.00084	0.276
Mil-17	84	1.148	6.37	0.061	6.43
Mil-18	56	0.628	1.52	0.0036	1.53
Mil-19	68	1.117	4.56	0.031	4.59
Mil-20	28	0.492	2.18	0.029	2.21
Mil-21	44	0.565	1.36	0.0023	1.37
Mil-22	66	0.969	2.54	0.0089	2.55
Mil-23	64	0.959	3.77	ND	3.77
Mil-24	58	0.867	4.59	0.079	4.67
Mil-25	56	0.814	3.46	0.049	3.51
Mil-26	58	0.988	6.15	0.127	6.28
Mil-27	56	0.725	3.78	0.039	3.82
Mil-28	72	0.849	5.24	0.091	5.33
Mil-29	62	0.769	3.84	0.0260	3.87
Mil-30	60	0.832	0.490	0.0035	0.493
Mil-31	64	1.028	5.51	0.123	5.63
Mil-32	8	0.103	0.025	ND	0.025
Mil-33	8	0.102	0.184	0.00089	0.184
Mil-34	14	0.21	0.045	ND	0.045
Mil-35	54	0.821	4.31	0.060	4.37
Mil-36	14	0.208	0.089	ND	0.090
Mil-37	12	0.119	0.021	ND	0.021
Mil-38	10	0.13	0.054	ND	0.054
Mil-39	34	0.313	0.137	0.00097	0.138

ND = not detected

Table S15. σ_1 Receptor Binding

	Haloperidol			Andrognathine A		
**log[Drug], M**	**Trial 1**	**Trial 2**	**Trial 3**	**Trial 1**	**Trial 2**	**Trial 3**
-11	2087	2124	2308	1845	2112	1837
-10	2122	2096	2112	2031	2307	2212
-9.52	2115	2010	2050	2039	2124	2224
-9	1895	1884	2014	1816	2438	2228
-8.52	1056	1490	1239	1989	2251	2115
-8	597	738	617	1775	1915	1937
-7.52	202	376	335	1862	2201	2260
-7	153	424	664	1794	1891	2051
-6.52	86	550	225	1867	1998	2436
-6	59	63	54	1240	1438	1258
-5.52	47	284	314	435	777	724
-5	12	127	186	149	195	174

Table S16. σ_2 Receptor Binding

	Haloperidol			Andrognathine A		
log[Drug], M	**Trial 1**	**Trial 2**	**Trial 3**	**Trial 1**	**Trial 2**	**Trial 3**
-11	1680	1771	2049	1917	1657	1722
-10	1795	1684	1977	1805	1905	2026
-9.52	2199	1817	2050	1894	2087	2085
-9	1842	1830	1687	2200	2005	2020
-8.52	1572	1477	1632	1975	2284	2190
-8	1493	1693	1582	2461	1882	2230
-7.52	1288	1305	1254	1952	2128	1917
-7	769	598	591	2060	1788	1802
-6.52	272	244	326	1911	1782	1882
-6	137	105	111	1424	1617	1570
-5.52	85	97	84	928	994	1046
-5	134	38	73	226	284	321

Table S17. PDSP Primary Screen at 10 μM

**Compound**	**Receptor**	**Trial 1**	**Trial 2**	**Trial 3**	**Trial 4**	**Trial 5**	**Average**	**Standard Deviation**
Andrognathine A (**1**)	σ_1R	88.9	88.3	86.8	86.4	87.6	87.6	1.02
	σ_2R	93.0	75.3	72.8	69.0	77.5	77.5	9.23
Andrognathine B (**2**)	σ_1R	59.0	15.3	23.8	11.8	27.5	27.5	18.7
	σ_2R	84.4	20.3	18.4	16.7	34.9	34.9	28.6
Andrognathanol A (**13**)	σ_1R	61.8	20.2	18.9	3.64	26.1	26.1	21.6
	σ_2R	55.2	8.4	8.5	0.50	18.1	18.1	21.6
Andrognathanol B (**14**)	σ_1R	38.0	15.1	12.3	3.84	17.3	17.3	12.6
	σ_2R	65.7	3.54	3.7	22.9	23.9	23.9	25.4
Andrognathanol C (**15**)	σ_1R	60.9	18.1	19.7	19.3	29.5	29.5	18.1
	σ_2R	94.3	13.5	24.8	28.5	44.8	41.2	31.7

Table S18. Nav1.5 Human Sodium Ion Channel Cell Based APC LeadHunter Assay

Compound	Concentration (μM)	Pulse 2	Pulse 1
**0.3% DMSO**		4.89178	-16.6
**Andrognathanol A (13)**	0.03	6.71373	-17.90
	0.1	15.3467	-7.21
	0.3	6.91311	-16.50
	1	8.93239	-4.26
	3	3.43145	-15.70
	10	4.32694	-19.90
**Andrognathine A (1)**	0.03	2.19656	-21.80
	0.1	3.87136	-18.50
	0.3	-2.87	-24.60
	1	5.69991	-15.80
	3	4.27218	-16.90
	10	7.73735	-17.40

Assay completed by Eurofins Discovery Services

Table S19. Nav1.8 Human Sodium Ion Channel Cell Based APC LeadHunter Assay

Compound	Concentration (μM)	Pulse 2	Pulse 1
**0.3% DMSO**		-2.65	5.09
**Andrognathanol A (13)**	0.03	-9.18	0.02
	0.1	-1.86	-10.00
	0.3	-11.00	-21.70
	1	-3.96	-8.35
	3	-22.80	-15.70
	10	-6.62	-5.76
**Andrognathine A (1)**	0.03	-0.12	-7.02
	0.1	-5.71	-3.99
	0.3	-4.29	2.00
	1	5.12	10.02
	3	-13.70	-7.71
	10	-11.80	-6.70

Assay completed by Eurofins Discovery Services

Table S20. XYZ Coordinates Andrognathanols

Conformer 1 (correct isomer)

O	-4.403298	-0.413901	-0.836904
H	-1.675253	-0.562249	-1.276543
H	0.658624	-1.039365	-1.861177
O	2.607962	-2.090710	0.012516
O	2.999502	2.453132	-0.850855
H	1.645216	1.126802	-2.245215
C	4.184934	0.482199	0.793258
C	1.897939	-0.019289	1.758849
C	0.374995	-0.101078	1.523216
C	2.403021	-0.772074	-0.563859
C	2.694341	0.328855	0.480512
C	2.027084	1.581340	-0.196369
C	-0.066584	-0.690227	0.152473
C	0.964067	-0.470193	-0.977605
C	1.091804	1.028109	-1.304488
C	-0.263289	1.704430	-1.519808
C	-2.543143	-0.401761	0.688958
C	-1.417550	-0.085121	-0.311463
N	-1.279951	1.378358	-0.506009
C	-2.552133	2.005059	-0.906360
C	-3.671262	1.748329	0.102135
C	-3.878182	0.249572	0.337656
C	3.407497	3.573587	-0.219121
C	4.375046	4.356182	-1.071371
O	3.030226	3.907718	0.897302
C	2.817375	-3.125947	-0.826508
C	3.048597	-4.408806	-0.068115
O	2.825810	-3.021993	-2.046891
H	-5.233299	0.031153	-1.078917

H	4.566667	-0.441466	1.241863
H	4.769264	0.685935	-0.109402
H	4.359216	1.294290	1.507041
H	2.279536	-0.968117	2.148312
H	2.102451	0.738967	2.524202
H	-0.043347	0.905318	1.608742
H	-0.075410	-0.685808	2.331788
H	3.069190	-0.678986	-1.425925
H	1.500154	2.184909	0.541226
H	-0.215943	-1.771587	0.257876
H	-0.139039	2.794143	-1.543000
H	-0.620592	1.404886	-2.524202
H	-2.667003	-1.489351	0.750613
H	-2.262840	-0.050092	1.686820
H	-2.381314	3.083544	-1.000890
H	-2.866427	1.643350	-1.903278
H	-4.601101	2.208309	-0.255146
H	-3.423946	2.219580	1.061428
H	-4.582594	0.100905	1.167703
H	5.233300	3.729600	-1.331237
H	3.887587	4.653661	-2.004798
H	4.711851	5.241748	-0.532470
H	2.225465	-4.587204	0.629512
H	3.133825	-5.241748	-0.766035
H	3.968632	-4.325688	0.518986

Conformer 2 (enantiomer)

O	-4.403298	0.413901	-0.836904
H	-1.675253	0.562249	-1.276543
H	0.658624	1.039365	-1.861177
O	2.607962	2.090710	0.012516
O	2.999502	-2.453132	-0.850855
H	1.645216	-1.126802	-2.245215
C	4.184934	-0.482199	0.793258
C	1.897939	0.019289	1.758849
C	0.374995	0.101078	1.523216
C	2.403021	0.772074	-0.563859
C	2.694341	-0.328855	0.480512
C	2.027084	-1.581340	-0.196369
C	-0.066584	0.690227	0.152473
C	0.964067	0.470193	-0.977605
C	1.091804	-1.028109	-1.304488
C	-0.263289	-1.704430	-1.519808
C	-2.543143	0.401761	0.688958
C	-1.417550	0.085121	-0.311463
N	-1.279951	-1.378358	-0.506009
C	-2.552133	-2.005059	-0.906360
C	-3.671262	-1.748329	0.102135
C	-3.878182	-0.249572	0.337656
C	3.407497	-3.573587	-0.219121
C	4.375046	-4.356182	-1.071371
O	3.030226	-3.907718	0.897302
C	2.817375	3.125947	-0.826508
C	3.048597	4.408806	-0.068115
O	2.825810	3.021993	-2.046891
H	-5.233299	-0.031153	-1.078917

H	4.566667	0.441466	1.241863
H	4.769264	-0.685935	-0.109402
H	4.359216	-1.294290	1.507041
H	2.279536	0.968117	2.148312
H	2.102451	-0.738967	2.524202
H	-0.043347	-0.905318	1.608742
H	-0.075410	0.685808	2.331788
H	3.069190	0.678986	-1.425925
H	1.500154	-2.184909	0.541226
H	-0.215943	1.771587	0.257876
H	-0.139039	-2.794143	-1.543000
H	-0.620592	-1.404886	-2.524202
H	-2.667003	1.489351	0.750613
H	-2.262840	0.050092	1.686820
H	-2.381314	-3.083544	-1.000890
H	-2.866427	-1.643350	-1.903278
H	-4.601101	-2.208309	-0.255146
H	-3.423946	-2.219580	1.061428
H	-4.582594	-0.100905	1.167703
H	5.233300	-3.729600	-1.331237
H	3.887587	-4.653661	-2.004798
H	4.711851	-5.241748	-0.532470
H	2.225465	4.587204	0.629512
H	3.133825	5.241748	-0.766035
H	3.968632	4.325688	0.518986
